# Prospective Role of Peptide-Based Antiviral Therapy Against the Main Protease of SARS-CoV-2

**DOI:** 10.3389/fmolb.2021.628585

**Published:** 2021-05-10

**Authors:** Shafi Mahmud, Gobindo Kumar Paul, Suvro Biswas, Shamima Afrose, Mohasana Akter Mita, Md. Robiul Hasan, Mst. Sharmin Sultana Shimu, Alomgir Hossain, Maria Meha Promi, Fahmida Khan Ema, Kumarappan Chidambaram, Balakumar Chandrasekaran, Ali M. Alqahtani, Talha Bin Emran, Md. Abu Saleh

**Affiliations:** ^1^ Microbiology Laboratory, Department of Genetic Engineering and Biotechnology, University of Rajshahi, Rajshahi, Bangladesh; ^2^ Department of Genetic Engineering and Biotechnology, University of Rajshahi, Rajshahi, Bangladesh; ^3^ Department of Pharmacy, International Islamic University Chittagong, Chittagong, Bangladesh; ^4^ Department of Pharmacology, College of Pharmacy, King Khalid University, Abha, Saudi Arabia; ^5^ Department of Medicinal Chemistry, Faculty of Pharmacy, Philadelphia University-Jordan, Amman, Jordan; ^6^ Department of Pharmacy, BGC Trust University Bangladesh, Chittagong, Bangladesh

**Keywords:** SARS-CoV-2, COVID-19, M Pro, peptides, molecular dynamics, in silico

## Abstract

The recently emerged coronavirus (SARS-CoV-2) has created a crisis in world health, and economic sectors as an effective treatment or vaccine candidates are still developing. Besides, negative results in clinical trials and effective cheap solution against this deadly virus have brought new challenges. The viral protein, the main protease from SARS-CoV-2, can be effectively targeted due to its viral replication and pathogenesis role. In this study, we have enlisted 88 peptides from the AVPdb database. The peptide molecules were modeled to carry out the docking interactions. The four peptides molecules, P14, P39, P41, and P74, had more binding energy than the rest of the peptides in multiple docking programs. Interestingly, the active points of the main protease from SARS-CoV-2, Cys145, Leu141, Ser139, Phe140, Leu167, and Gln189, showed nonbonded interaction with the peptide molecules. The molecular dynamics simulation study was carried out for 200 ns to find out the docked complex’s stability where their stability index was proved to be positive compared to the apo and control complex. Our computational works based on peptide molecules may aid the future development of therapeutic options against SARS-CoV-2.

## Introduction

The novel coronavirus, currently known as SARS-CoV-2 (severe acute respiratory syndrome coronavirus-2), originated in Wuhan, Hubei Province, China, and then spread rapidly worldwide (Singhal, 2020; Wang et al., 2020a). SARS-CoV-2 is a virus with a high contagious property. As the virus is highly contagious, it spreads quickly to many other regions of China and other countries of the world within just 1 month (Luo et al., 2020). As of this study’s writing, 3,060,651 people have died so far in COVID-19, and confirmed cases are 143,663,539 (https://www.worldometers.info/coronavirus/#countries). This virus has spread worldwide, but Europe can be called the epicenter of COVID-19 because of its worse attack than China (Ceylan, 2020).

Later in December 2019, pneumonia-like symptoms of indecipherable etiology were confirmed in Wuhan, Hubei Province, China, where seafood in conjunction with wild and farmed animal species is vented in a wholesale seafood market, that is, preliminarily thought to be enacting the phenomenon of this worldwide outbreak (Zheng, 2020). As of this study’s writing, 2,600,162 people have died so far in COVID-19 and confirmed cases are 117,089,586 (https://www.worldometers.info/coronavirus/#countries). SARS-CoV-2 is a zoonotic transformation disease where the disease spreads from animal to human via the intermediate host (Dehelean et al., 2020). Bat-coronavirus (bat-nCoV) RaTG13 exerted near about 96% sequence identity with SARS-CoV-2 that sorely indicates bats are the main natural reservoir. In contrast, few criteria like clinical signs, histological changes, and circulating antibodies of Malayan pangolins (*Manis javanica*) partook 90.1% identity and 99% similarity of genome sequence SARS-CoV-2 implying Malayan pangolins as an immediate, intermediate host of deadly COVID-19 disease (Li et al., 2020; Zhao et al., 2020). The very first four cases of SARS-CoV-2 disease are directly in contact with the seafood market from where this disease spreads rapidly among humans by person-to-person contamination via several ways of transference, among which droplet transmission and respiratory droplets are the most causative reasons for transmitting infection toward noninfected people. When people are nearly connected within 1.5 m, there is a highly risky chance of droplet transmission by forming droplets of mucosae or conjunctiva of an infected person. In contrast, airborne transmission, fecal–oral transmission, and vertical transmissions are lower risky transmission mediums of emerging SARS-CoV-2 strain (Acuti Martellucci et al., 2020; Wang et al., 2020b).

Glycoprotein-associated homotrimeric spike protein (S) is a fusion protein that comprises the S1 and S2 subunits. For the S1 subunit, spike protein held a binding with receptor-binding domain (RBD) within the host cell contrary to a fusion between cellular and viral membranes for the S2 subunit. This type of binding cascade leads the viral genome to enter into the cell from which it reproduces countless viruses, and the engrossing structure of spikes plays the premier role in spreading infection (Chan et al., 2020; Lan et al., 2020; Yang et al., 2020). Angiotensin-converting enzyme 2, abbreviated as ACE2, is recognized as a well-known receptor of life-threatening SARS-CoV-2, and by binding to ACE2, spike protein–containing virus then enters into the mucosal endothelial cells. It requires 96 h to enter human respiratory epithelial cells and near about take 6 days for the entire cell line, and after contamination, interacting of saliva and respiratory secretions through coughing or sneezing or talking or singing, this virus can touch the nose, mouth, or eyes of a noninfected person from which virus spreads toward circumstances (Zhang et al., 2020b; Shang et al., 2020; WHO, 2020).

Coronaviruses contain enveloped, positive-sense, single-stranded RNA. They are also known as the largest RNA viruses because of their large genome size. These viruses range in size from (65–125 nm) in diameter as they are minute in size (Ge et al., 2020; Shereen et al., 2020). The SARS-CoV-2 belonged to the family Coronaviridae, which is in the order Nidovirales and Betacoronavirus genus (Zhong et al., 2003; Ge et al., 2020; Shereen et al., 2020). Seven coronaviruses can cause disease in the human body. The four human coronaviruses (HKU1, NL63, HCoV 229E, and OC43) are known to be globally endemic and cause damage to the respiratory tract of the elderly through infection of the upper portion of the tract. Alpha, beta, gamma, and delta—these are the four genera of coronaviruses (Chen et al., 2020; Ge et al., 2020; Wu et al., 2020a). As there are four subfamilies of coronaviruses, the alpha coronaviruses and beta coronaviruses possibly not only infect mammals but also originate from them. These mammals are specifically bats. Birds and pigs are the origins of another two subfamilies, gamma coronaviruses and delta coronaviruses. It is already known that coronaviruses have the largest genome and their genome size differs from 26 to 32 kb. Four leading structural genes encipher the membrane glycoprotein (M), the spike protein (S), nucleocapsid protein (N), and a small membrane protein (SM) (Velavan and Meyer, 2020; Zhou et al., 2020).

Since it has been predicted that SARS-CoV-2 originated from bats, the genome of this virus naturally matches about 96% of bats’ genome. This virus’s genome contains a single open reading frame (ORF) that encodes a polyprotein, cap structure at 5′, and a poly-A-tail at 3'. Cap structure and poly-A-tail are the two untranslated regions (UTRs). The genomic structure of SARS-CoV-2 starts from the 5′ end. Then, there are two viral replicases, open reading frame (ORF 1a and 1b). COVID-19 takes on a pandemic form in a very short time.

The main protease of SARS-CoV-2 (M^pro^) is a leading enzyme that plays a crucial role in regulating viral replication and transcription, the functioning of viral replicase enzymes and self-maturation, and in the acclimatization of the immune response in the host, among further fundamental actions for progressing the pathogen within a host cell (Dai et al., 2020; Joshi et al., 2020; Silva Andrade et al., 2020; Wu et al., 2020b; Zhang et al., 2020a). The SARS-CoV main protease is a 33.8 kDa protease (also known as the 3C-like protease or 3CL^pro^), a promising target for drug design due to its unexpected inhibitor mode binding site (Yang et al., 2003). Along with, SARS-CoV main protease has a pivotal role in mediating viral replication, multiplication, and transcription, as well as it is a key enzyme in the viral life cycle. On the point of being primarily inactive, the last C-terminal helix in domain III of M^pro^ monomer is involved in the dimerization of the main protease, and the derived homodimer is the active form of the enzyme which contains an intercommunicating attachment, chiefly between domain II of molecule A and the N-finger (NH_2_-terminal residues) of molecule B, aligned perpendicular to one another (Suárez and Díaz, 2020; Ullrich and Nitsche, 2020; Zhang et al., 2020a). In protomers, dimerization of the enzyme is mandatory for catalytic activity because the N-finger (residues 1–7) in domain I of one protomer intervenes with Glu166 of another protomer and thus assists in forming the S1 pocket of the substrate-binding site and the oxyanion loop as well in protomers. Emphatically, the Thr285Ala substitution marked in the SARS-CoV-2 M^pro^ concedes the two domains III to appear slightly closer, evolving higher catalytic efficiency (Anand et al., 2002; Yang et al., 2003; Suárez and Díaz, 2020; Zhang et al., 2020a).

Few vaccine candidates have been applied to nonhuman primates (NHPs) during trial phases (Dagotto et al., 2020). Some notable vaccine candidates are PiCoVacc (Wang et al., 2020c), DNA vaccines (Yu et al., 2020), RNA vaccines (Corbett et al., 2020), (mRNA-1273), and adenovirus-based vaccines ChAdOx1 (van Doremalen et al., 2020) and Ad26 (Mercado et al., 2020). Moreover, very few promising vaccine candidates have been showing good results during the clinical trial and early phase clinical trial (Dagotto et al., 2020; Folegatti et al., 2020; Jackson et al., 2020; Zhu et al., 2020).

## Materials and Methods

### Peptide and Protein Preparation

The antiviral database AVPdb (Qureshi et al., 2014) was utilized for screening peptides. Primarily, 88 peptide sequences were extracted from the database based on the activity against SARS-CoV-2. The peptide sequences were modeled before the peptide modeling. The PEP-FOLD-3 webtool (Maupetit et al., 2009; Thévenet et al., 2012) was used to model the peptide structure by utilizing amino acid sequences from the peptide. The three-dimensional protein structure of the main protease from SARS-CoV-2 (6LU7) was taken from the protein data bank database (RCSB) (Berman et al., 2000). The protein structure was initially cleaned; water molecules and heteroatoms were removed. The cleaned protein structure was subjected to an energy minimization process in YASARA (Krieger et al., 2013) by employing the YASARA force field. The minimized structure was saved for further docking and simulation study.

### Active Site Prediction

The CASTp (Computed Atlas of Surface Topology of protein) provides protein pockets and the protein's buried interface by solvent-accessible surface and molecular surface models. This webtool (www.scts.bioe.uic.edu/castp/) can precisely predict the functional and surface features. This program’s catalytic sites/active sites of the protein model can be identified (Binkowski et al., 2003).

### Molecular Docking

The peptides were initially docked in PatchDock (Schneidman-Duhovny et al., 2005) tools by targeting the main protease of SARS-CoV-2. The protein structure was used as a receptor, and the peptides were input as ligand molecules. The best 10 conformations of each peptide–protein complex were further docked in FireDock tools (Mashiach et al., 2008). The peptides were further docked in ClusPro (Kozakov et al., 2017) tools to maintain the accuracy in the docking study.

### SAR Analysis of Peptides

The ProtParam (Garg et al., 2016) tools were initially used for peptides properties calculation. The best 15 peptides based on binding energy were considered for the SAR analysis, where acidic, basic, polar, nonpolar amino acid, net charge at pH, molecular weight, and volume were considered. Furthermore, the principal component analysis of the peptide properties was also calculated to further explore the structural variance.

### Molecular Dynamics Simulations

The molecular dynamics simulation study was employed in YASARA dynamics (Krieger et al., 2013) commercial package where AMBER14 force field was utilized. The co-crystalized protein complex of main protease (PDB ID: 6LU7) was used as control-1, whereas peptide molecules QYIKWPWYI and the main protease complex were used as the control-2 system. The peptide from control-2 has COVID-19-induced T cell recognition and currently in the preparatory stage of clinical trials (EudraCT 2020-002502-75, EudraCT 2020-002519-23) (Nelde et al., 2021). The ligand-free protein complex (apo) was also included in this study to compare the protein's stability after binding with the protein molecules. The protein complex was initially cleaned, and the hydrogen bond network system was optimized along with the removal of the bumps from protein structure. The peptides and protein complexes were taken into the simulation box, and the total environment of the simulation system was neutralized by adding water molecules; 0.9% NaCl and the pH was set 7.4 (Krieger and Vriend, 2015). The temperature of the simulation system was 310 k, and the simulation temperature was maintained with the aid of the Berendsen thermostat. The TIP3P water model was used, and the cell density was set as 1.012 gm/cm^3^. The simulation cell box was bigger than the peptide and epitope complexes by 20 Å so that the complex can move freely within the complex (Land and Humble, 2018). The long-range electrostatic interactions were calculated through PME or the particle mesh Ewald method (Krieger et al., 2006). The simulation was initially optimized with the help of steepest gradient approaches using 5,000 cycles. The simulation was run by using 1.25 fs time step. The trajectories were saved after every 100 ps to analyze the root mean square deviation, root mean square fluctuations, hydrogen bond, radius of gyration, and solvent accessible surface area (Bappy et al., 2020; Khan et al., 2020; Islam et al., 2020; Mahmud et al., 2020a; Mahmud et al., 2020b; Mahmud et al., 2021b; Swargiary et al., 2020; Chowdhury et al., 2021; Rakib et al., 2021; Uddin et al., 2021).

## Results and Discussion

### Active Site Prediction

The CASTp webserver helps to identify the active groove of the main protease from SARS-CoV-2. About twenty-five active points were determined in CASTp tools; Arg 188, Asn119, Asn142, Asp187, Cys44, Cys145, Gln189, Gln192, Glu166, Gly143, His41, His164, Leu27, Leu167, Met49, Met165, Phe181, Pro168, Pro52, Thr24, Thr25, Thr26, Thr45, Tyr54, and Val186. These amino acids from the main protease were carefully assessed and considered for the peptide screening process via molecular docking.

### Molecular Docking

The peptide sequences were modeled in PEP-FOLD server and incorporated in the Supplementary Figure S1. The docking energy from each peptide is documented in Supplementary Table S1. The most favorable binding energy found for P14, P39, P41, and P74 than other peptide models. The binding energy from FireDock server was found for best four peptides in between −55 and −60 kcal/mol. The FireDock energy for four peptides was as −58.45, −59.18, −55.13, and −59.16 kcal/mol, respectively, for P14, P39, P41, and P74 (Table 1). Moreover, to understand the binding with the main protease and peptide molecules, flexible docking approaches from ClusPro were also assessed. The binding pocket or targeted amino acid sequences were not specified for this docking study. However, strong binding energy was also found in ClusPro docking program. The P14, P39, P41, and P74 had binding energy in ClusPro program as −885.9, −839.2, −888.4, and −926.3 kcal/mol, respectively. The docking energy of the other peptides was reported in Supplementary Table S1.

**TABLE 1:** Docking score of the top four peptides based on binding energy. The binding energy was calculated through FireDock and ClusPro program.

Peptides ID	Sequence	FireDock score (kcal/mol)	ClusPro score (kcal/mol)
P14	YQDVNCTDVSTAIHADQLTP	−58.45	−885.9
P39	SVVPSKATWGFA	−59.18	−839.2
P41	ALNCYWPLNDYGFYTTTGIGYQPYRVVVLSFEL	−55.13	−888.4
P74	VVNIQKEIDRLNEVAKNLNESLIDLQELGKYEQYIKWPW	−59.16	−926.3

### Docking Interaction

A virtual screening process including molecular docking is regarded as a feasible approach for identifying reliable antiviral therapeutics among diverse peptide sequences available in the reliable online-based database. Notably, the interaction type, and the binding energy in conjunction with the bond distance of interaction between a protein substrate and a peptide ligand, can be evaluated through the utility of molecular docking. Therefore, screening a plausible peptide among numerous peptide sequences based on binding energy assessment is feasible within minimum time by running molecular docking to evaluate supreme peptide candidates according to the docking interaction. Moreover, diverse docking programs are compatible for engaging in the computational scheme to achieve accuracy (Shoichet, 2006; Sousa et al., 2010; Hengphasatporn et al., 2020; Maia et al., 2020). Consequently, a computational-based approach is considered a reliable technique for apprehending a repurposing antiviral peptide candidate against the SARS-CoV-2.

**FIGURE 1:**
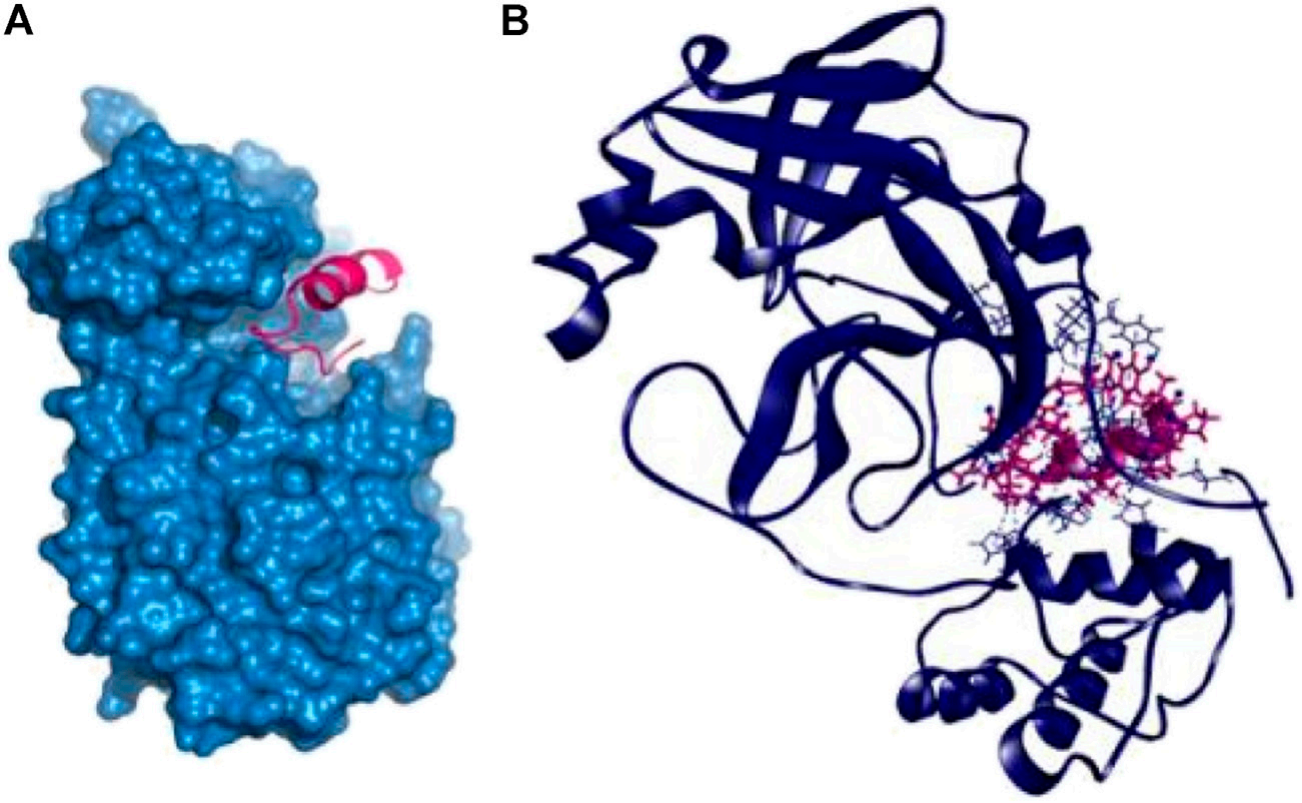
Docking interaction with P14 peptide and main protease enzyme of SARS-CoV-2 **(A**,**B)**.

The P14 peptide created eight hydrogen bonds with the main protease from SARS-CoV-2 at Lys102, Gln110, Arg298, Ser301, Ile152, Ser158, and Val297 positions where each of the amino acid residue has bond distance below 2.5 Å indicating stronger binding interactions. This complex had five more hydrophobic bonds at Phe294 (Pi-sulfur), Tyr154 (Pi–Pi T-shaped), Pro252 (alkyl), Ile249 (alkyl), and His246 (Pi-alkyl) residues (Table 2 and Figure 1).

**TABLE 2:** Nonbonded interaction of the top four peptides and main protease of the SARS-CoV-2, here, H, A, PA, PS, and PPT denote hydrogen bond, alkyl bond, Pi-alkyl bond, Pi-sulfur bond, and Pi–Pi T-shaped interactions, respectively.

Peptide	Amino acid	Bond type	Distance
P14	Lys102	H	1.95
	Asp153	H	1.92
	Gln110	H	2.02
	Arg298	H	2.15
	Ser301	H	1.99
	Ile152	H	2.15
	Ser158	H	2.59
	Val297	H	1.95
	Phe294	PS	2.78
	Tyr154	PPT	4.23
	Pro252	A	4.99
	Ile249	A	4.01
	His246	PA	5.26
P39	Asn142	H	2.05
	Thr24	H	1.68
	Glu166	H	2.55
	Asn142	H	1.46
	Asp187	H	2.97
	Gln189	H	2.80
	Cys145	A	3.63
	His41	PA	4.12
P41	Lys102	H	1.80
	Arg245	H	1.76
	Arg105	H	1.95
	Gln107	H	2.01
	Gln110	H	2.05
	Tyr154	H	1.86
	Ile249	H	2.05
	Asp153	H	2.01
	Gln107	H	2.53
	Thr198	H	2.41
	Phe294	PA	4.47
	Pro293	PA	4.19
	Arg105	PA	4.68
P74	Glu166	H	1.72
	Ser139	H	1.76
	Phe140	H	2.76
	Leu167	H	2.52
	Ile135	H	1.95
	Gln189	H	1.93
	Ser139	H	2.94
	Met165	H	2.65
	Pro168	H	2.25
	Asp187	H	3.05
	Cys145	PS	4.95
	Leu141	A	4.93

**FIGURE 2:**
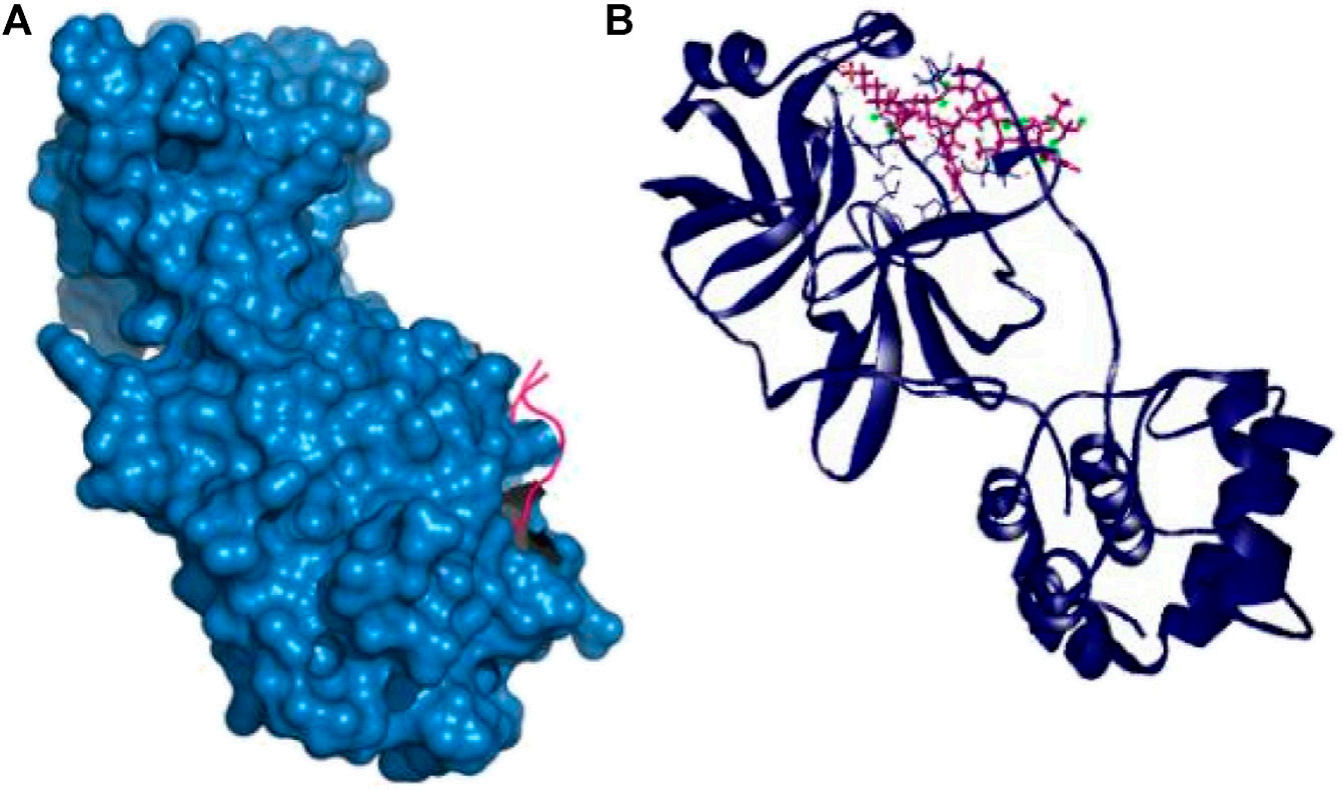
Nonbonded interaction with the main protease and P39 peptide **(A**,**B)**.

Therefore, the P39 complex stabilized by creating six hydrogen bonds at Asn142, Thr24, Glu166, Asn142, Asp187, and Gln189 positions. Two more hydrophobic bonds were also observed at Cys145 and His41 residues. Interestingly, all of the interacting residues, except Asp187, from P39 complex were in the active cavity of the main protease enzyme. These binding at the active groove may be responsible for the possible inhibition of the targeted protein (Table 2 and Figure 2).

**FIGURE 3:**
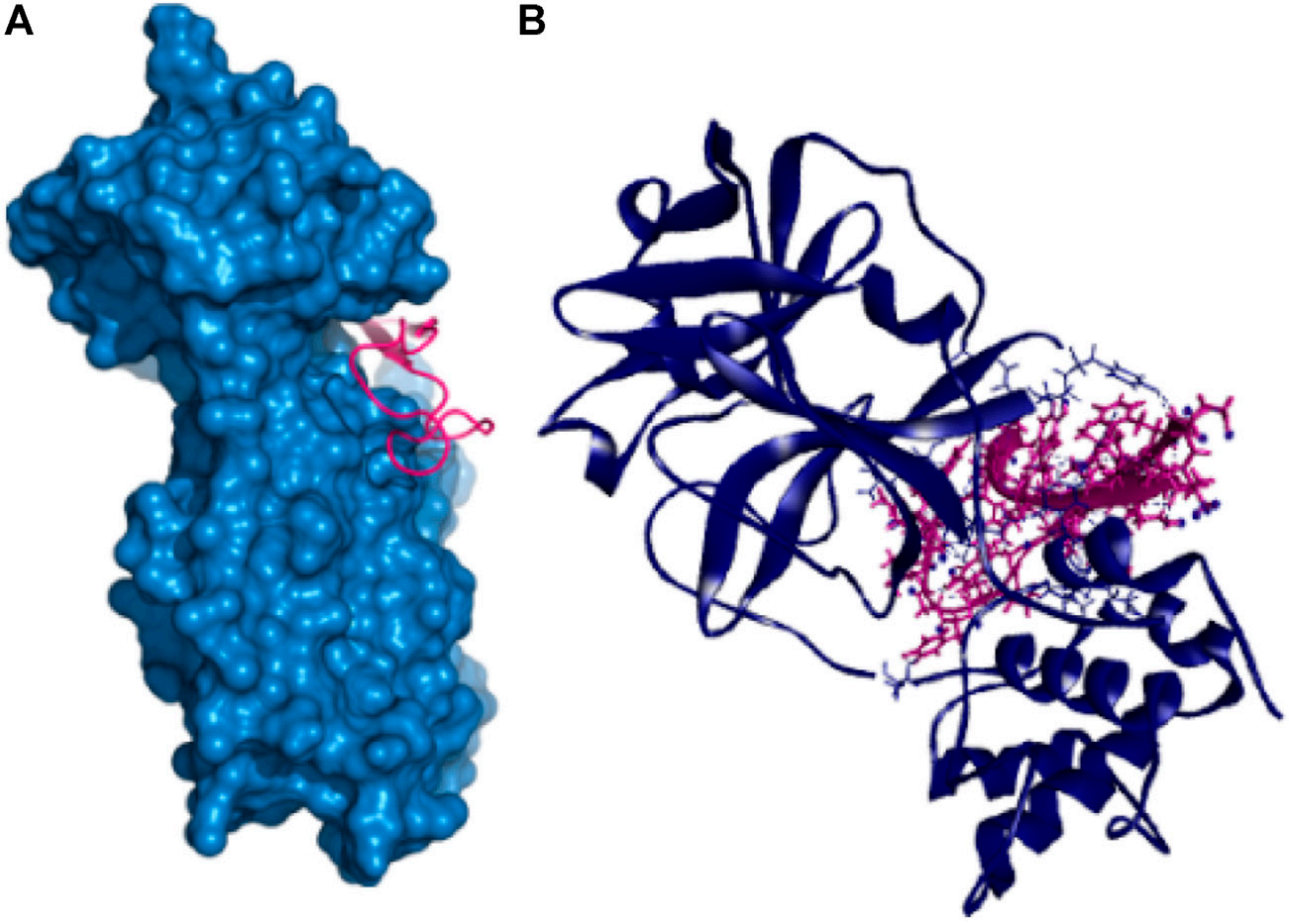
Non-covalent interactions of main protease and P41 peptide molecule **(A**,**B)**.

Also, the P41 peptide creates ten hydrogen bonds with the target protein at Lys102, Arg245, Arg105, Gln107, Gln110, Tyr154, Ile249, Asp153, Gln107, and Thr198 residues. This complex also exhibited three more hydrophobic bonds at Phe294, Pro293, and Arg105 (Table 2 and Figure 3).

**FIGURE 4:**
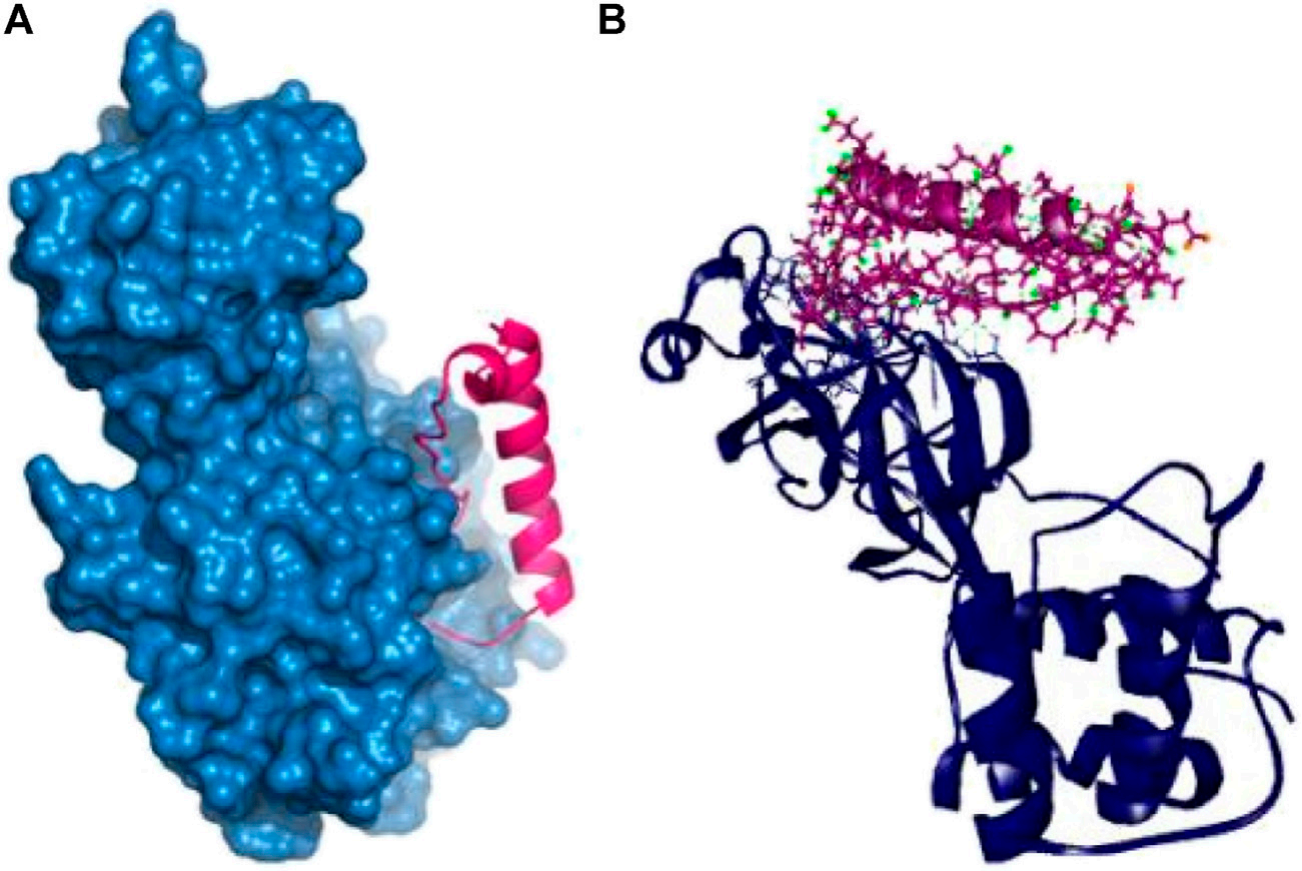
Nonbonded interaction of P74 and main protease enzyme **(A,B)**.

The P74 peptide and protein complex also generate ten hydrogen bonds at Glu166, Ser139, Phe140, Leu167, Ile135, Gln189, Ser139, Met165, Pro168, and Asp187 residues. This complex had one Pi-sulfur bond at Cys145 and one alkyl bond at Leu141. The P74 complex also had interaction in the catalytic residues at Cys145, Leu141, Ser139, Phe140, Leu167, and Gln189. These interactions at the active site of the protein may be responsible for the higher binding energy compared to the other peptides’ molecules (Table 2 and Figure 4).

### Molecular Dynamics Simulation

In the computational drug discovery process, the flexibility of protein plays a significant role in ligand binding. Thus, to predict the motion of the protein, many computational techniques are needed. Unfortunately, the required calculations for demonstrating chemical reactions between complex molecular processes and farcical quantum-mechanical motions are mostly very much perplexing to understand. To surpass these complexities and invigorate atomic motions, molecular dynamics (MD) simulations use facile presumptions that are a cornerstone of Newtonian physics (Liu et al., 2018). To identify cryptic binding sites and keep the proteins and ligands flexible and calculate the ligands’ binding energies and allow binding sites’ relaxation close to the ligand, the evaluation of (MD) is essential (Okimoto et al., 2009). The study of molecular dynamics simulation in the main protease of SARS-CoV-2 was being used to figure out how flexible all the docked complex (four peptide complexes), and this evaluation was lasted up to 200 ns. Assimilating these peptides’ complex properties of dynamics and motion is another application of dynamics simulation. Additionally, RMSD was seen to comprehend confirmational fixity. Besides the analysis of the docked complex solvent, the accessible surface area was observed. Further, gyration profiles radius was investigated to recognize the variation in protein mobility. MD simulation resulting in all the peptide complex conditions was firm and inflexible while binding with the SARS-CoV-2 main protease.

**FIGURE 5:**
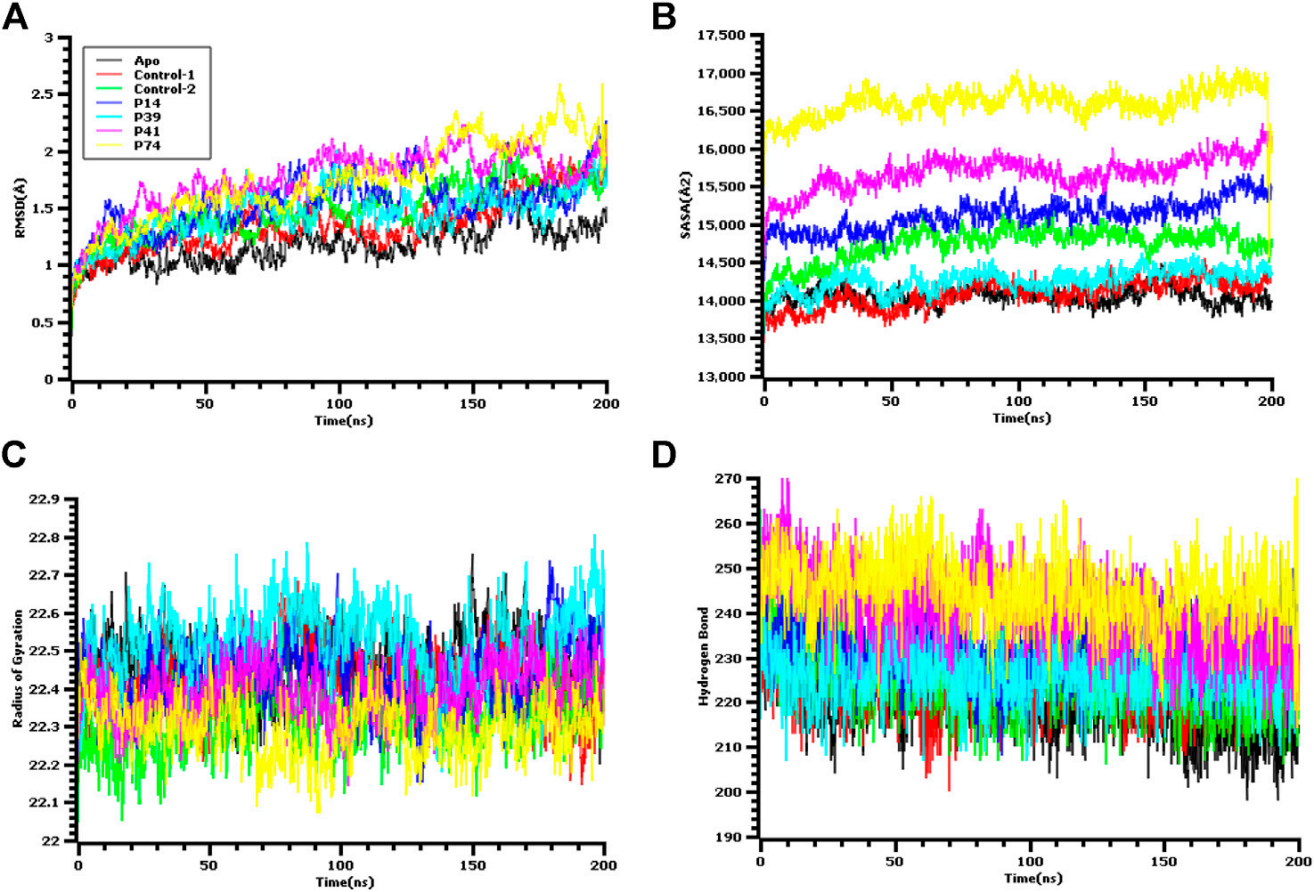
Molecular dynamics simulation study of the peptide and main protease enzyme. **(A)** Root mean square deviation of the C-alpha atom, **(B)** the solvent accessible surface area of the protein volume to understand the change in protein volume, **(C)** the radius of gyration, and **(D)** the hydrogen bond of the protein complex to evaluate their stability in simulation.

The ligand-free complex (apo), co-crystalized ligand (control-1), and peptide-bound main protease (control-2) were included in the dynamic simulation study to compare the motion and dynamics properties of the four peptide complexes. The root mean square deviation of the C-alpha atoms from the seven complexes was found in stable condition in Figure 5. The average RMSD descriptors from seven complexes: apo, control-1, control-2, P14, P39, P41, and P74 were 1.17, 1.35, 1.479, 1.53, 1.45, 1.75, and 1.74 Å, respectively (Figure 5A). The P41 and P74 peptide complexes were initially stable from 0 to 90 ns, and a higher RMSD trends were observed for both complexes. But large deviation was not seen for both complexes, but they were in the below range of 2.5 Å. The control and apo complexes along with P14 and P39 complexes exhibit lower RMSD trend across the whole simulation time. This lower RMSD trend among the all seven complexes indicated the conformational stability of the docked and control complexes. Therefore, upon peptide binding with the target protein of SARS-CoV-2, lower degree of conformational variation was observed as all of the complexes were found rigid in dynamic simulation (Nainu et al., 2020; Rakib et al., 2020a; Rakib et al., 2020b; Rakib et al., 2020c; Tallei et al., 2020; Harapan et al., 2021; Mahmud et al., 2021a).

The solvent accessible surface area of the docked complexes was also analyzed. These simulation descriptors correlate with the surface volume of the complexes where higher SASA profile indicates the expansion in the surface area. The P39 complex had similar SASA trend as the peptide size was comparatively lower than other peptides (Figure 5B). So, this complex had similar surface area like control and apo. Although higher SASA trend were observed for rest of the peptides, but deviations in SASA value were absent for these complexes, thus these peptide complex had strict surface area across the simulation complex. The average SASA profile for the six complexes: apo, control-1, control-2, P14, P39, P41, and P41 were as 14048.28, 14075.69, 14738, 15115.54, 14261.35, 15679.8, and 16117.22 Å^2^, respectively.

The radius of gyration profile indicated the change in the protein mobility across the simulation time. The P74 peptide had lower Rg value than the control and other peptide molecules. After 60 ns, this complex lowered its Rg profile more and higher again after 100 ns (Figure 5C). The lower radius of gyration profile of this complex indicates the compacted nature of the protein. On the other hand, the P39 complex, apo, and control complex had higher deviation which correlates with lability of the complex and also possibility of the folding or unfolding nature of the complexes. Moreover, the hydrogen bond of the systems can determine the stable nature of the complexes. The P74 and P41 peptides had more hydrogen bonds than the control and apo complexes. The other two peptides, P14 and P39, had similar hydrogen pattern with the ligand-free complexes and control (Figure 5D). Although number of hydrogen bonds were different for all seven complexes, but they did not change the stable nature as lower flexibility were observed for the peptide complexes.

**FIGURE 6:**
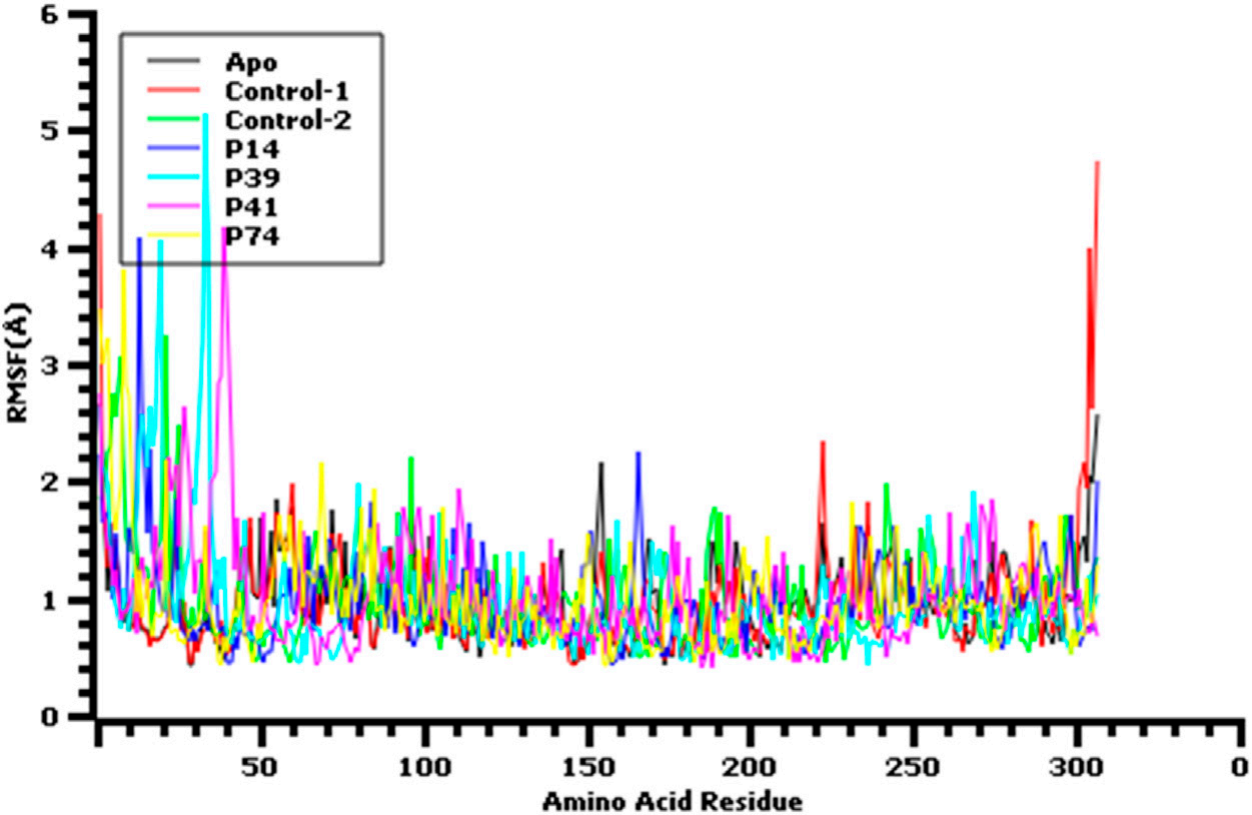
Root mean square fluctuation of the amino acid residue of the protein complex to understand their fluctuations across the residues.

The root mean square fluctuation or RMSF of the amino acid residues from main protease enzyme was also analyzed (Figure 6). The complex had lower RMSF value except Ser1 (helix), Ala7 (helix), Val13 (helix), Gln19 (helix), Leu32 (helix), Asp33 (helix), Pro39 (helix), Arg40 (helix), Pro96 (beta turn), Tyr154 (beta turn) Glu166, Arg222, (beta turn), Gly302, (beta turn), Val303, (beta turn), Thr304, (beta turn), Phe305, (beta turn), and Gln306 (beta turn) residues. The last amino acid segment from beta turn domain and first segment from helical region of this protein exhibit more flexibility. Overall, all other complex had lower RMSF profile than the other complex by denoting more rigidity and structural stability of the amino acid residues from main protease.

### SAR Analysis

**FIGURE 7:**
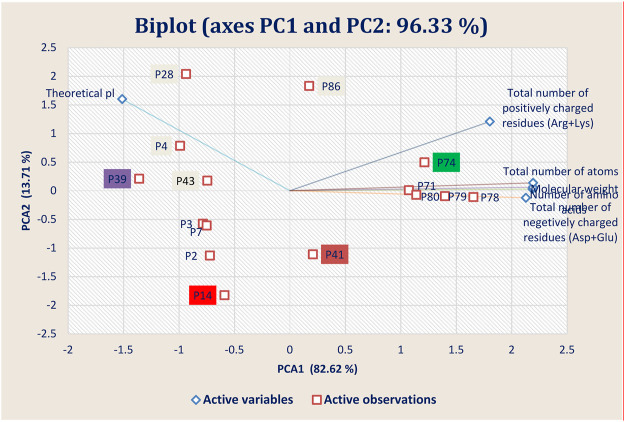
Principal component analysis (PCA) showing the Biplot of top 15 high-binding affinity peptides clustered based on four peptide properties.

The physiochemical properties of the best 15 peptides molecules were tabulated in Supplementary Table S2. The structural variation among the best 15 peptide molecules was assessed through clustering behavior. Figure 7 depicted that the best four peptides P14, P39, P41, and P74 and their clustering pattern were replicated the energy score plot of their complexes with main protease from SARS-CoV-2 but that P14 is close to P41 (Figure 7). Besides theoretical pl, total number of negatively charged residues (Asp + Glu) and total number of atoms and molecular weight residues play a significant role in the clustering pattern of the test peptides as negatively charged and total number of atoms residues are heavily loaded onto PC1 and the theoretical pl residue onto PC2, which altogether explains 96.33% structural variance.

Theoretical pl and total number of positively charged residues samples are clustered in the first and second quadrants, whereas the negatively charged residues samples were grouped in the fourth quadrants of the observation and biplots. This grouping accounted for 96.33% of the total variation present in the data set. Based on the sample ordination in principal component (PC1), total number of positively charged residues samples are located at positive values or close to zero compared to the theoretical pl samples, indicating a difference in the abundances of the compounds generated in both types of peptides.

## Conclusion

Modeling and designing antiviral peptides by targeting viral protein from SARS-CoV-2 can be promising, unique techniques to tackle SARS-CoV-2 treatment systems. The comparative modeling of screened peptide molecules allows us to evaluate the best peptide molecules’ binding energy. The combinatorial docking approaches were further assessed to find the vital active points of interactions where most of the binding residues were found in the binding sites. This docking approach had favorable results in a dynamics simulation study where every peptide was in a rigid state, and flexible nature was not found. Furthermore, these experiments, along with future *in vivo* and *in vitro* experiments, can lead to the rational and rigorous development of peptide-based inhibitors by targeting the viral protein of SARS-CoV-2.

## Data Availability

The original contributions presented in the study are included in the article/Supplementary Material, further inquiries can be directed to the corresponding authors.
