# Supplementary material for: Prospective Role of Peptide-Based Antiviral Therapy Against the Main Protease of SARS-CoV-2
Source: Front Mol Biosci. 2021 May 10;8:628585. doi: 10.3389/fmolb.2021.628585 (PMC8142691; doi:10.3389/fmolb.2021.628585)
Supplement: Supplementary file 1 [file datasheet1.docx]

Supplementary Material

# Supplementary Figures and Tables

## Supplementary Figures

**
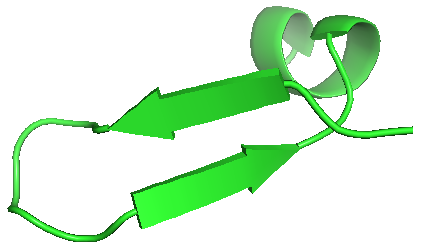
**
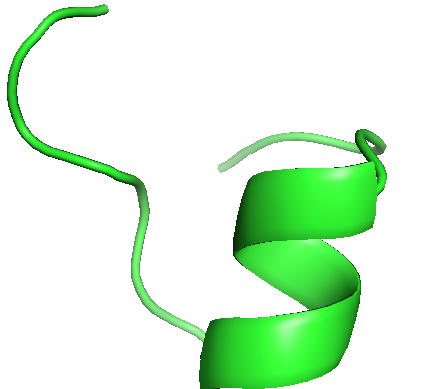


**P1 P2**

**
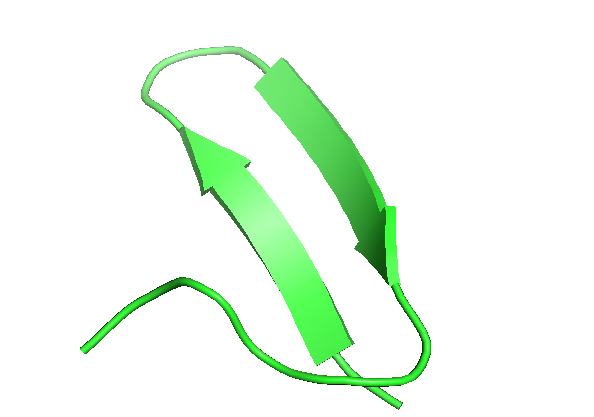

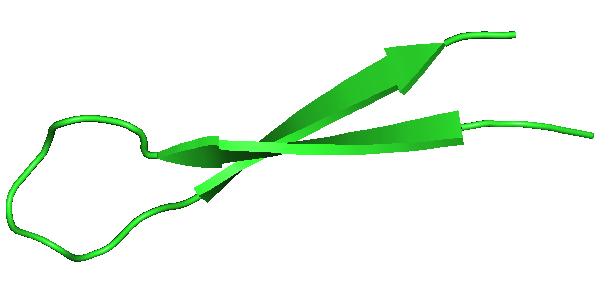
**

**P3 P4**

**
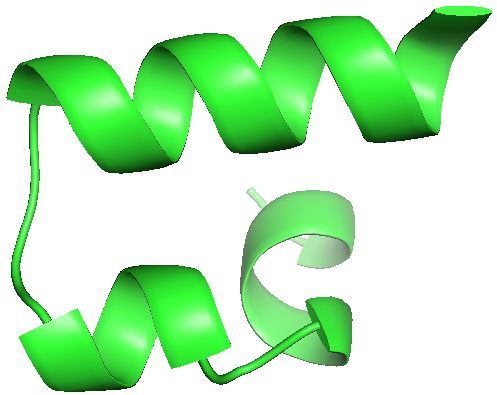

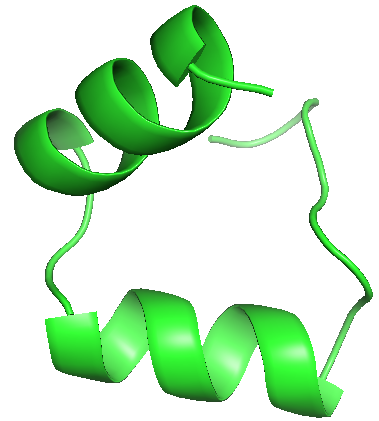
**

**P5 P6**

**
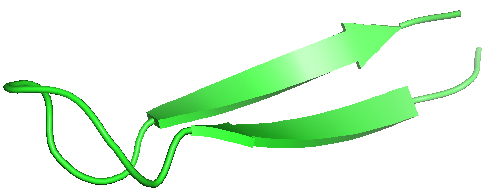

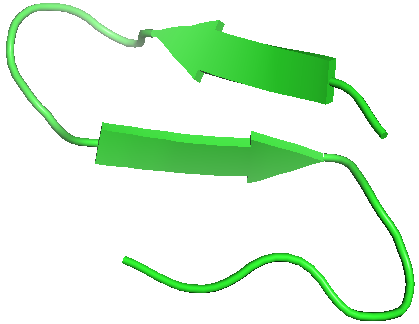
**

**P7 P8**

**
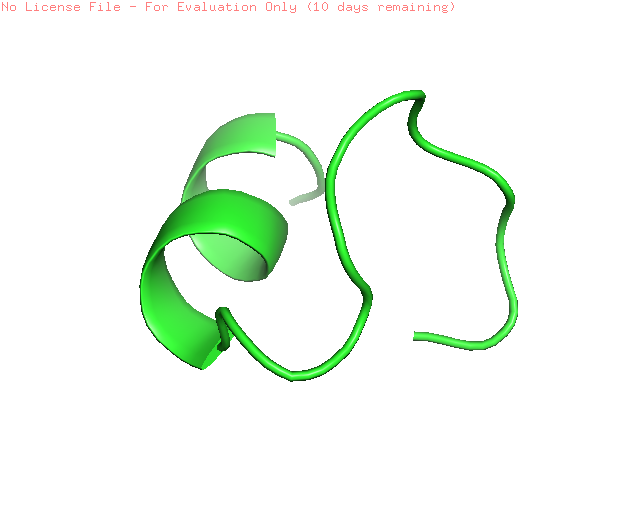

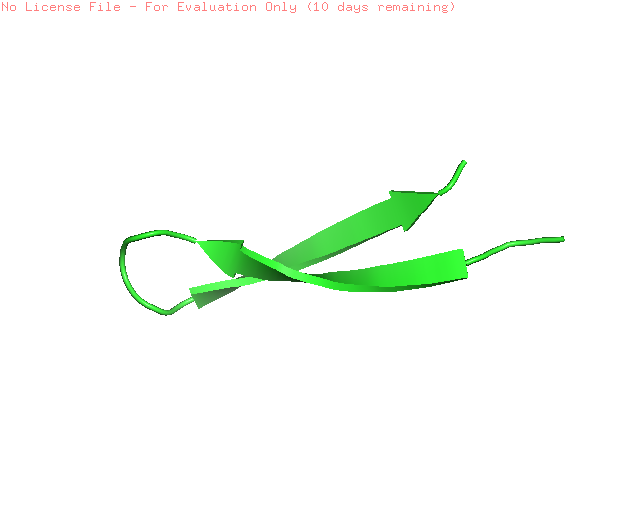
 P9 P10**

**
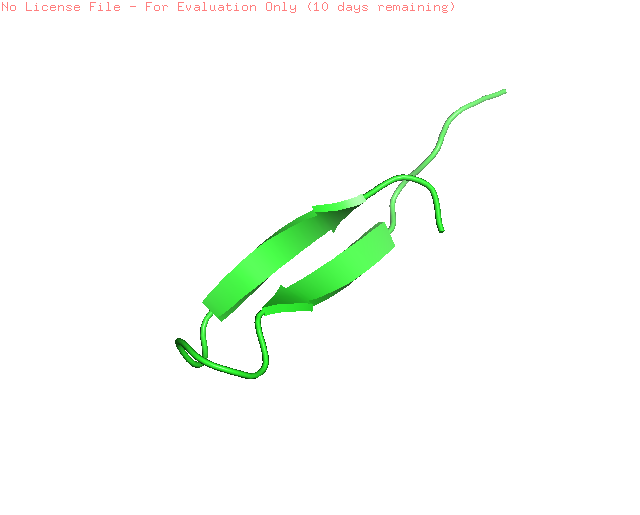

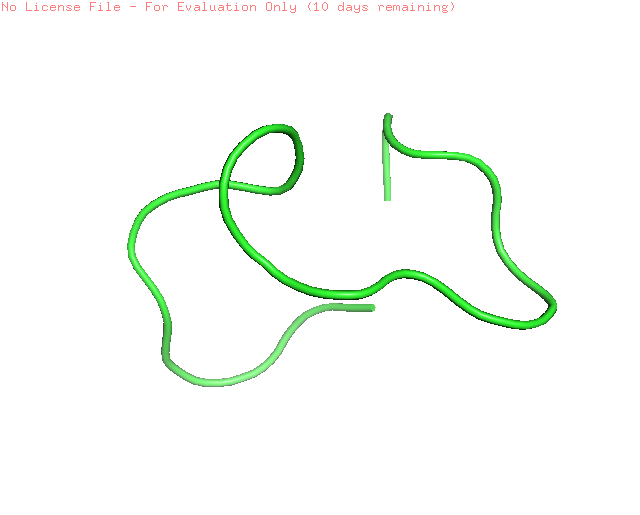
**

**P11 P12**

**
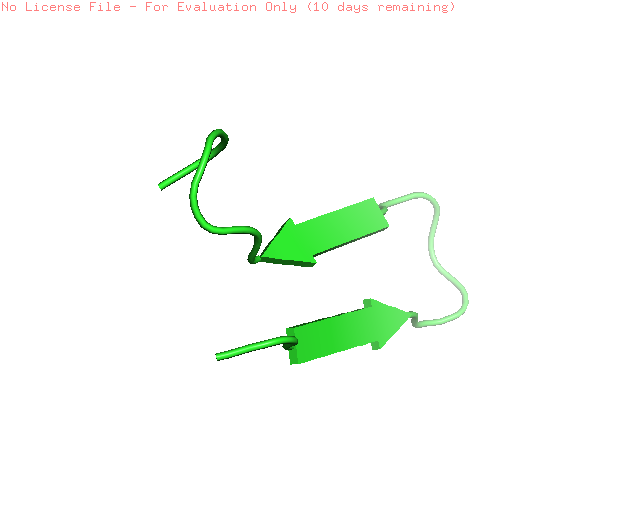

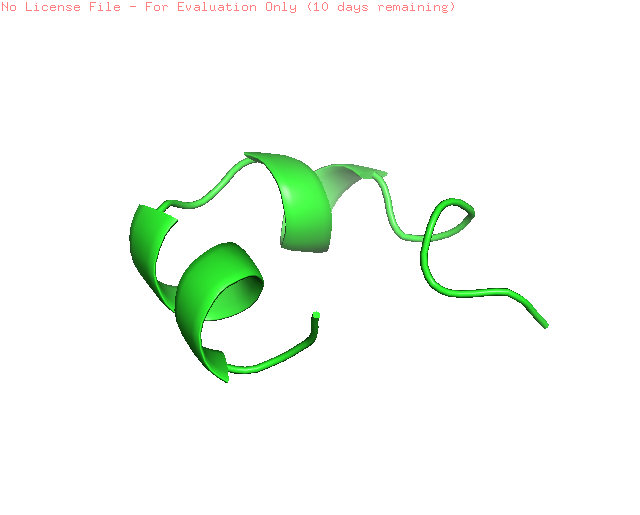
**

**P13 P14**

**
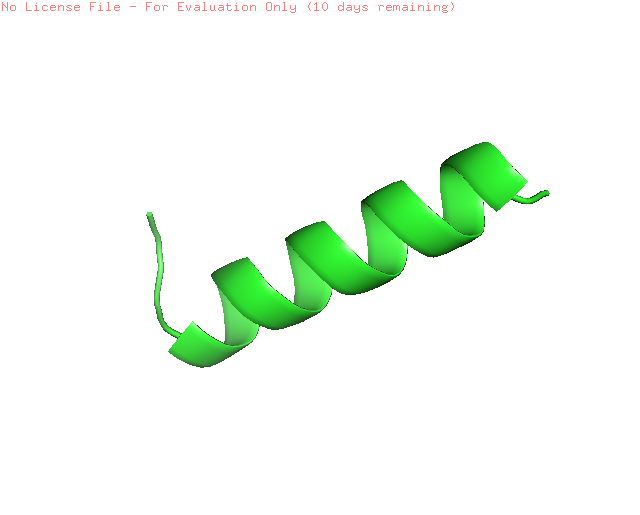

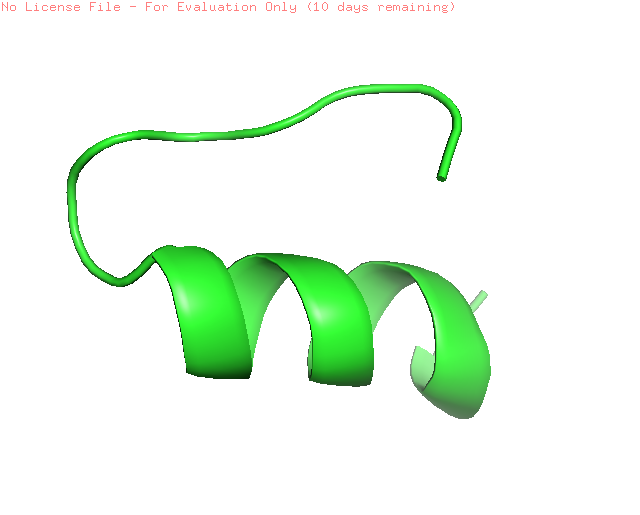
**

**P15 P16**

**
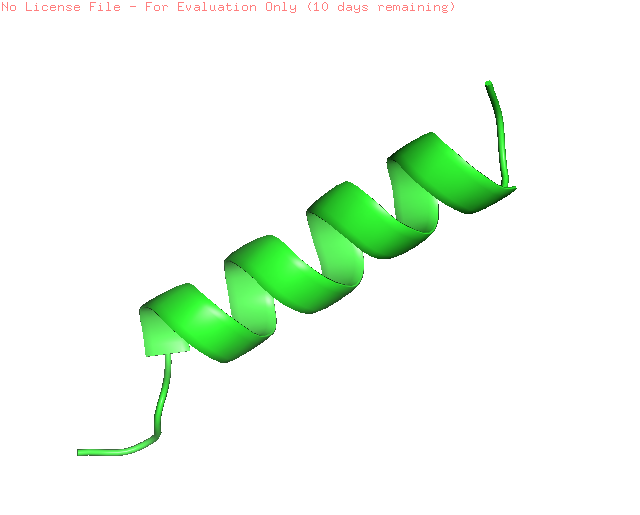

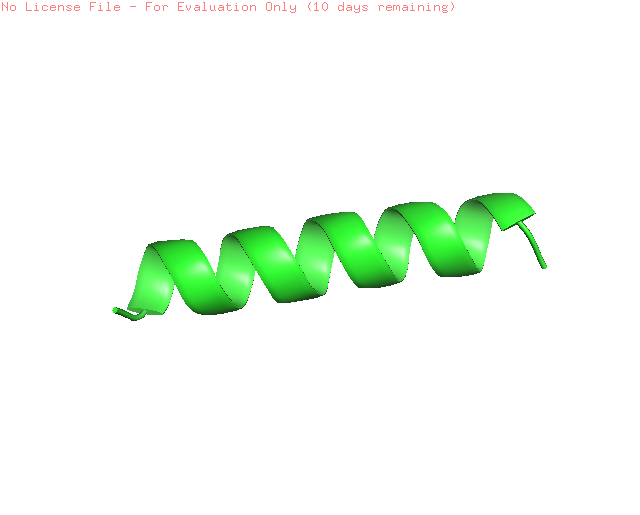
**

**P17 P18**

**
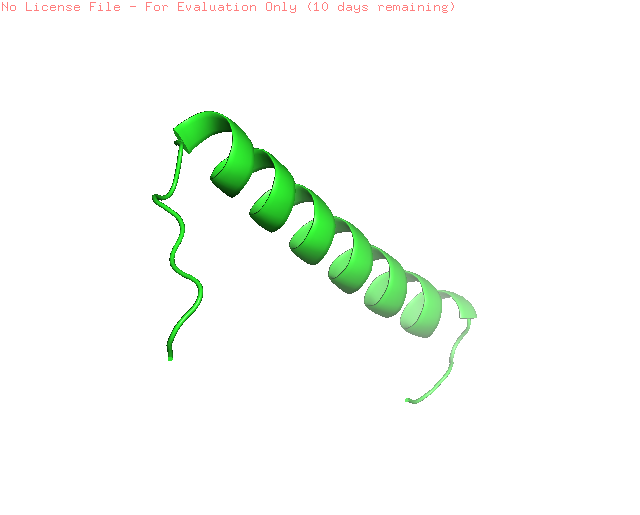

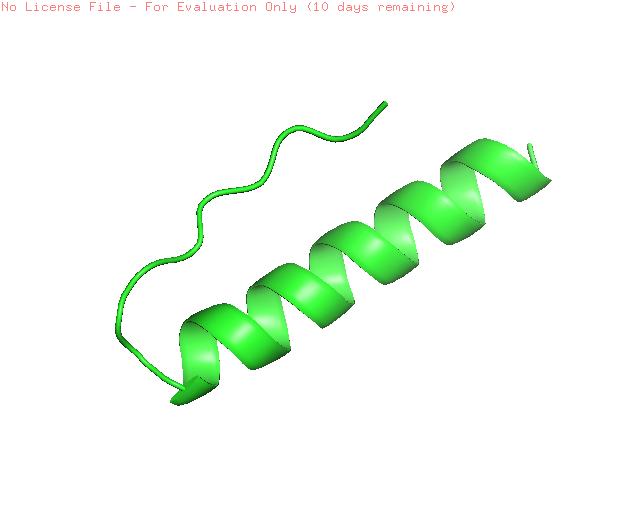
**

**P19 P20**

**
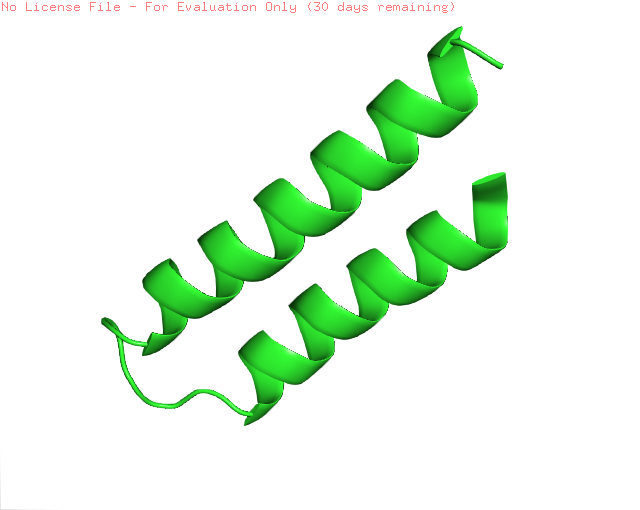
**
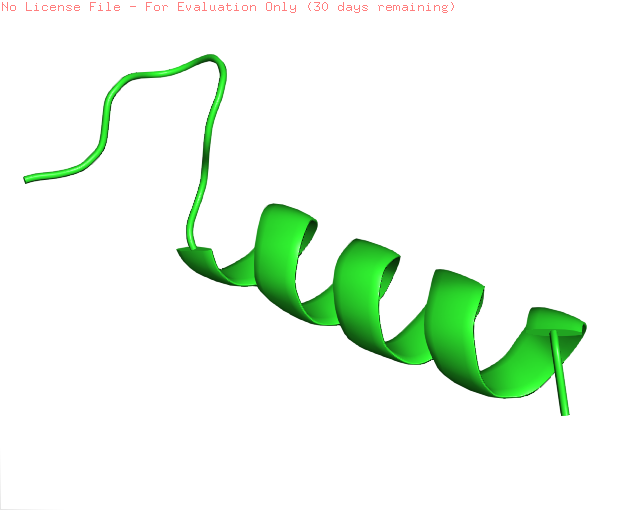


**P21 P22**

**
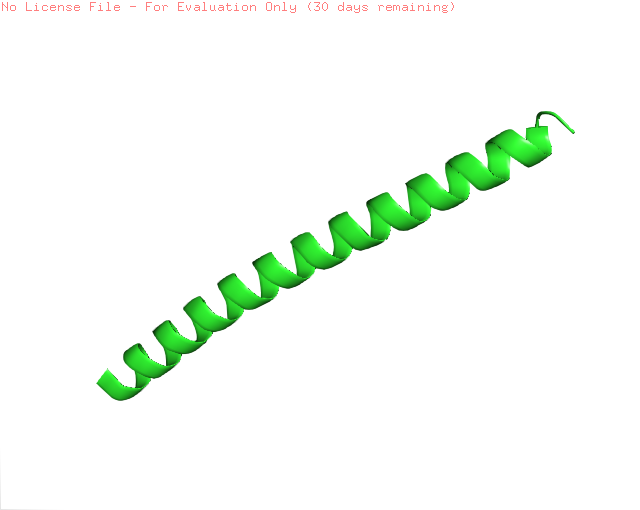
**

**
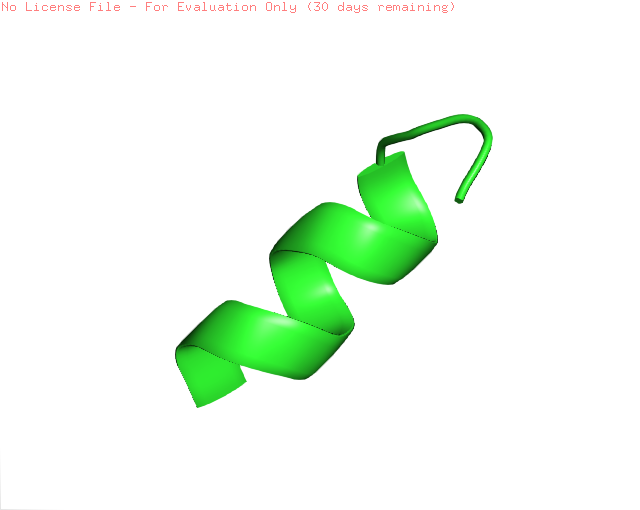
**

**P23 P24**

**
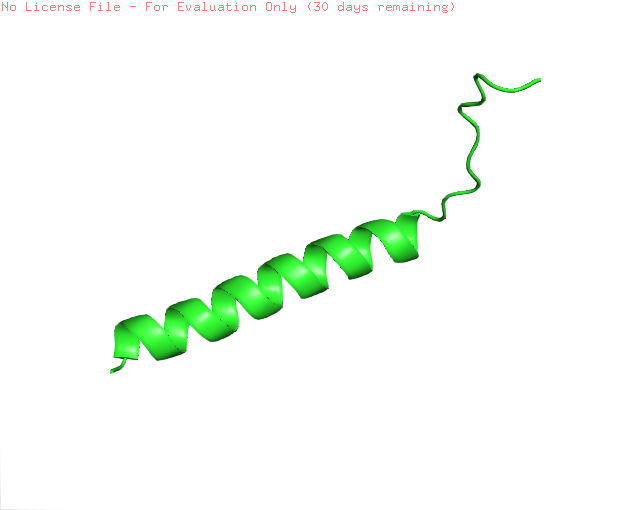
**

**
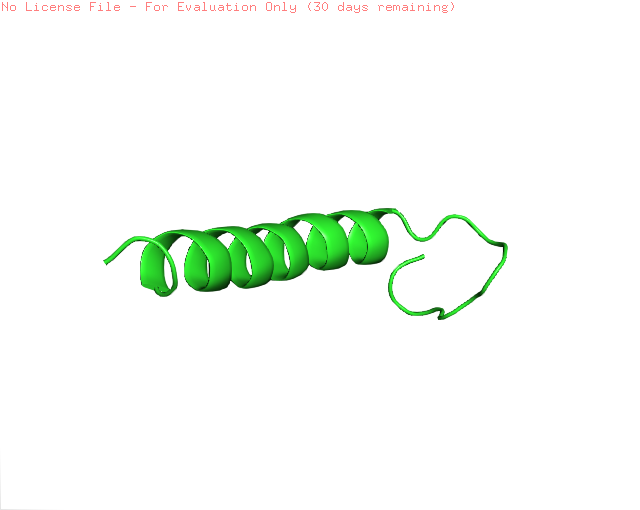
**

**P25 P26**

**
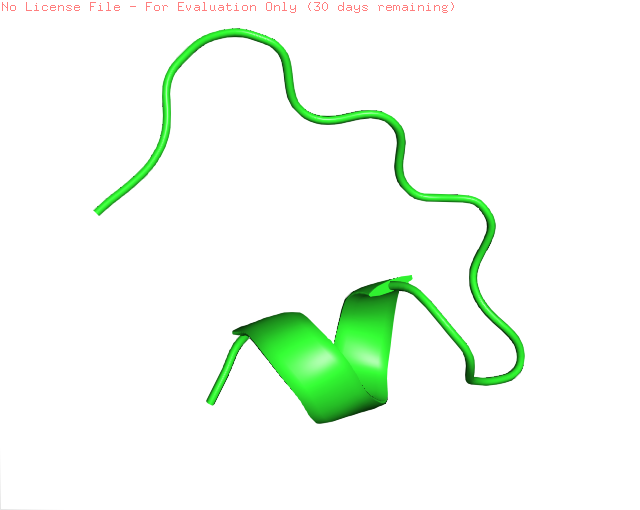

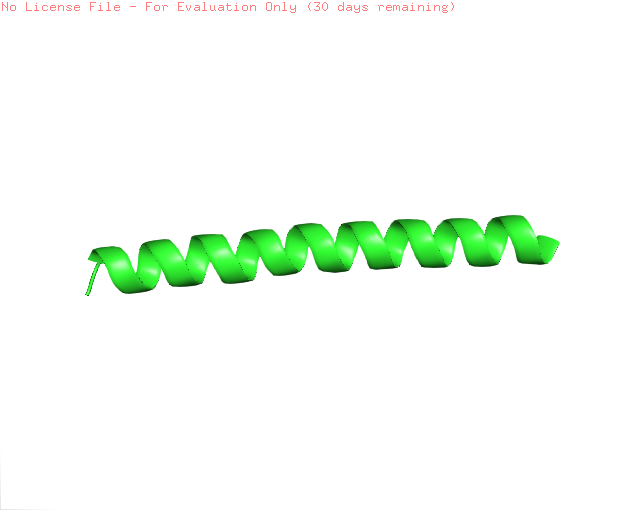
**

**P27 P28**

**
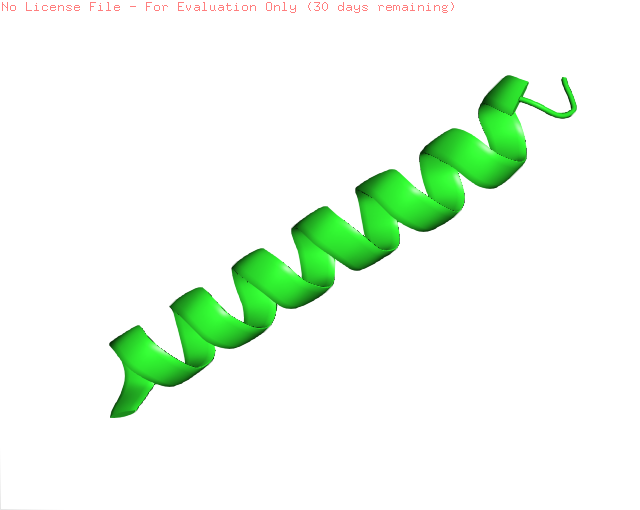

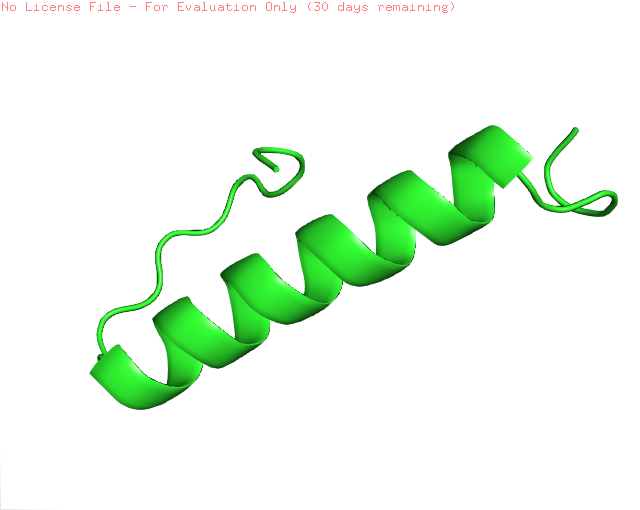
**

**P29 P30**

**
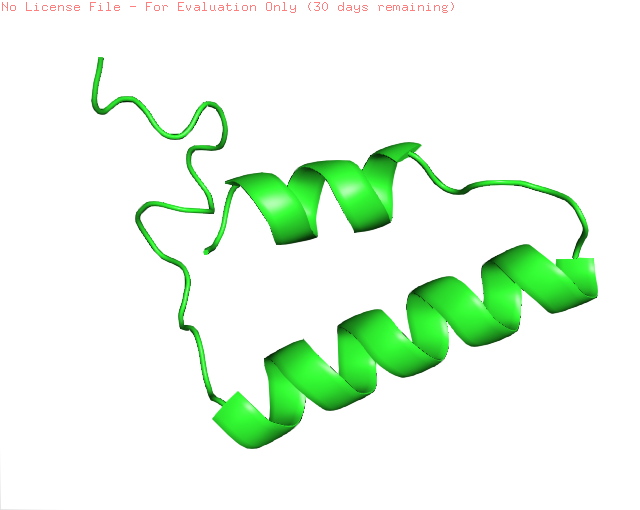

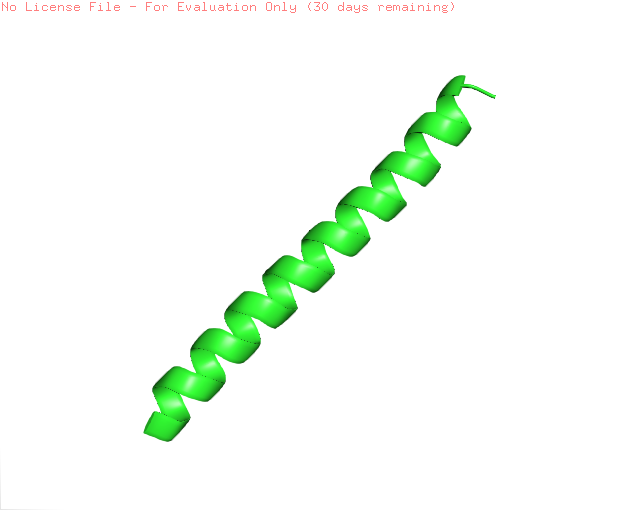
**

**P31 P32**

**
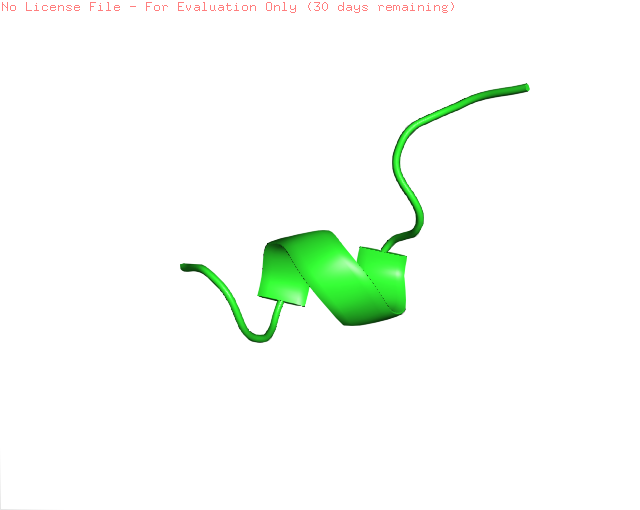

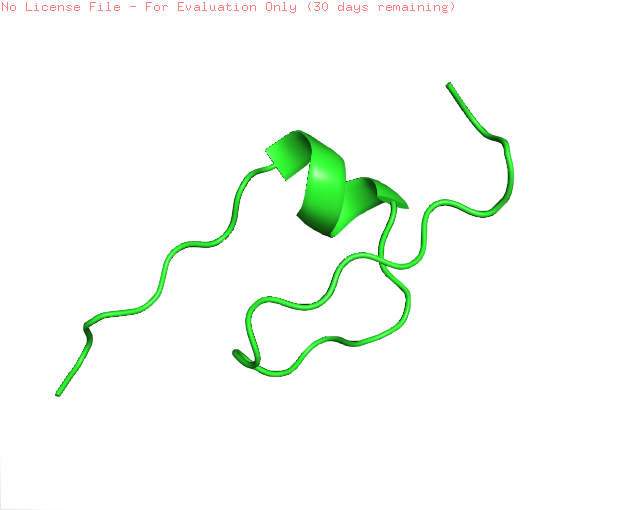
 P33 P34**

**
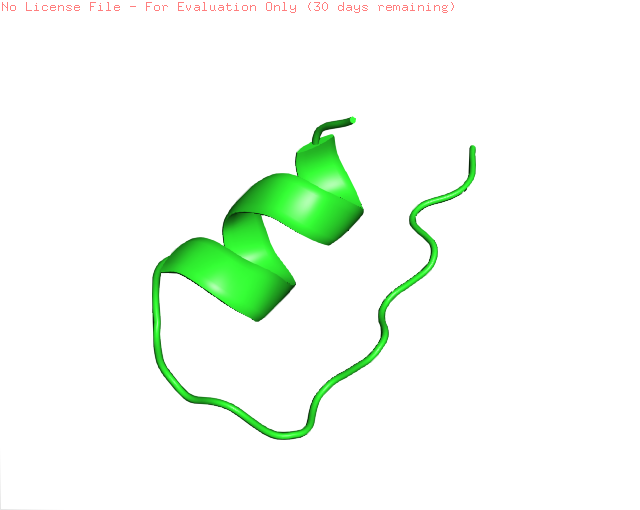

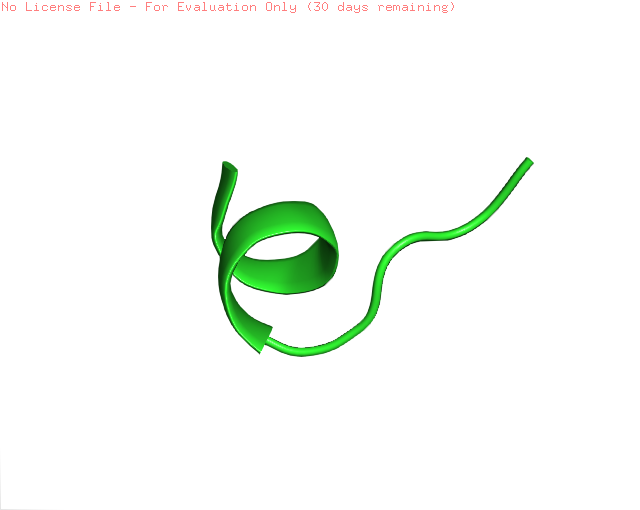
**

**P35 P36**

**
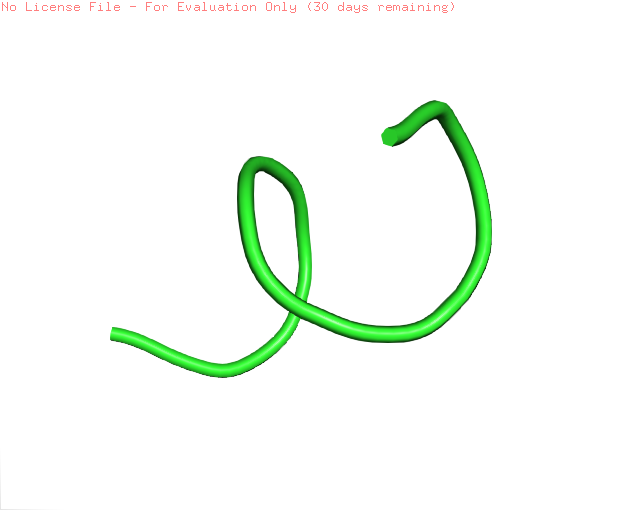

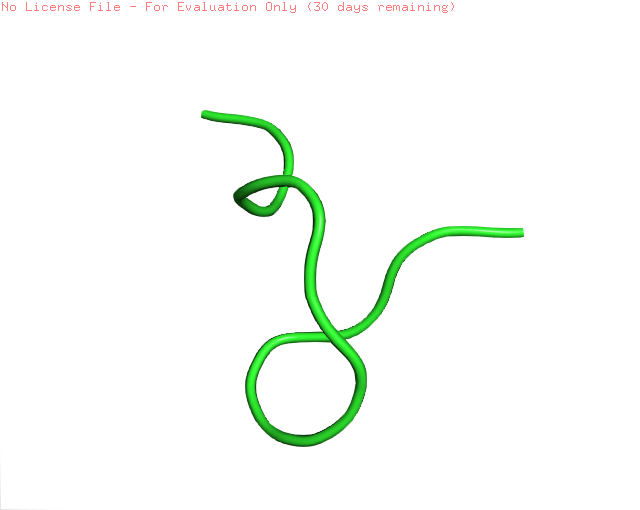
 P37 P38**

**
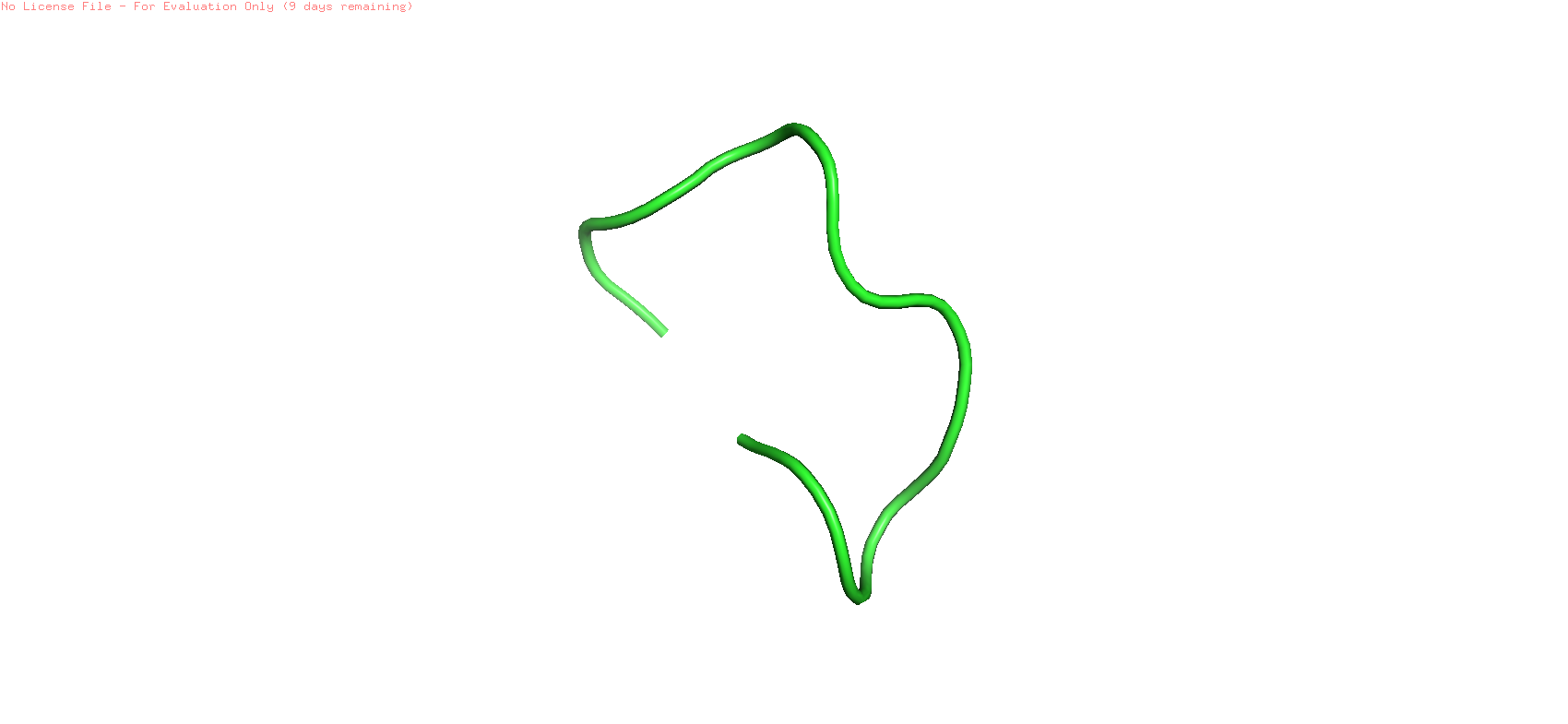

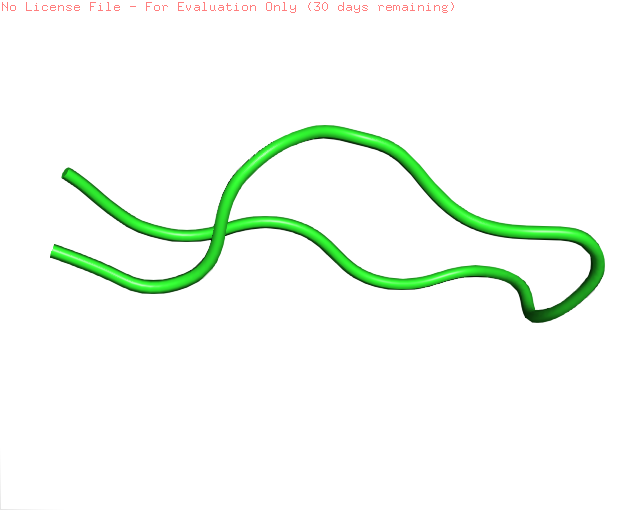
**

**P39 P40**

**
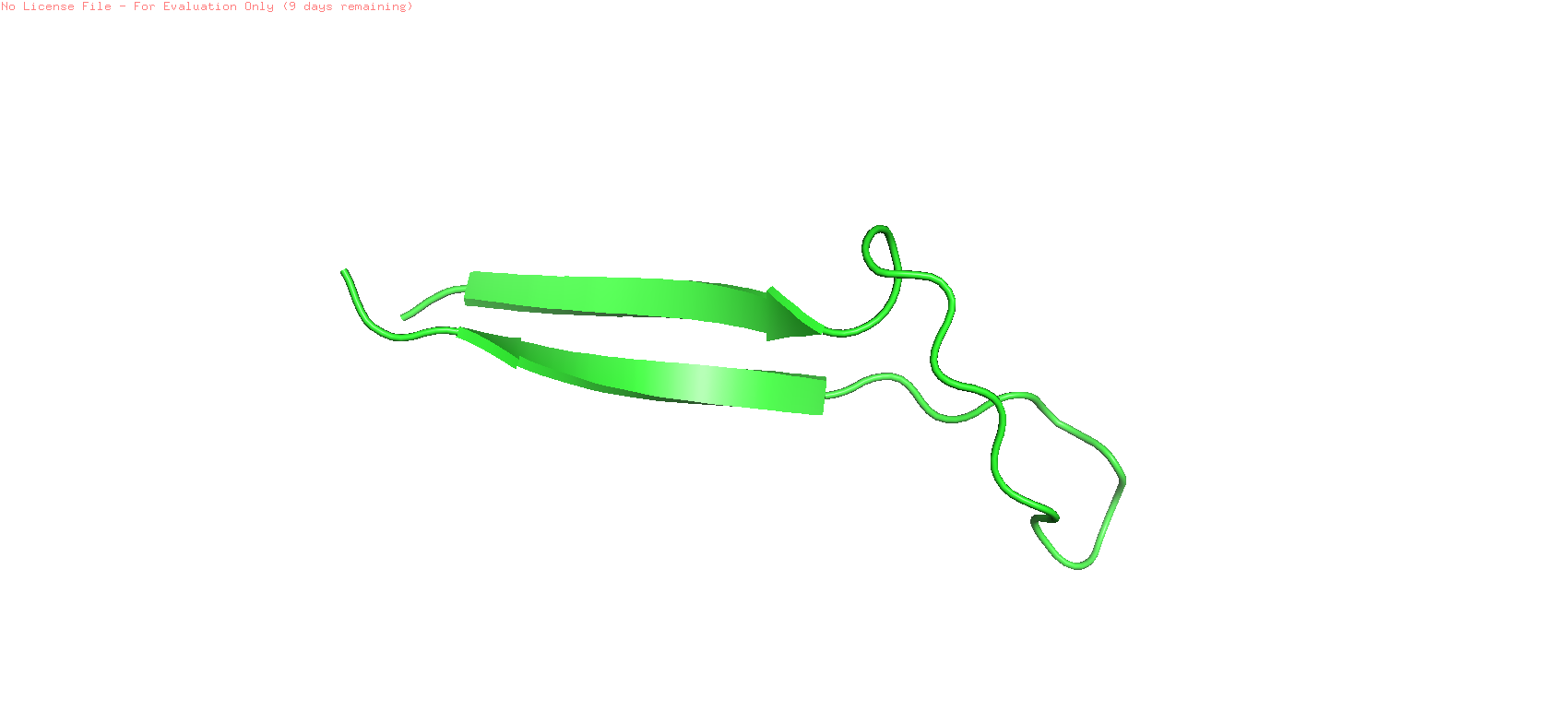

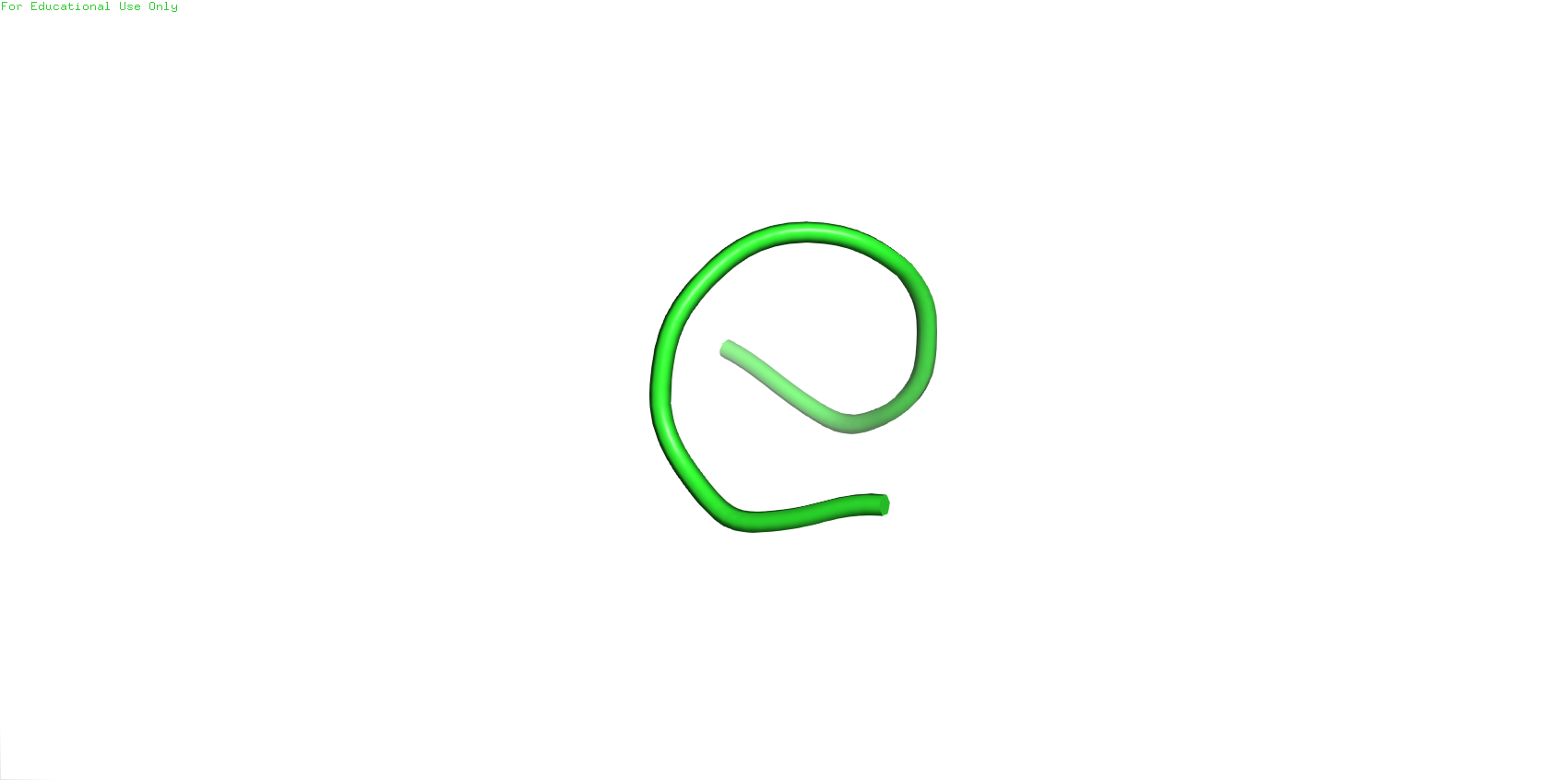
 P41 P42**

**
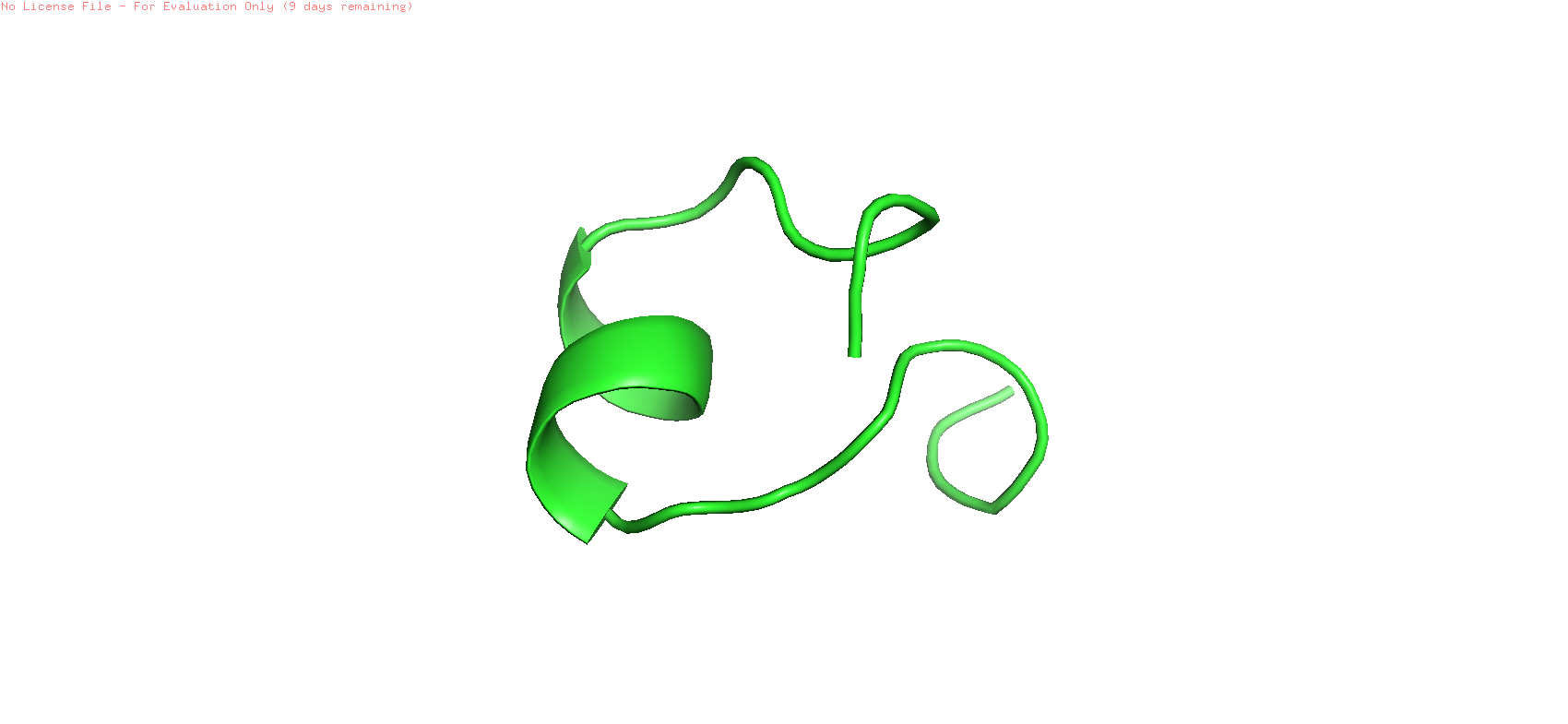

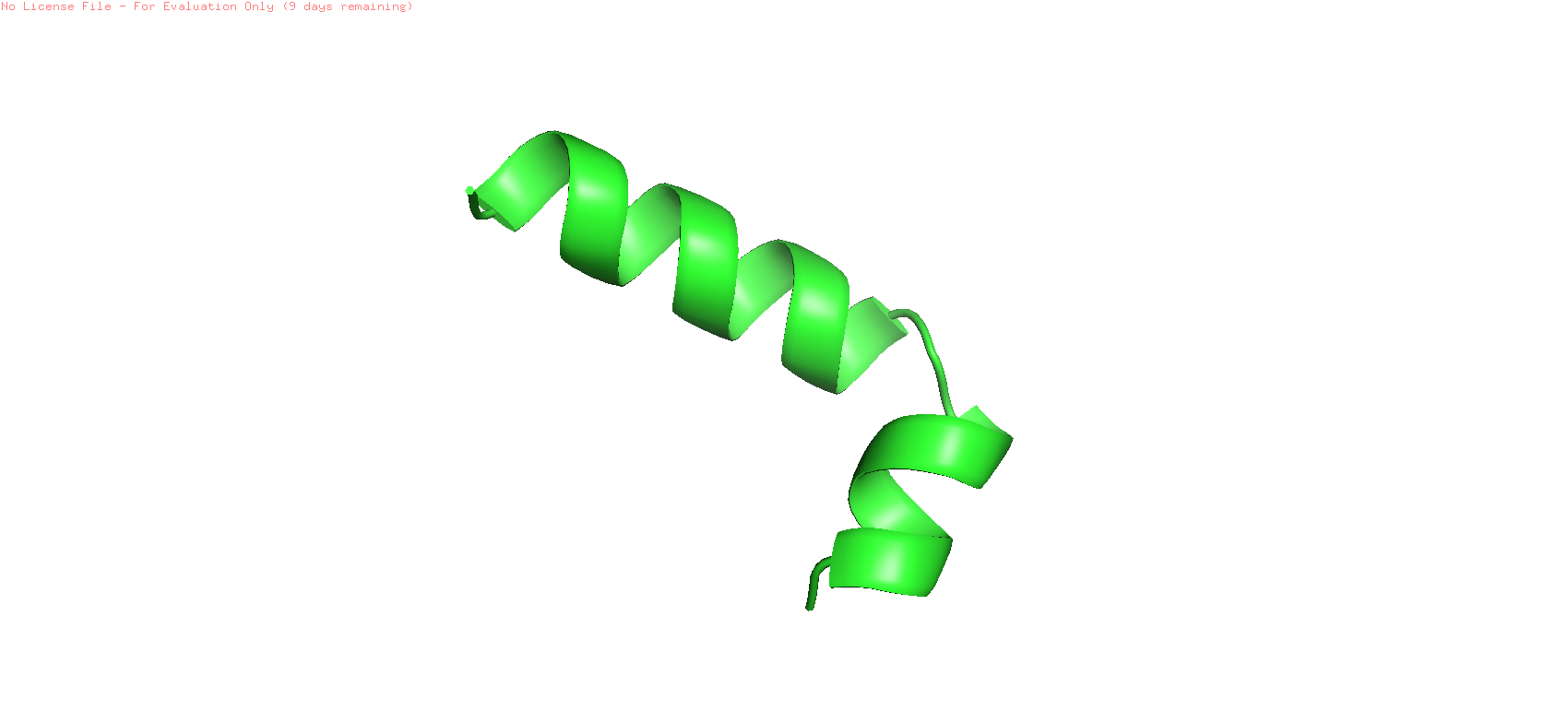
**

**P43 P44**

**
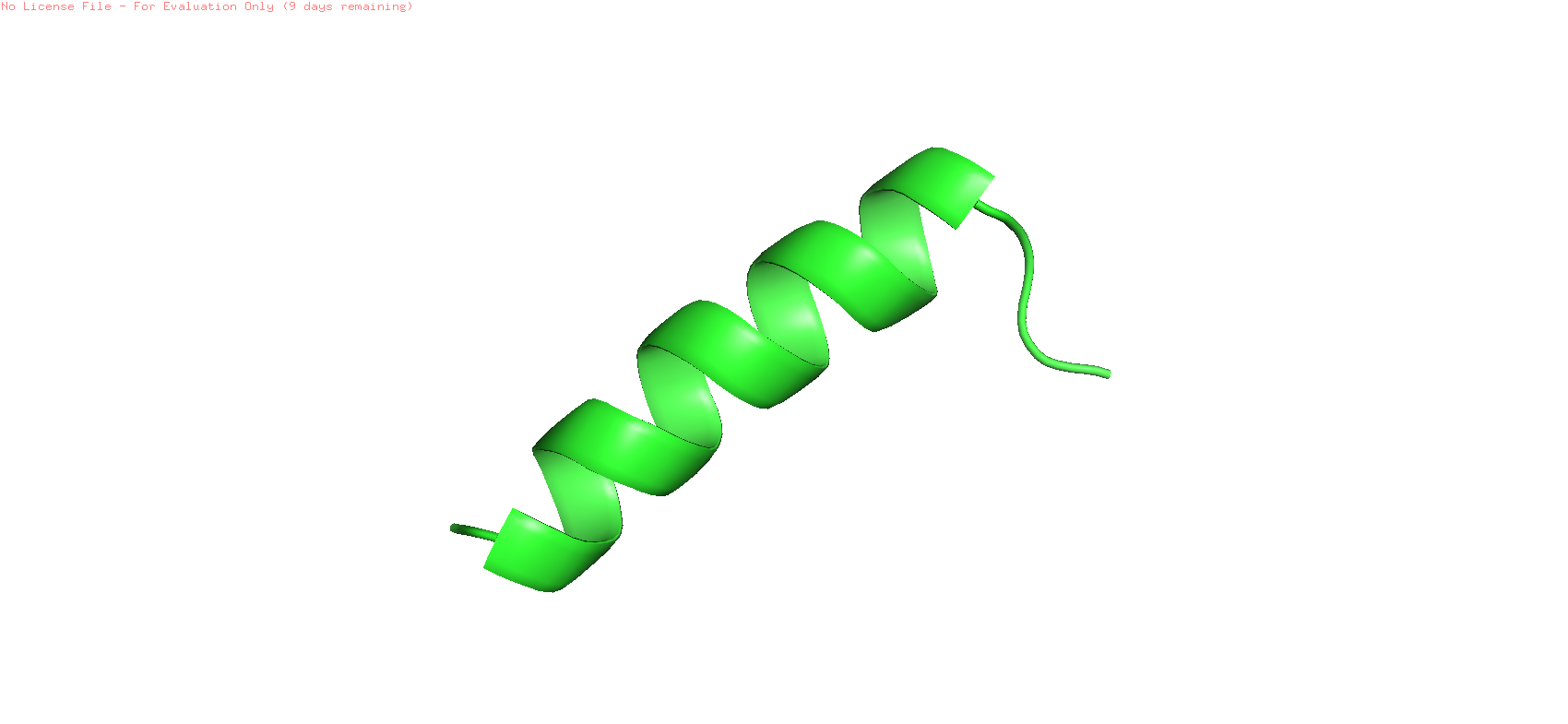

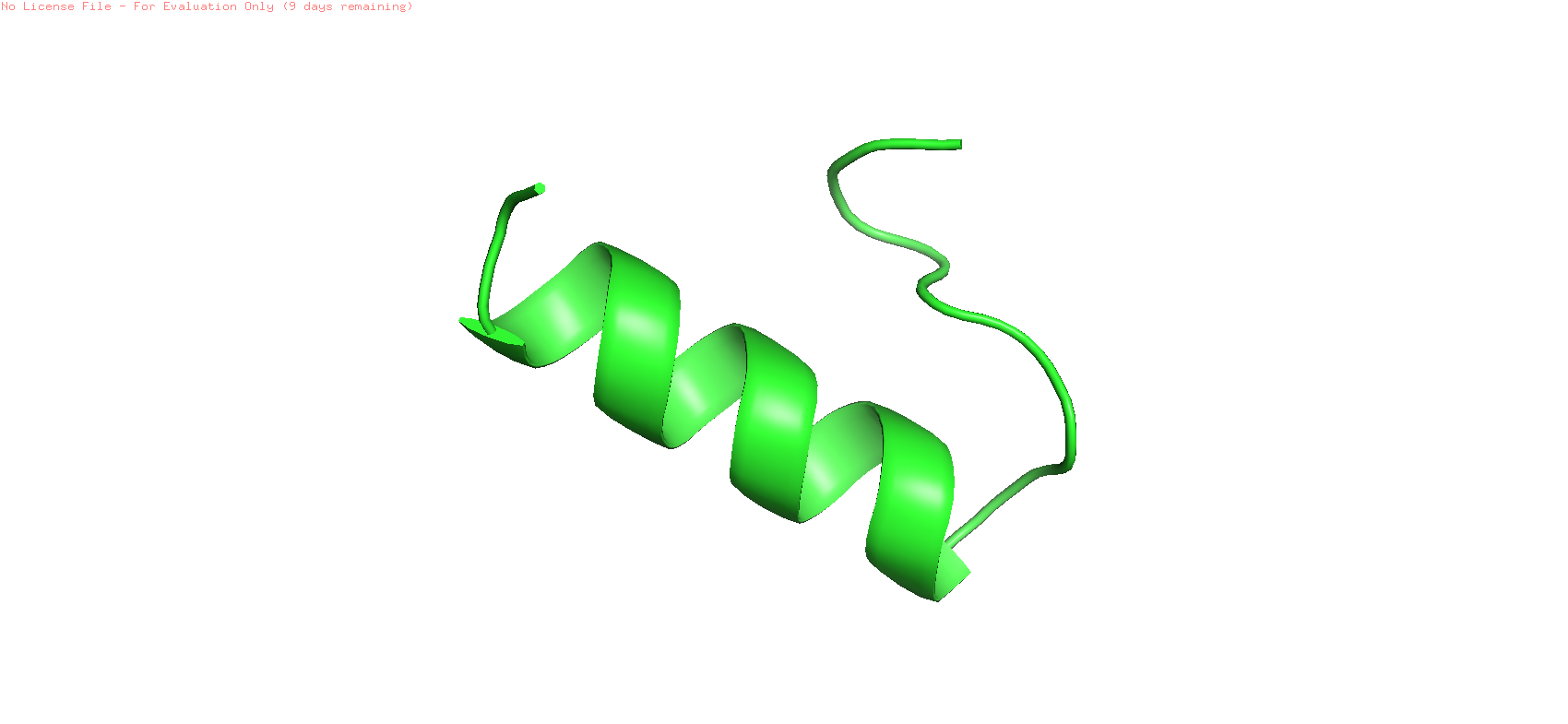
 P45 P46**

**
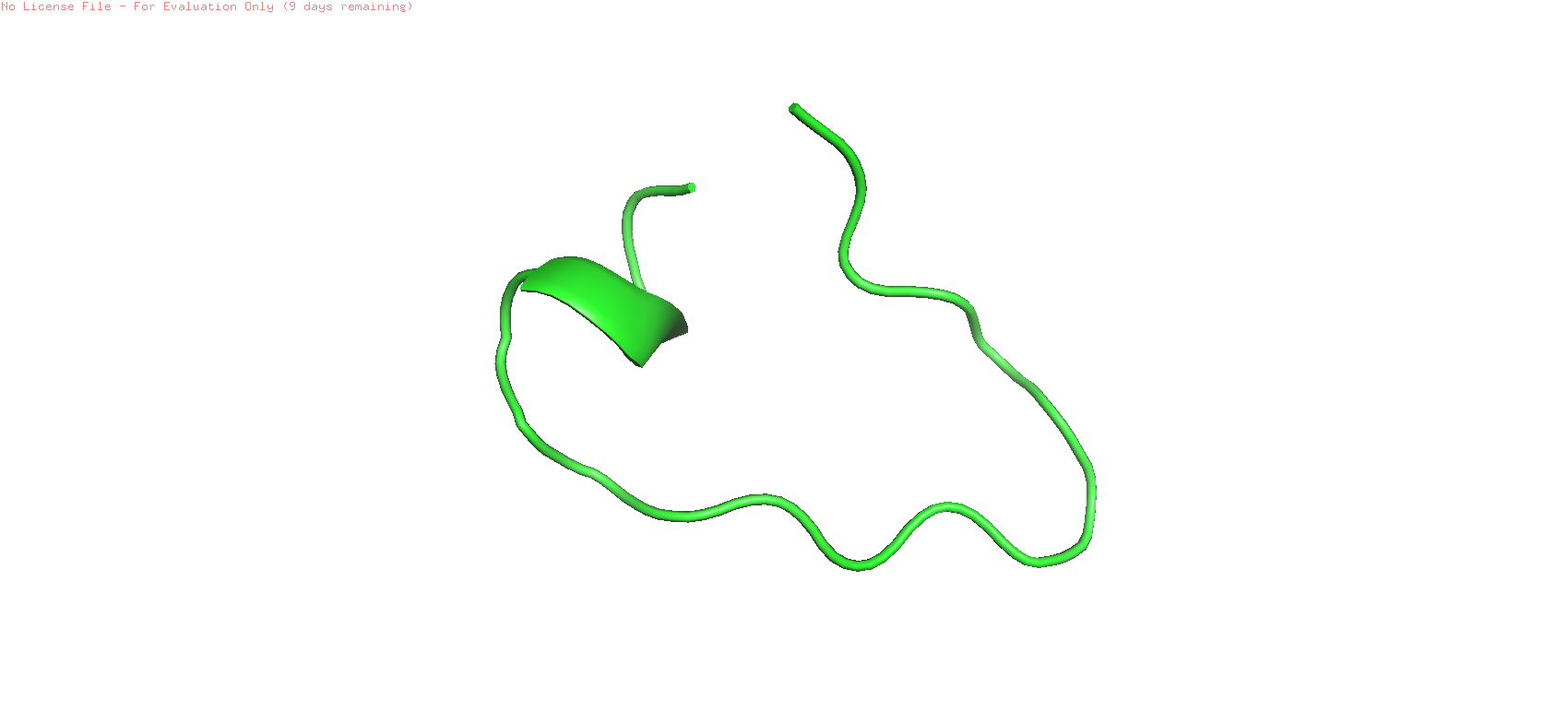

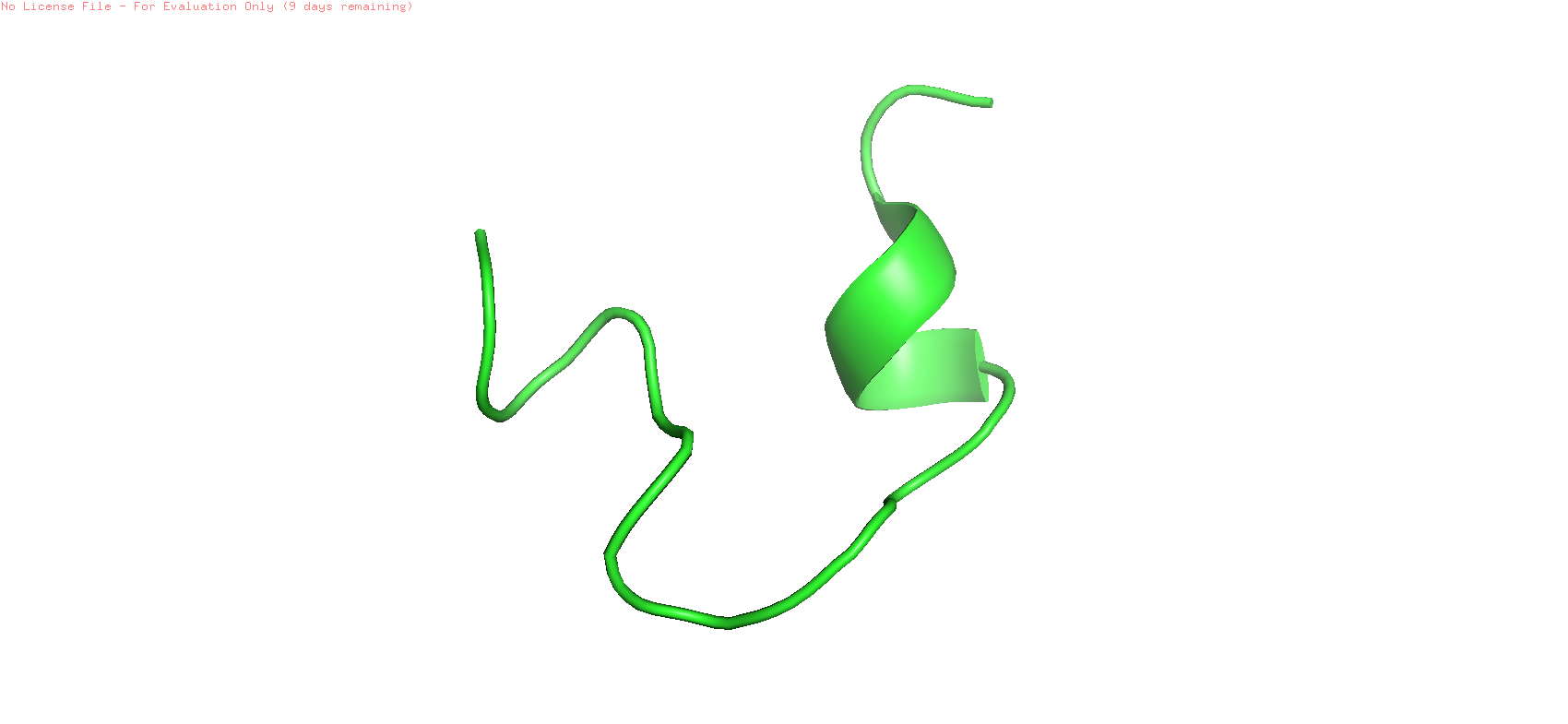
**

**P47 P48**

**
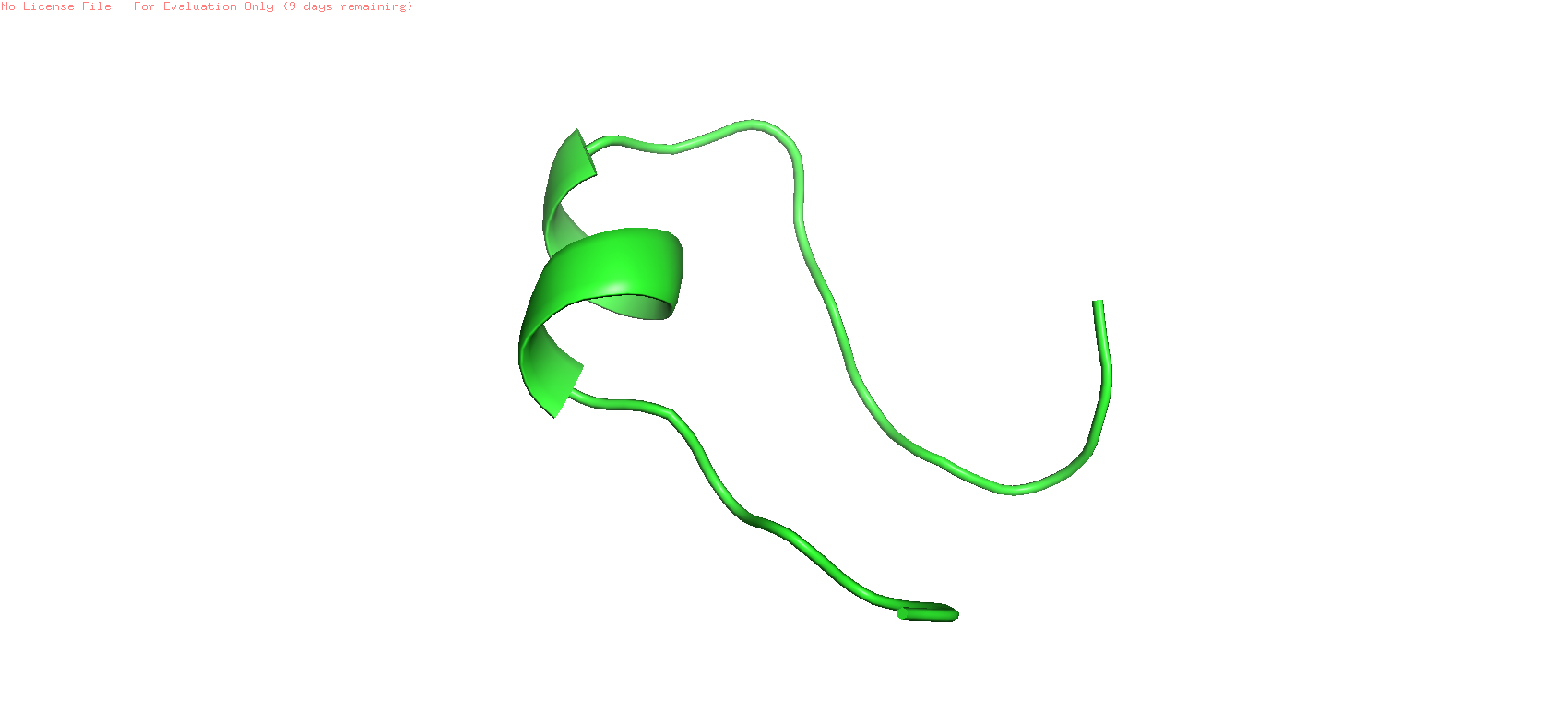

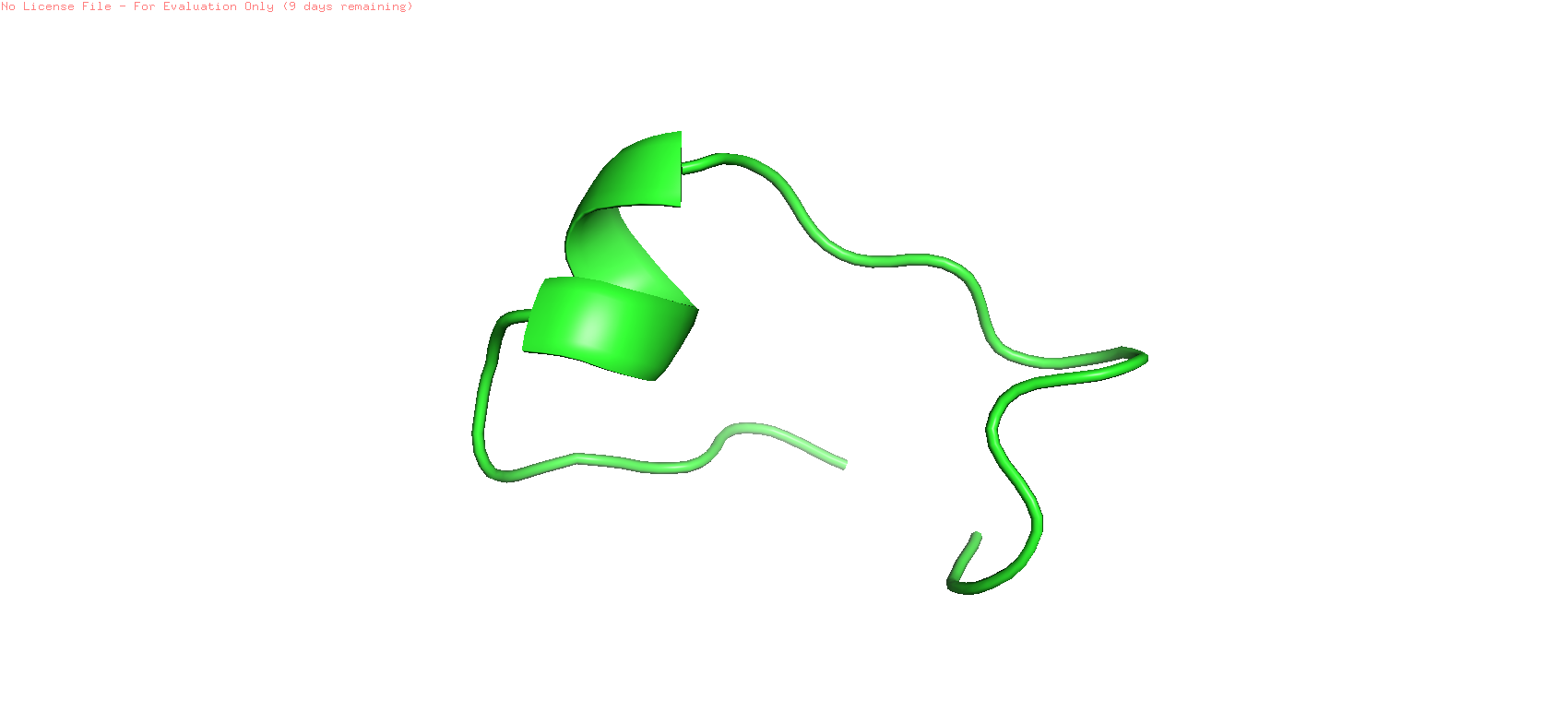
**

**P49 P50**

**
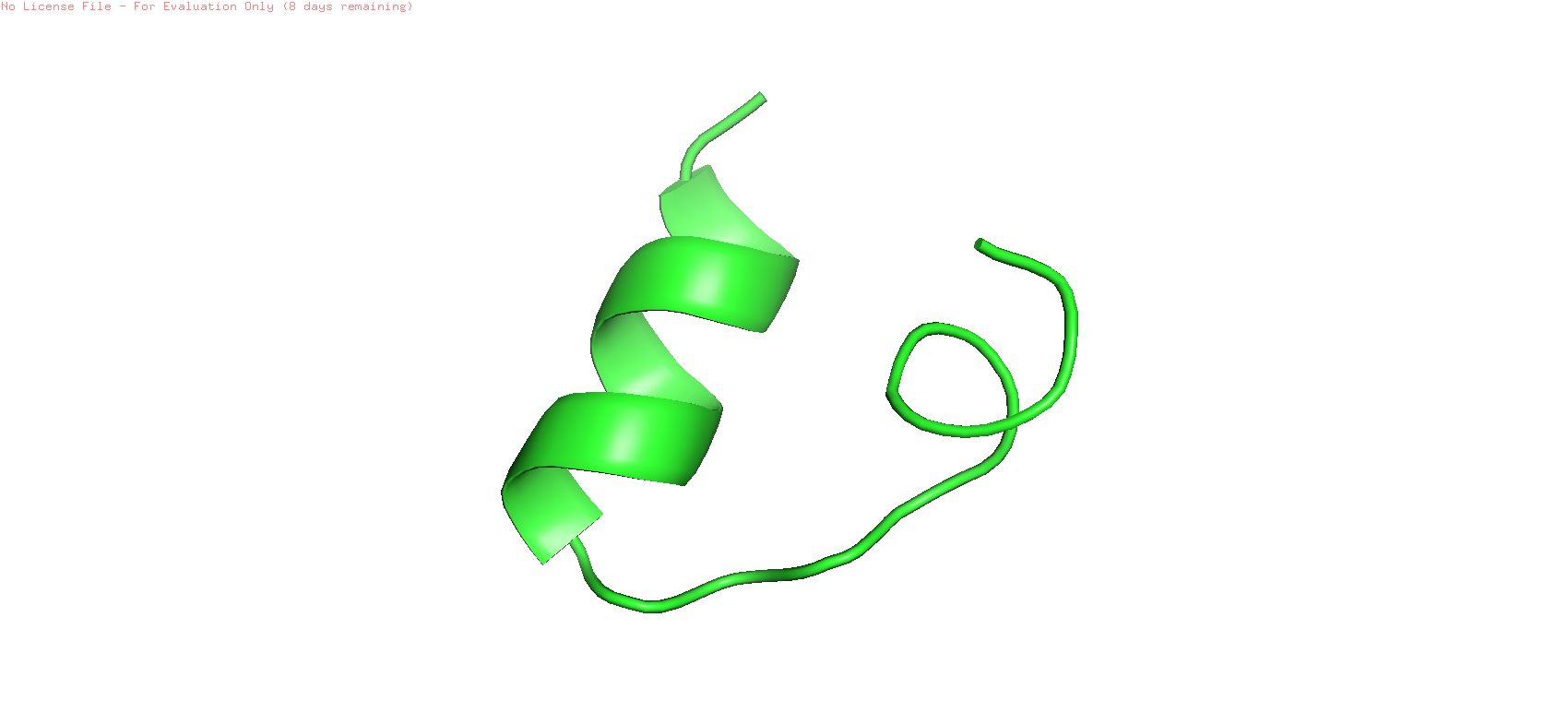

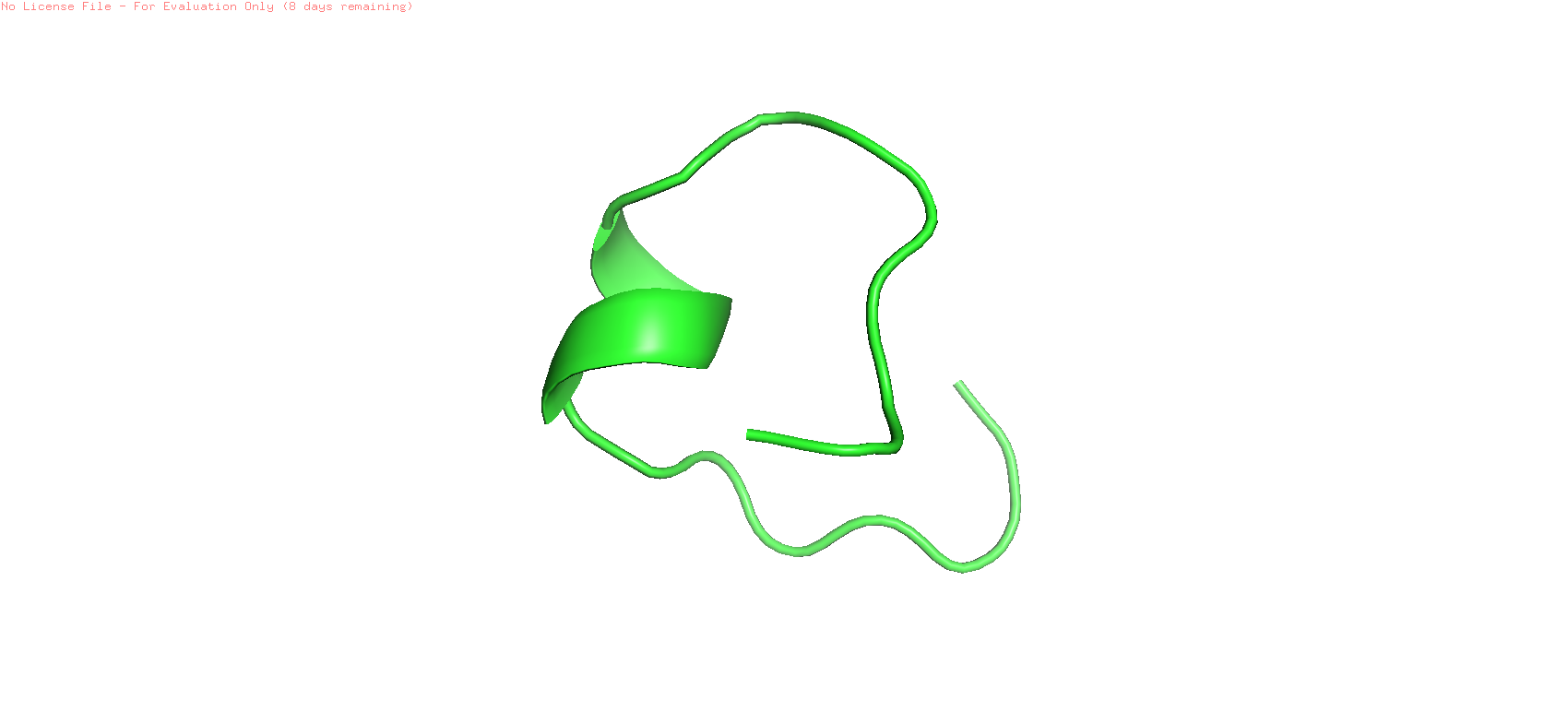
**

**P51 P52**

**
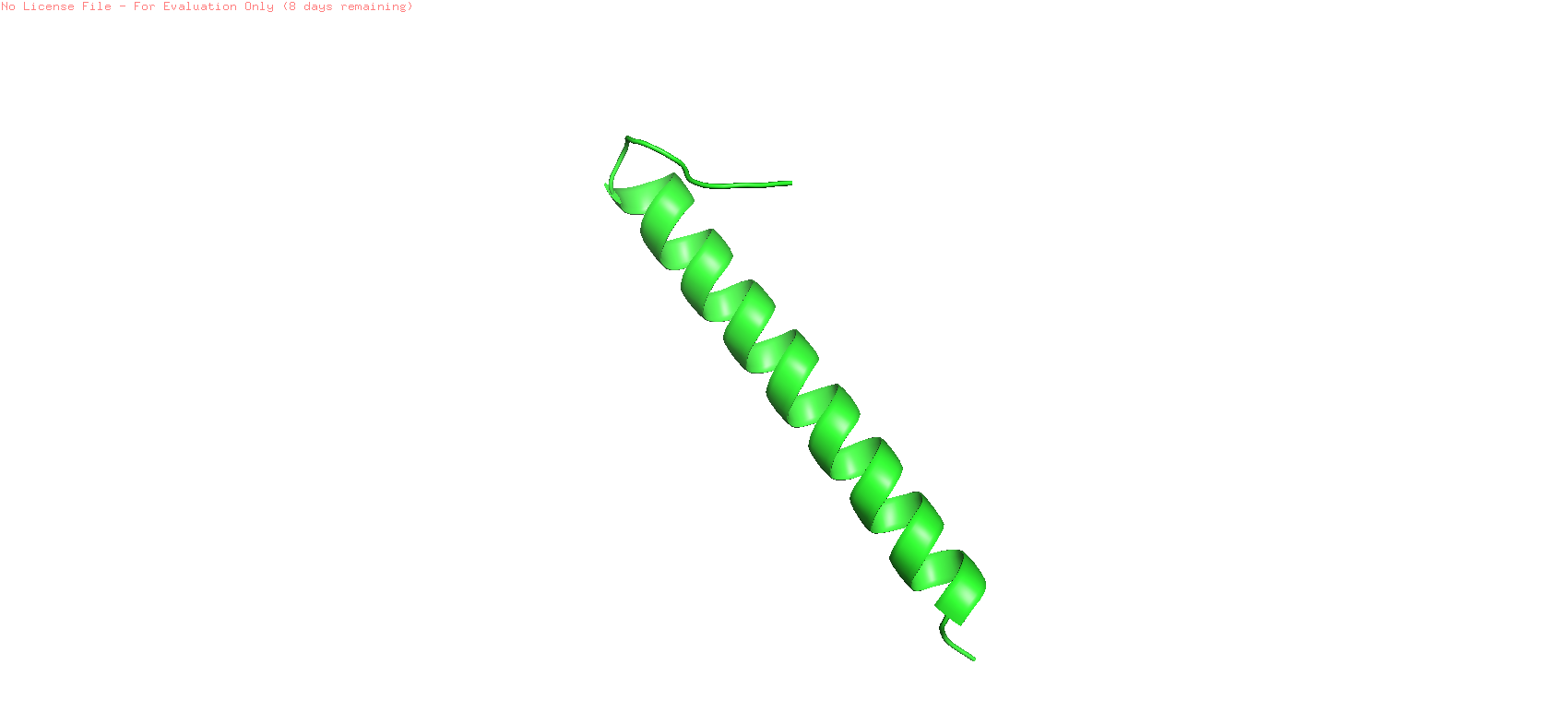

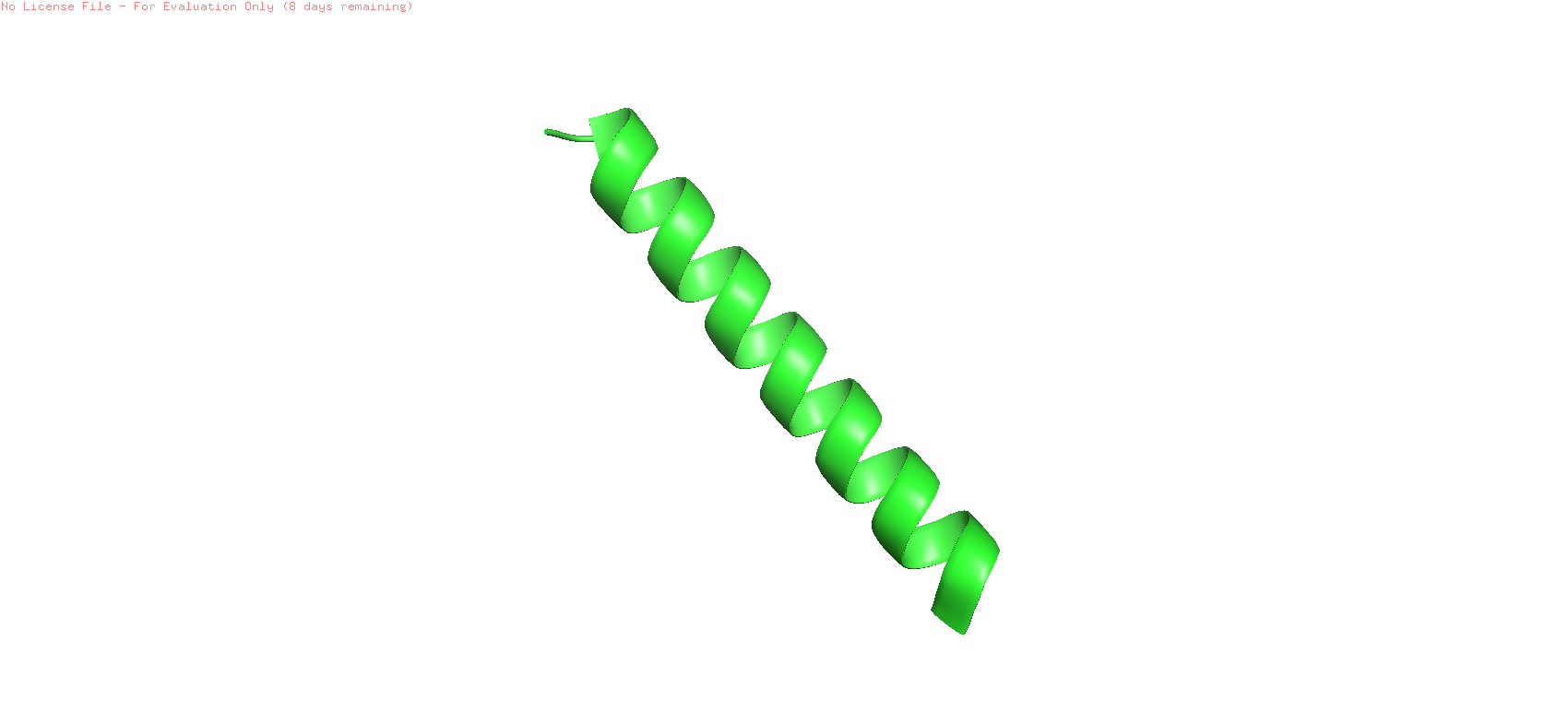
 P53 P54**

**
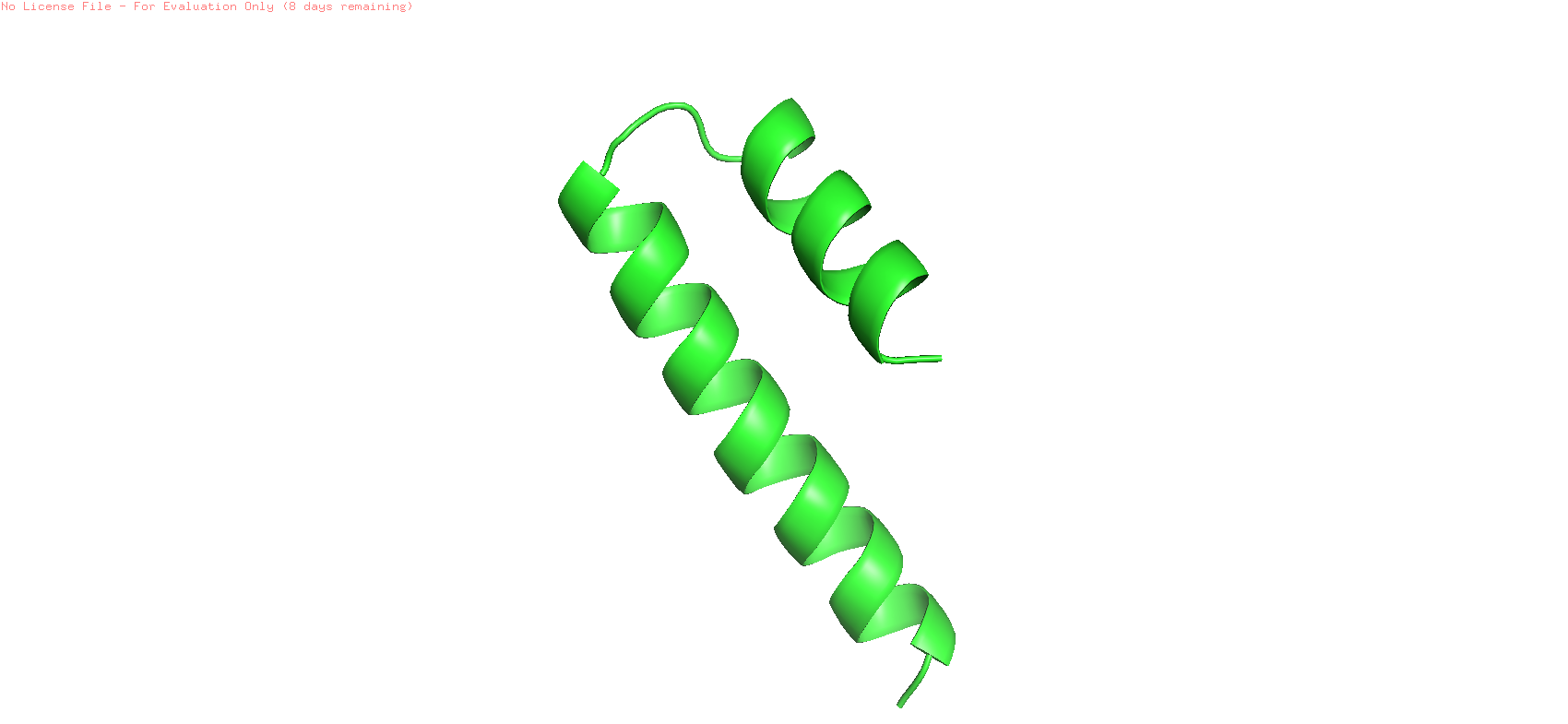

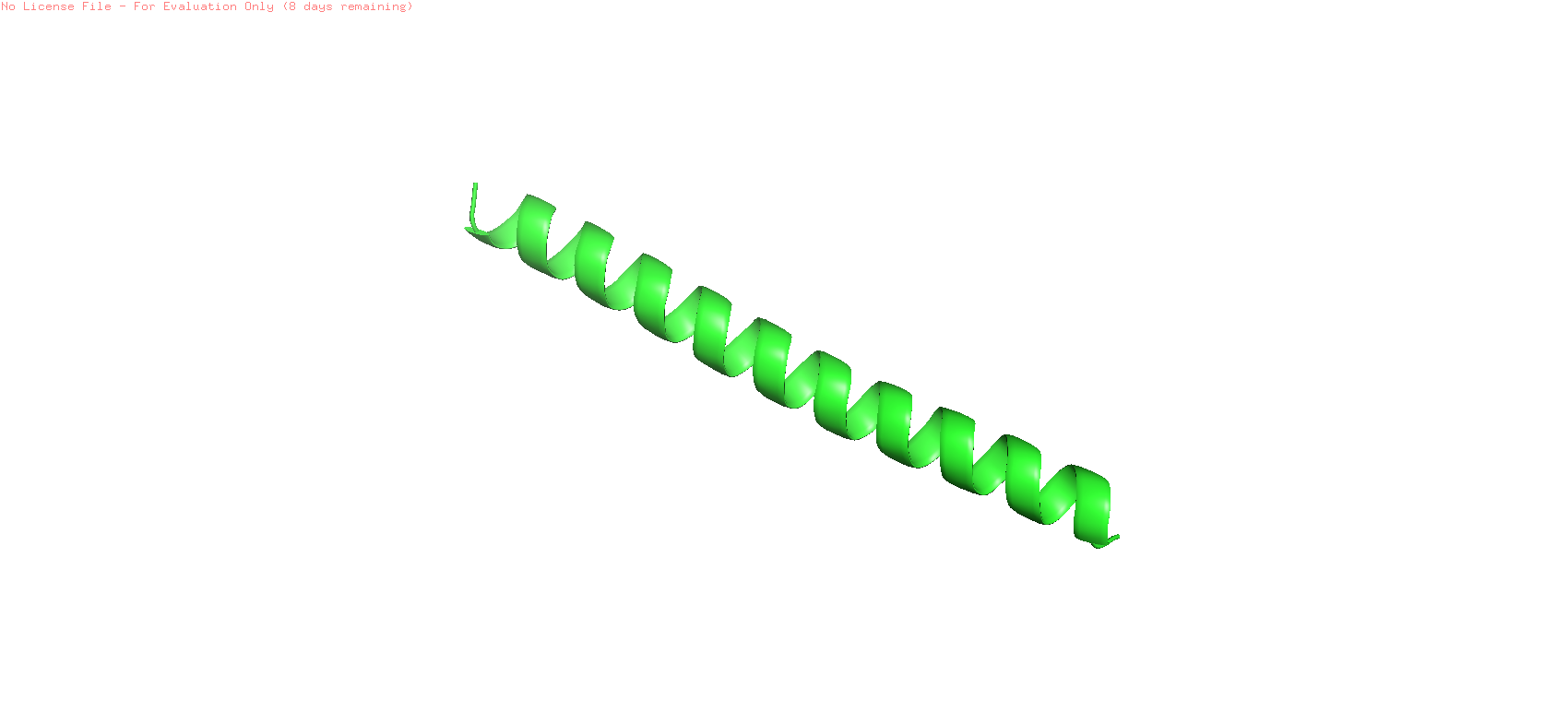
**

**P55 P56**

**
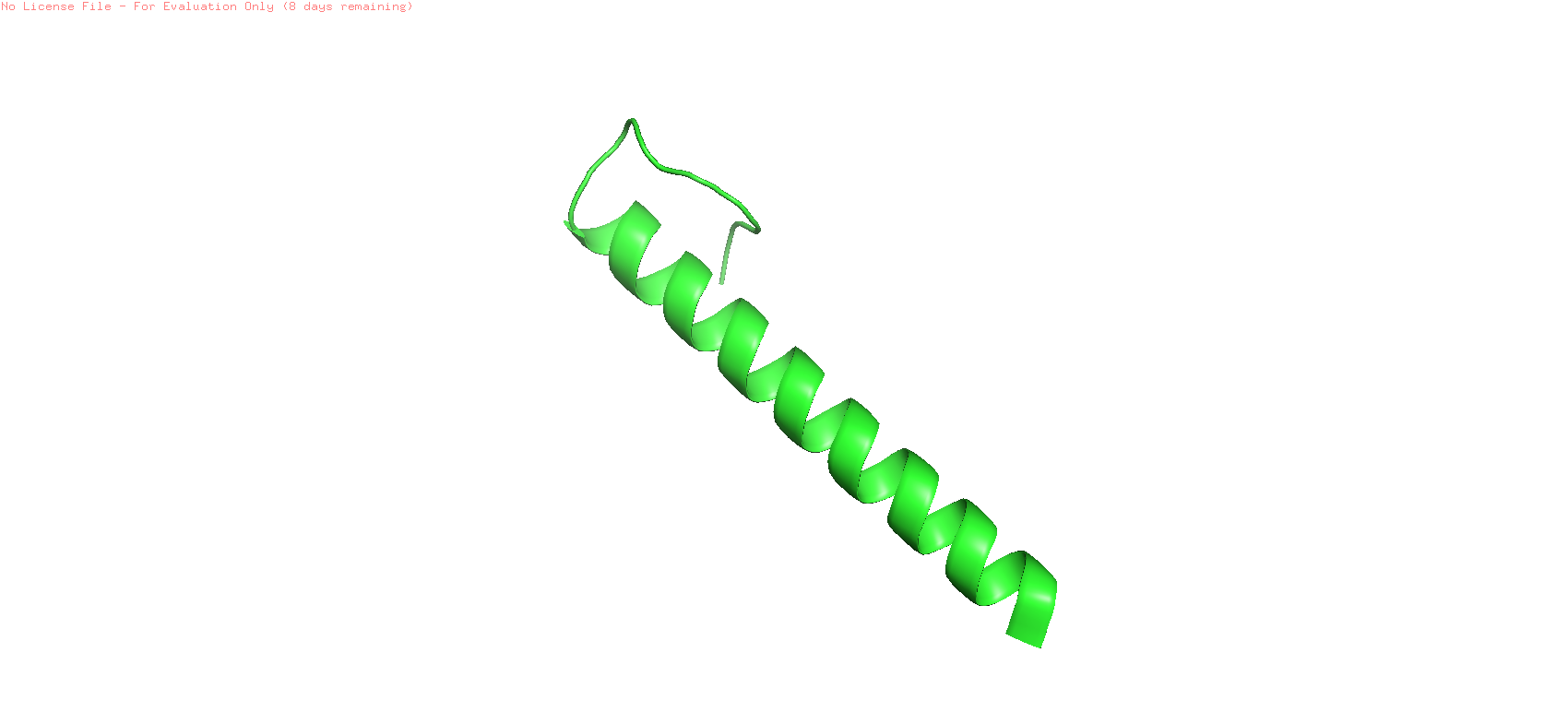

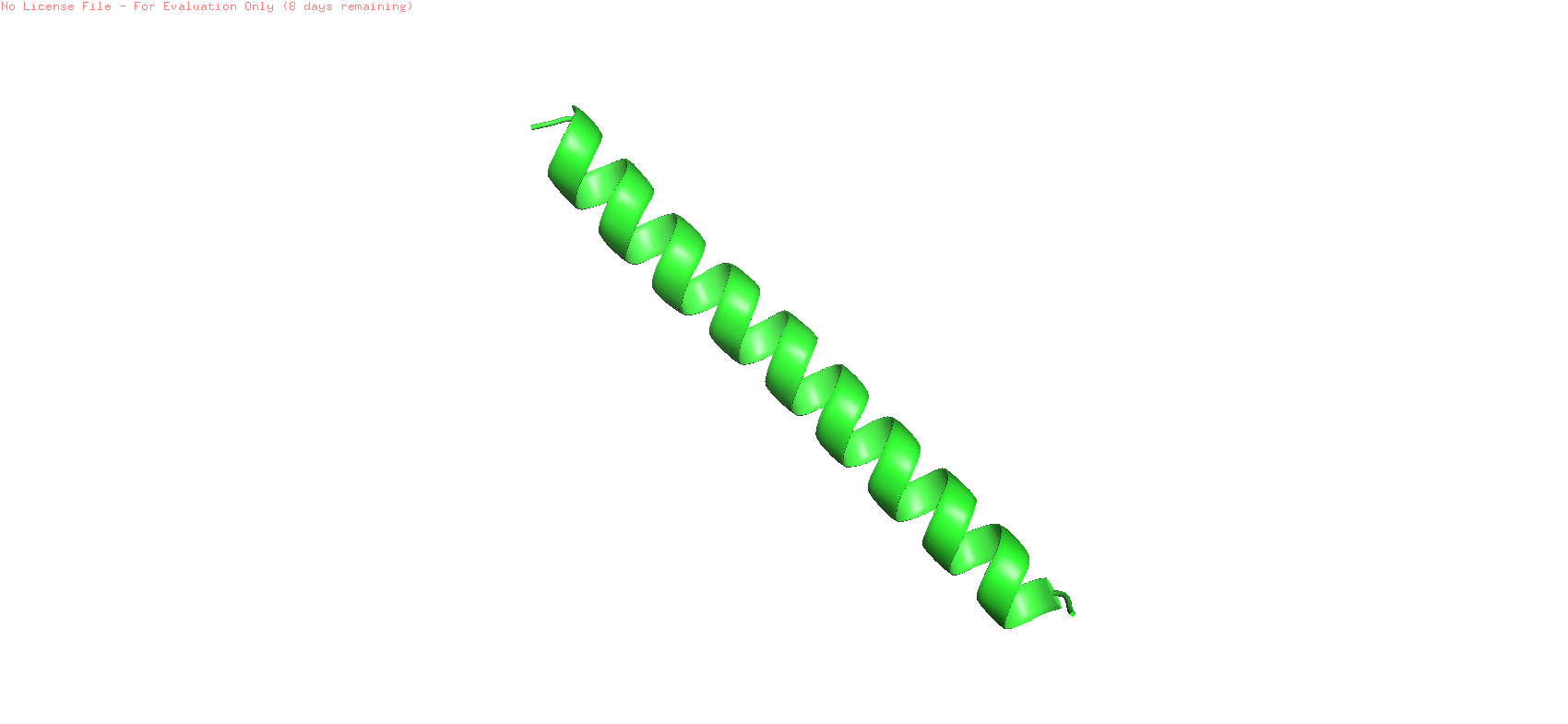
 P57 P58**

**
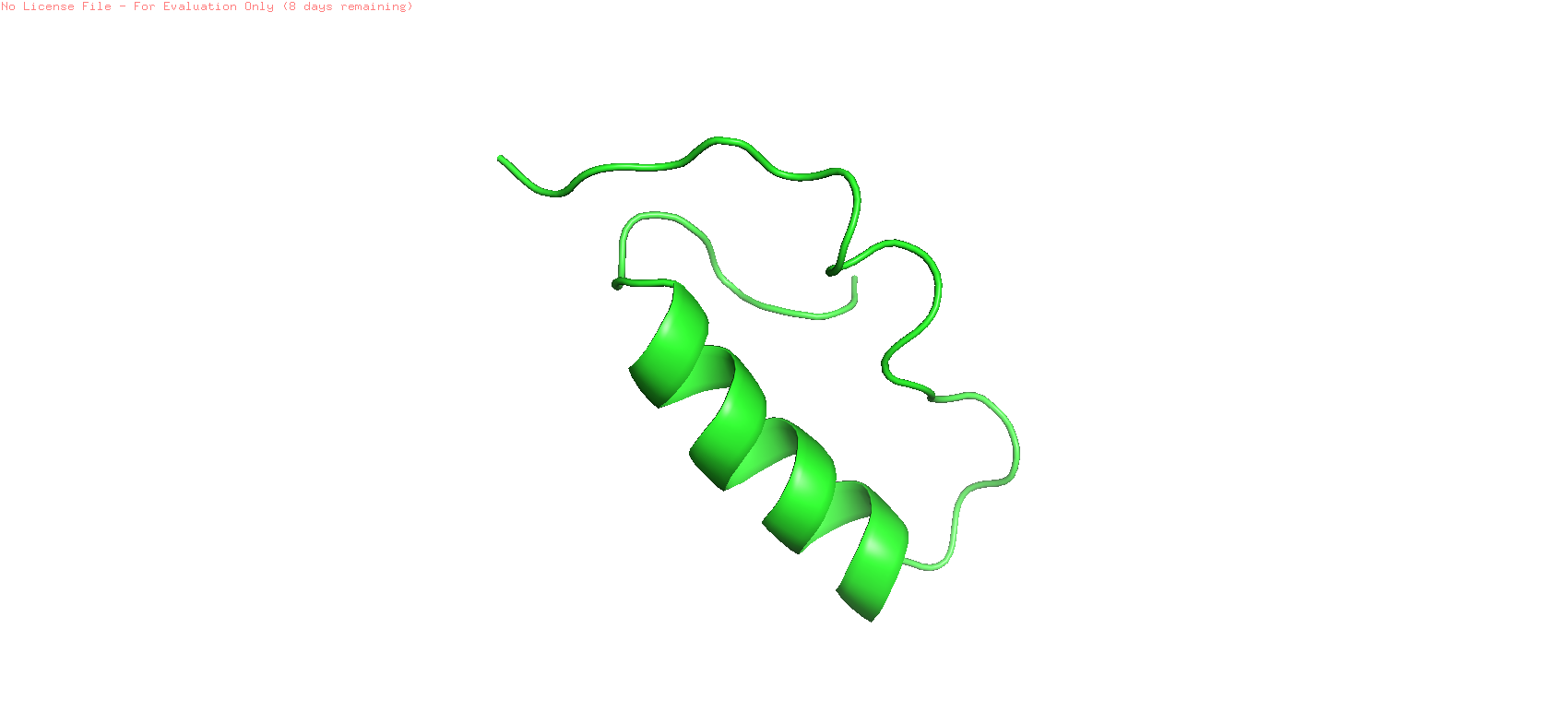
**

**P59**

**
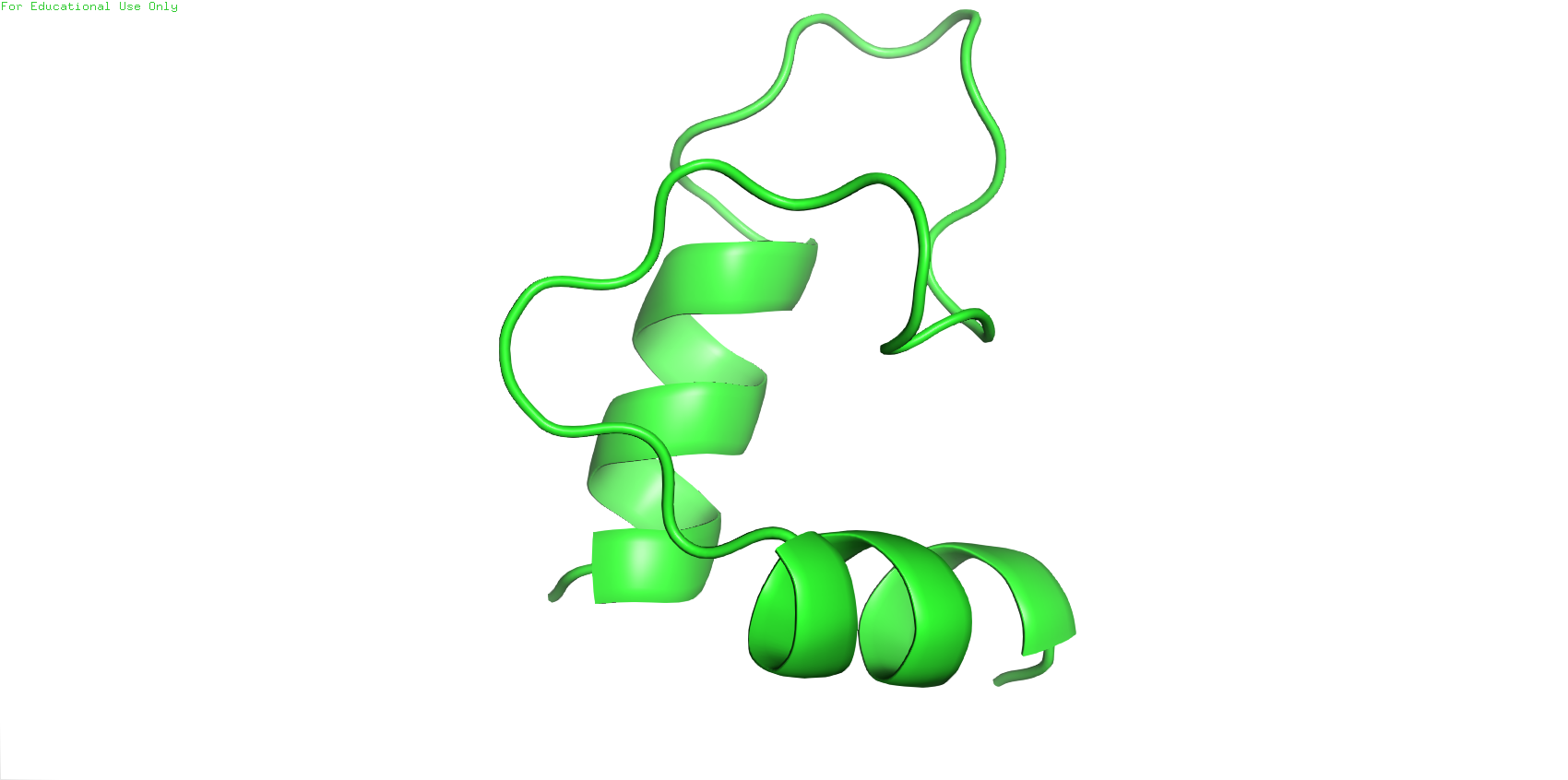

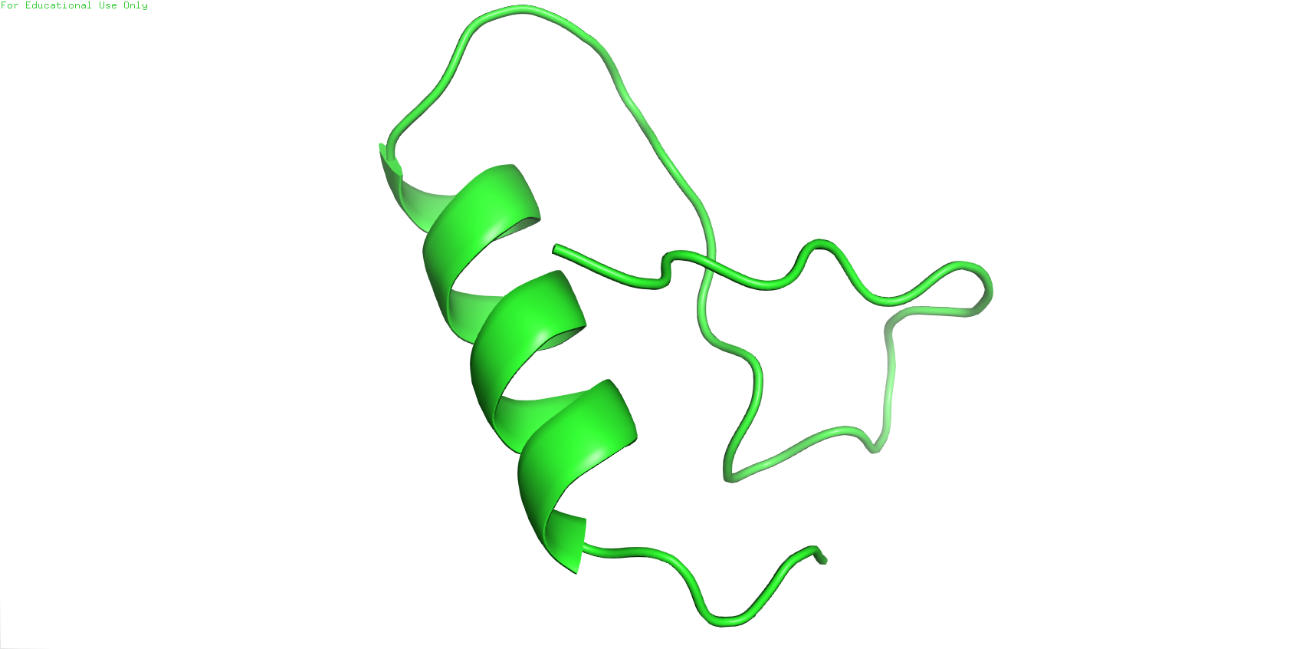
 P60 P61**

**
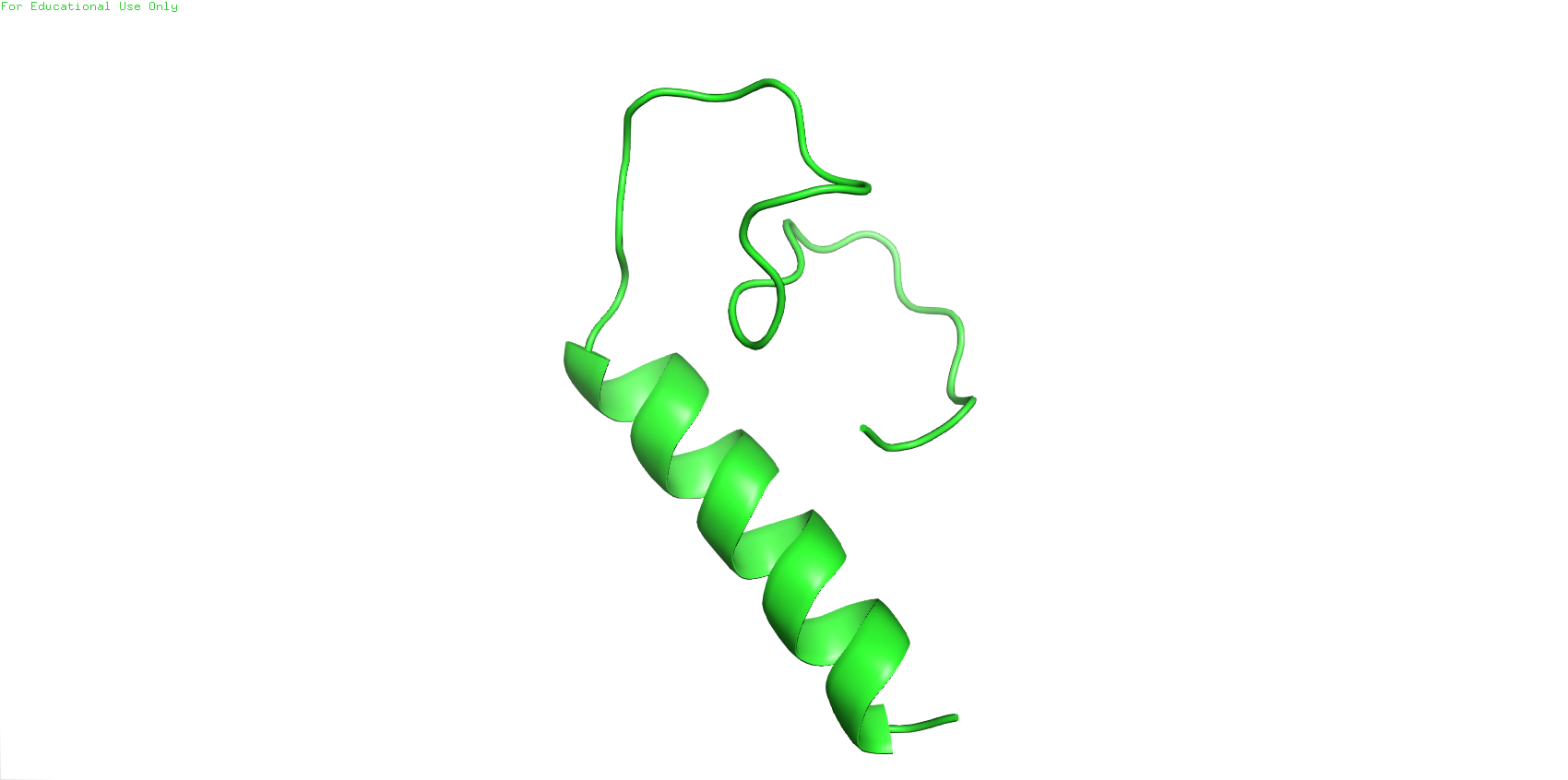

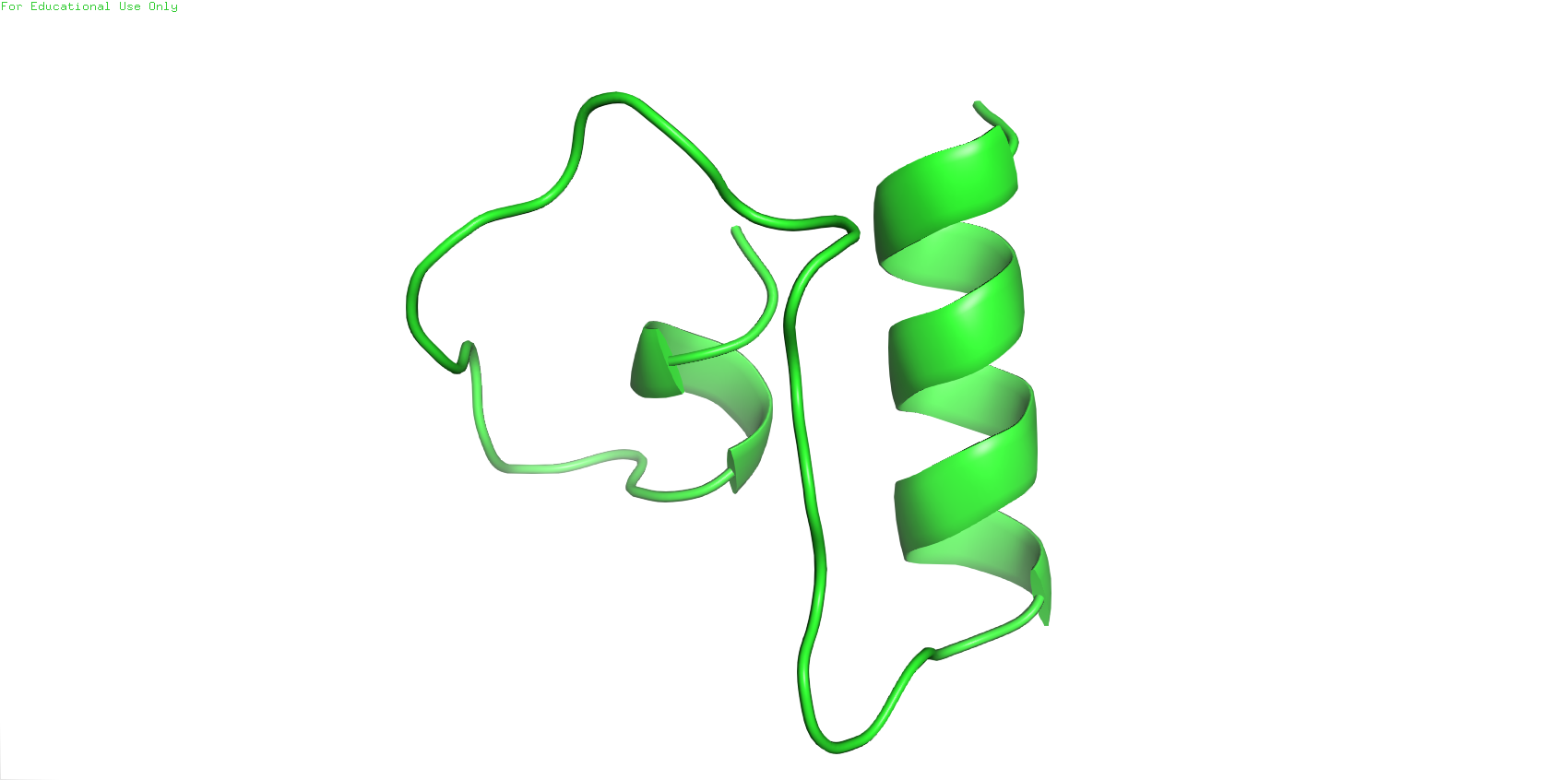
**

**P62 P63**

**
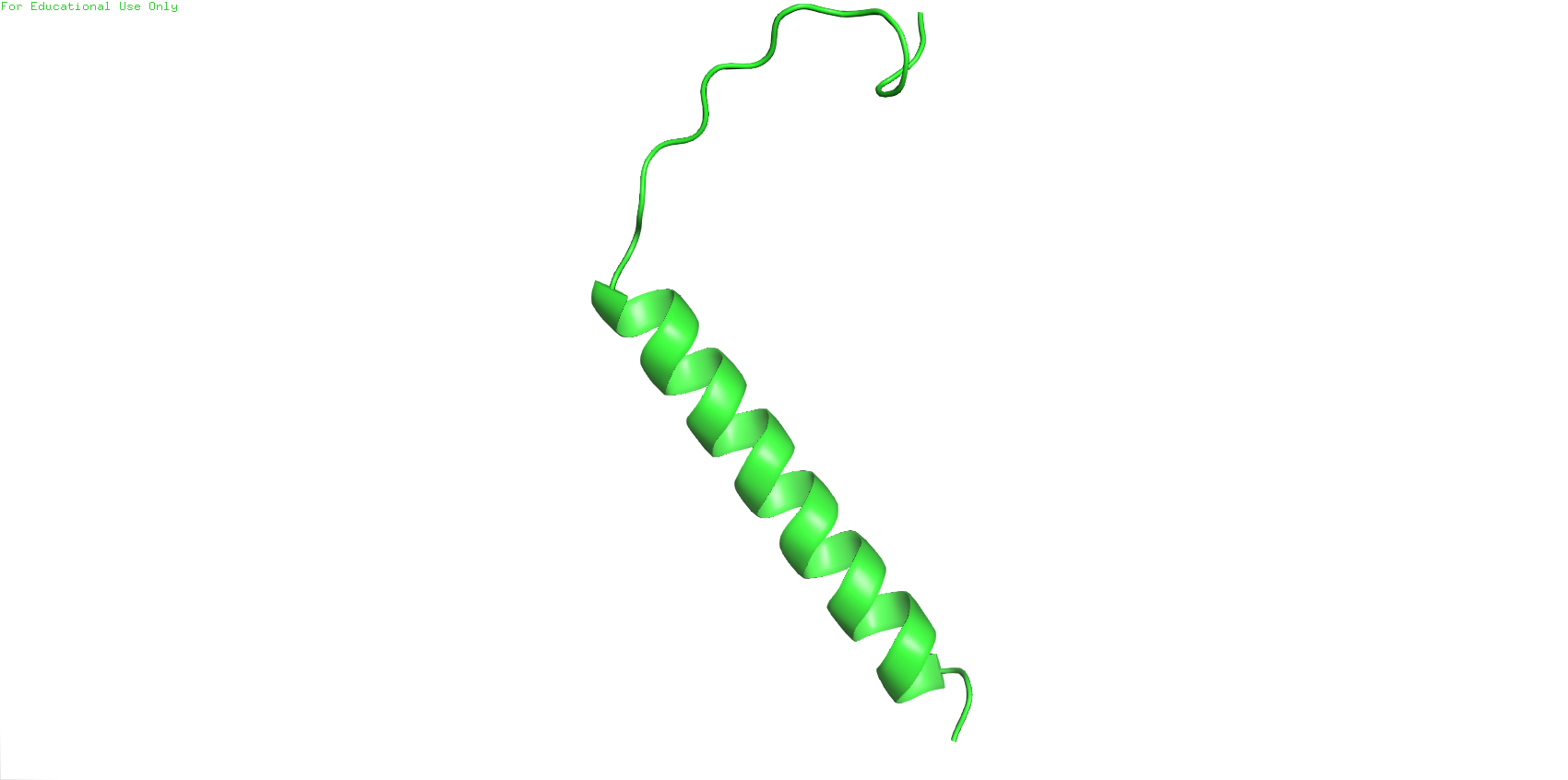

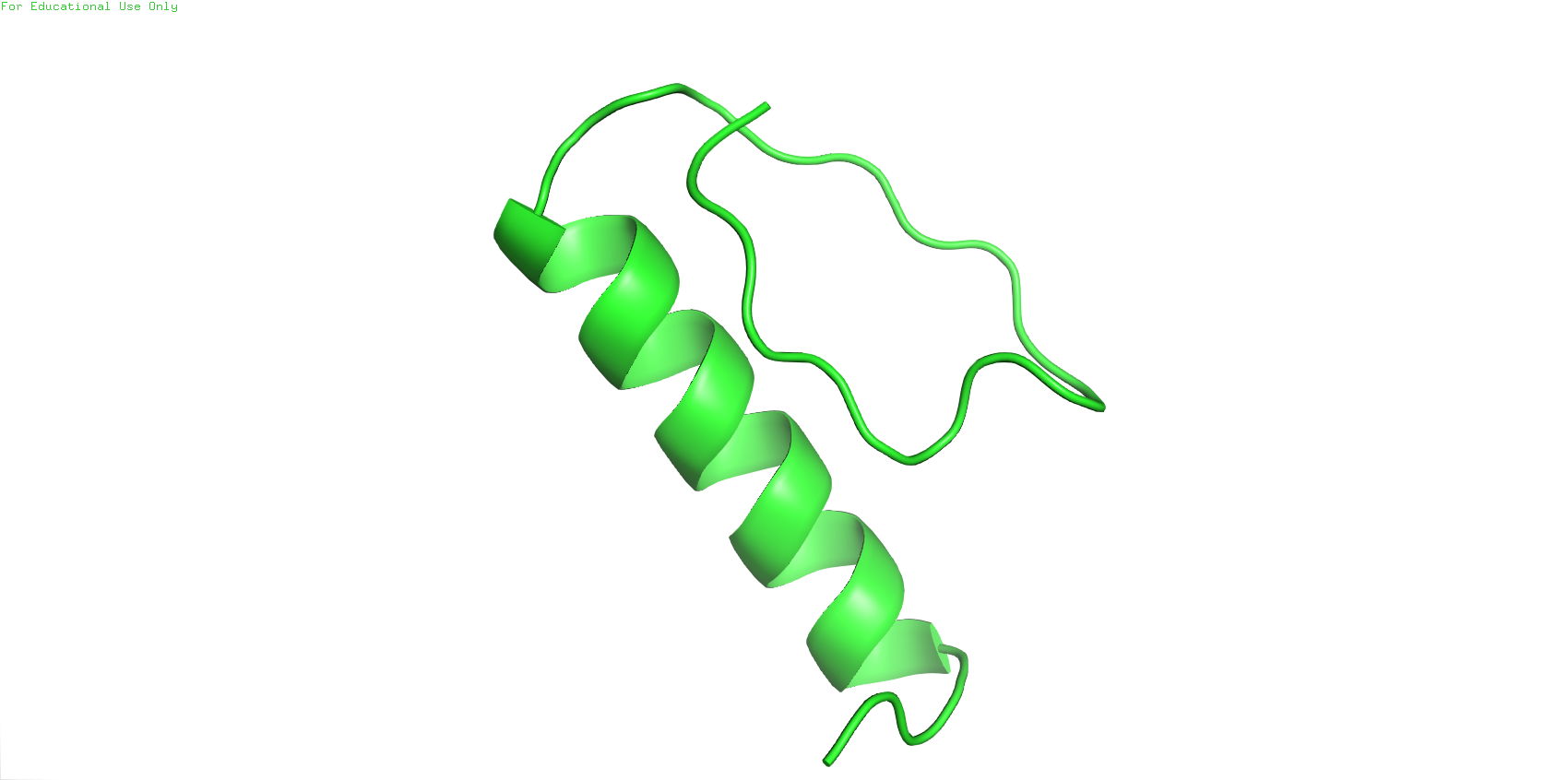
 P64 P65**

**
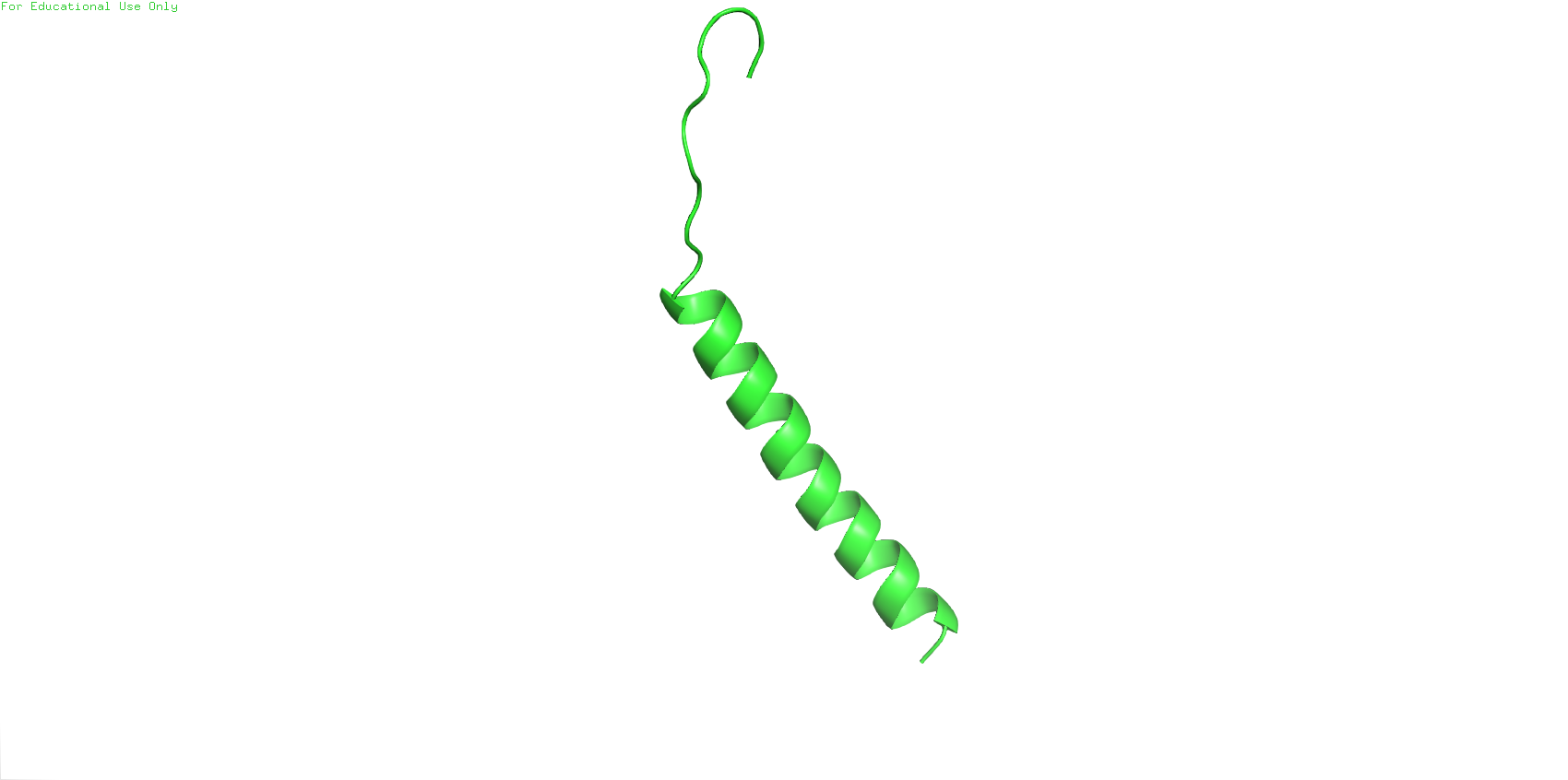
**


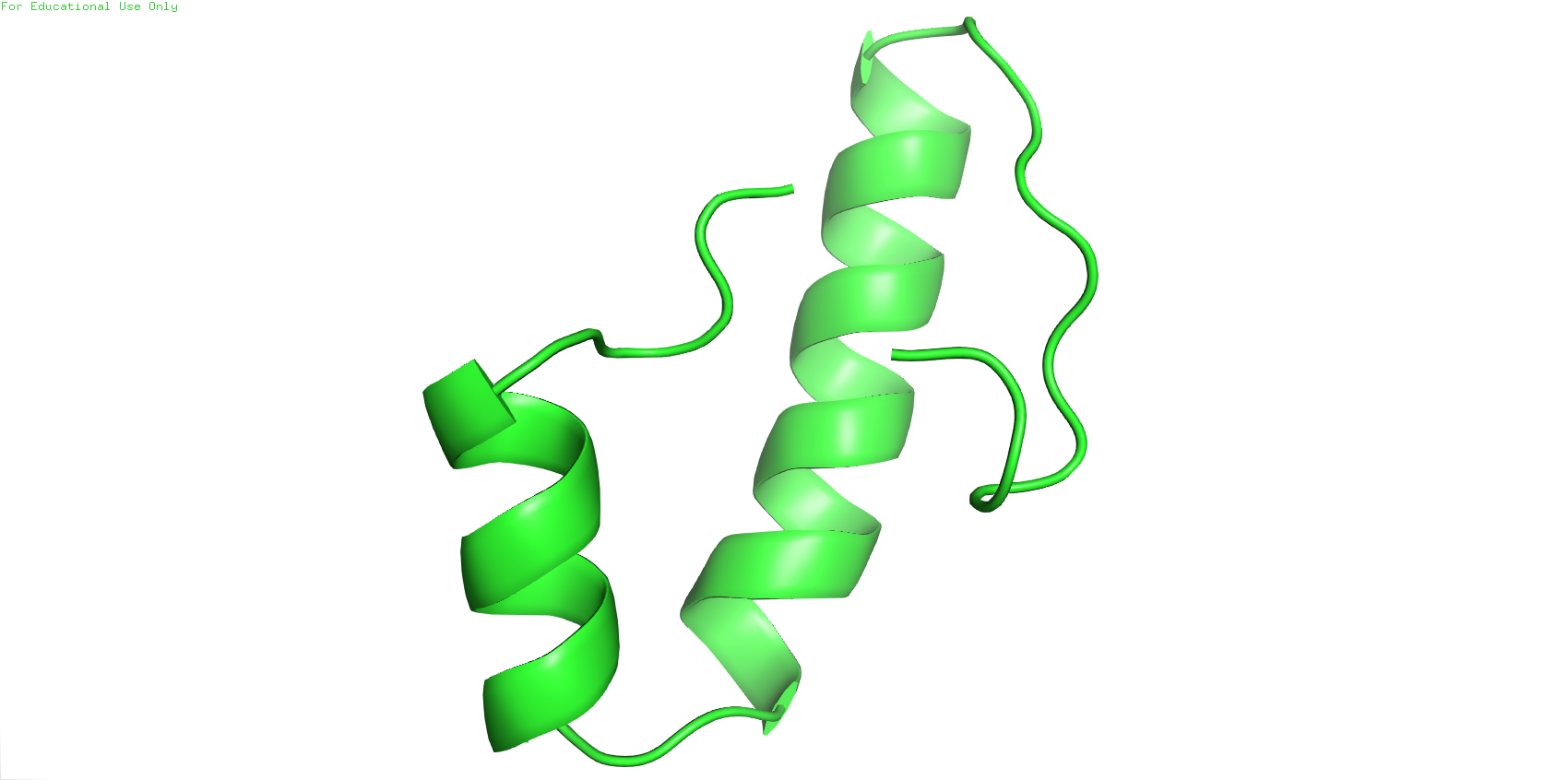


**P66 P67**

**
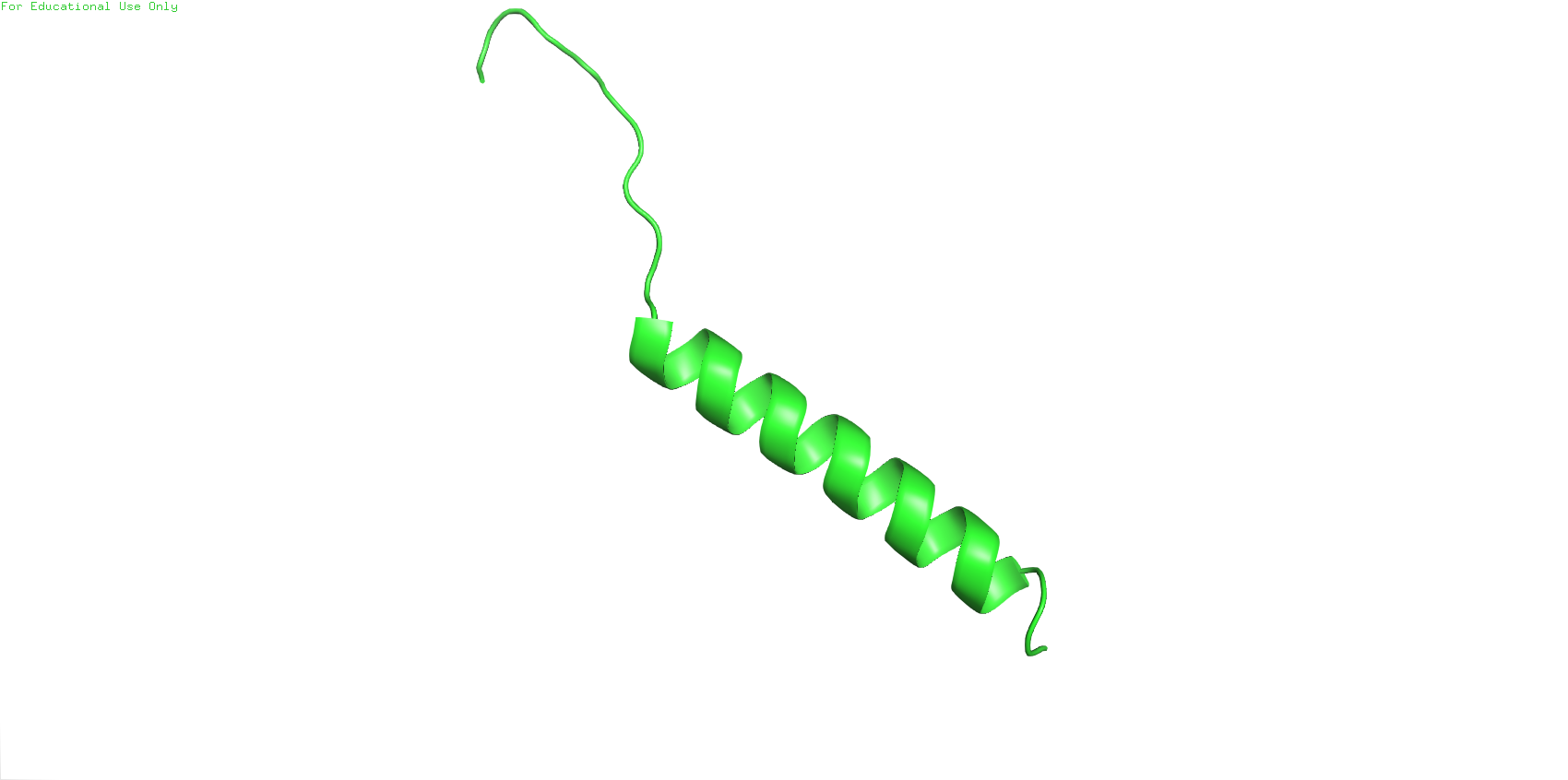

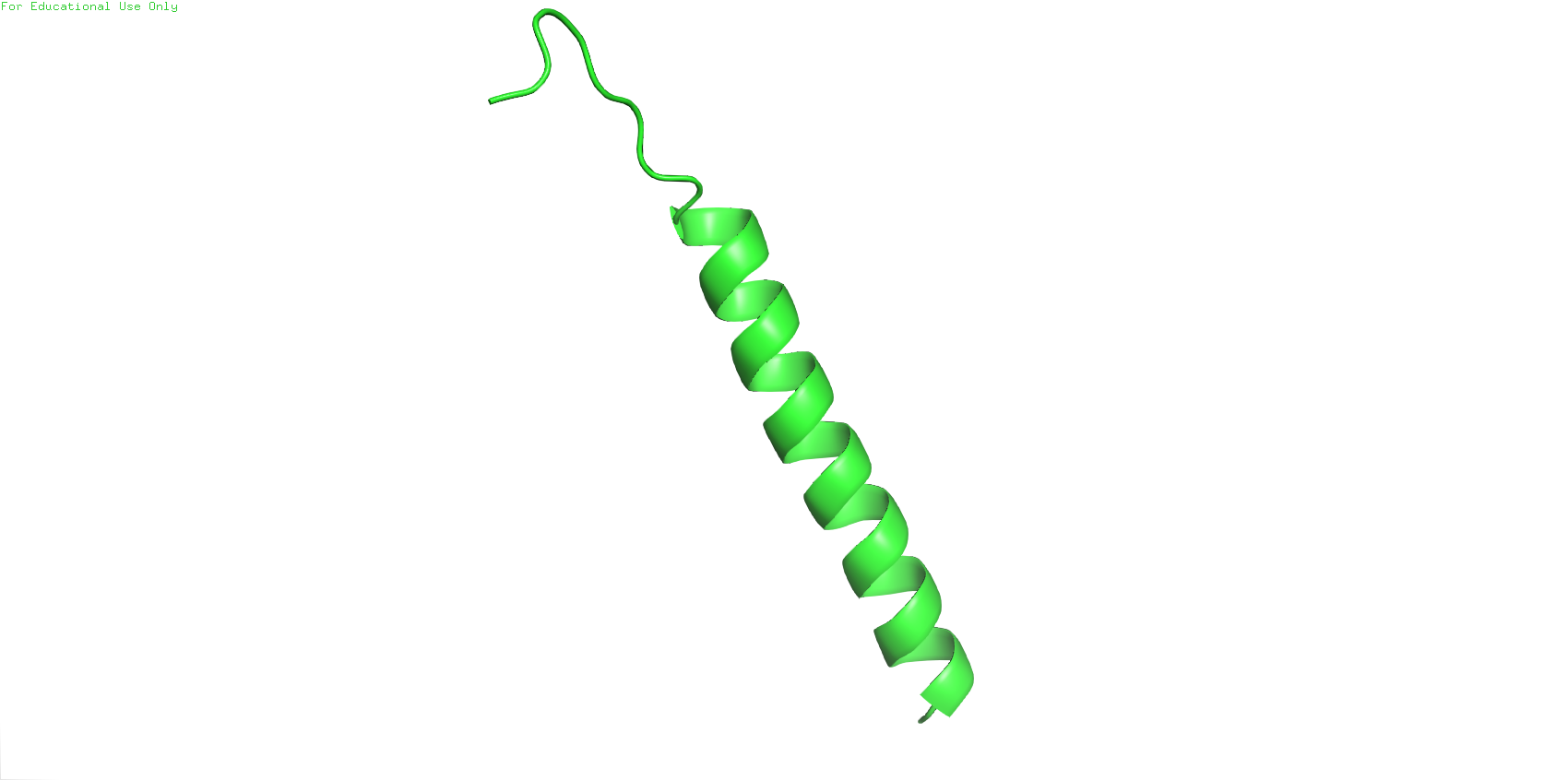
**

**P68 P69**

**
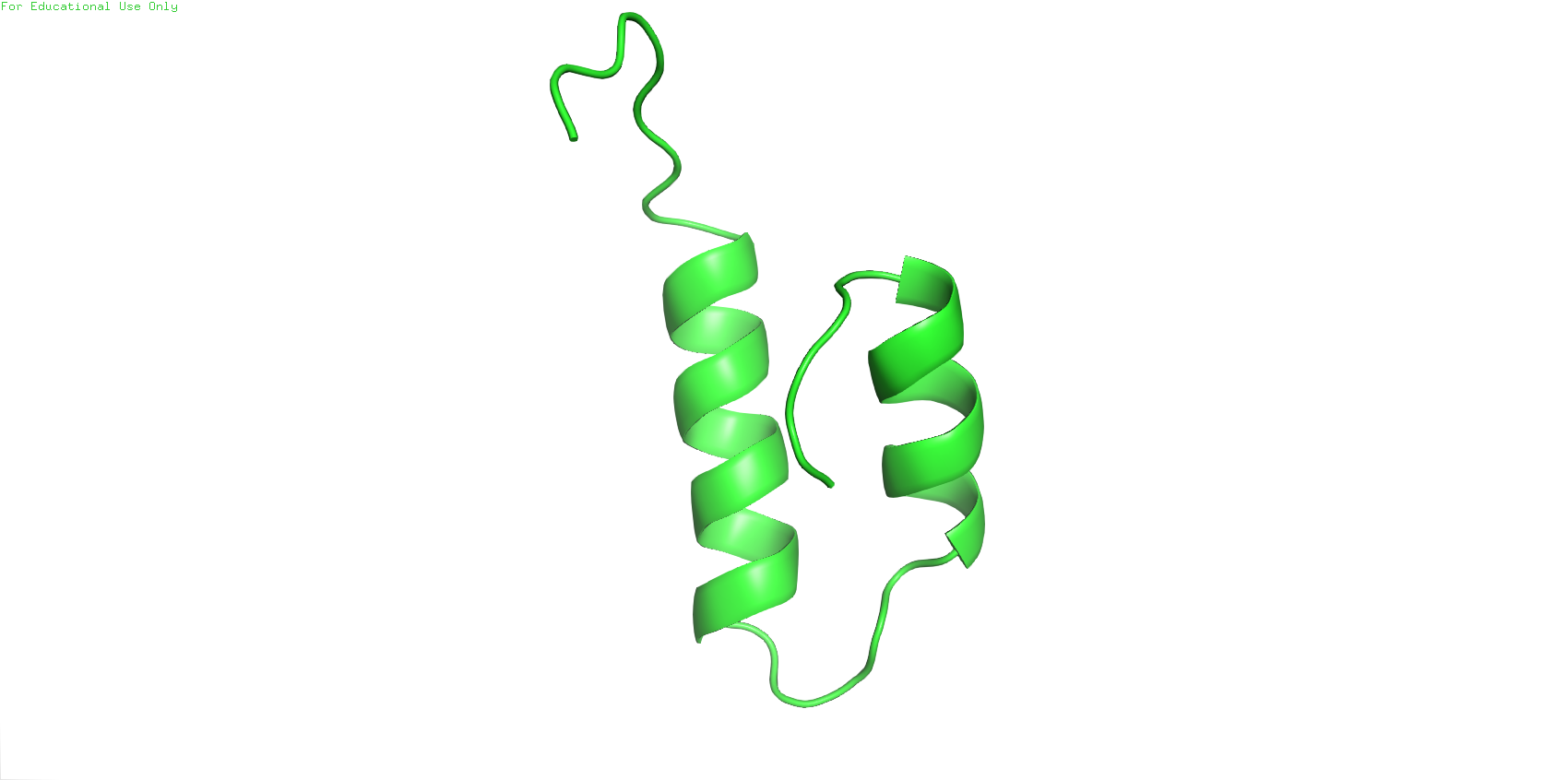
**

**
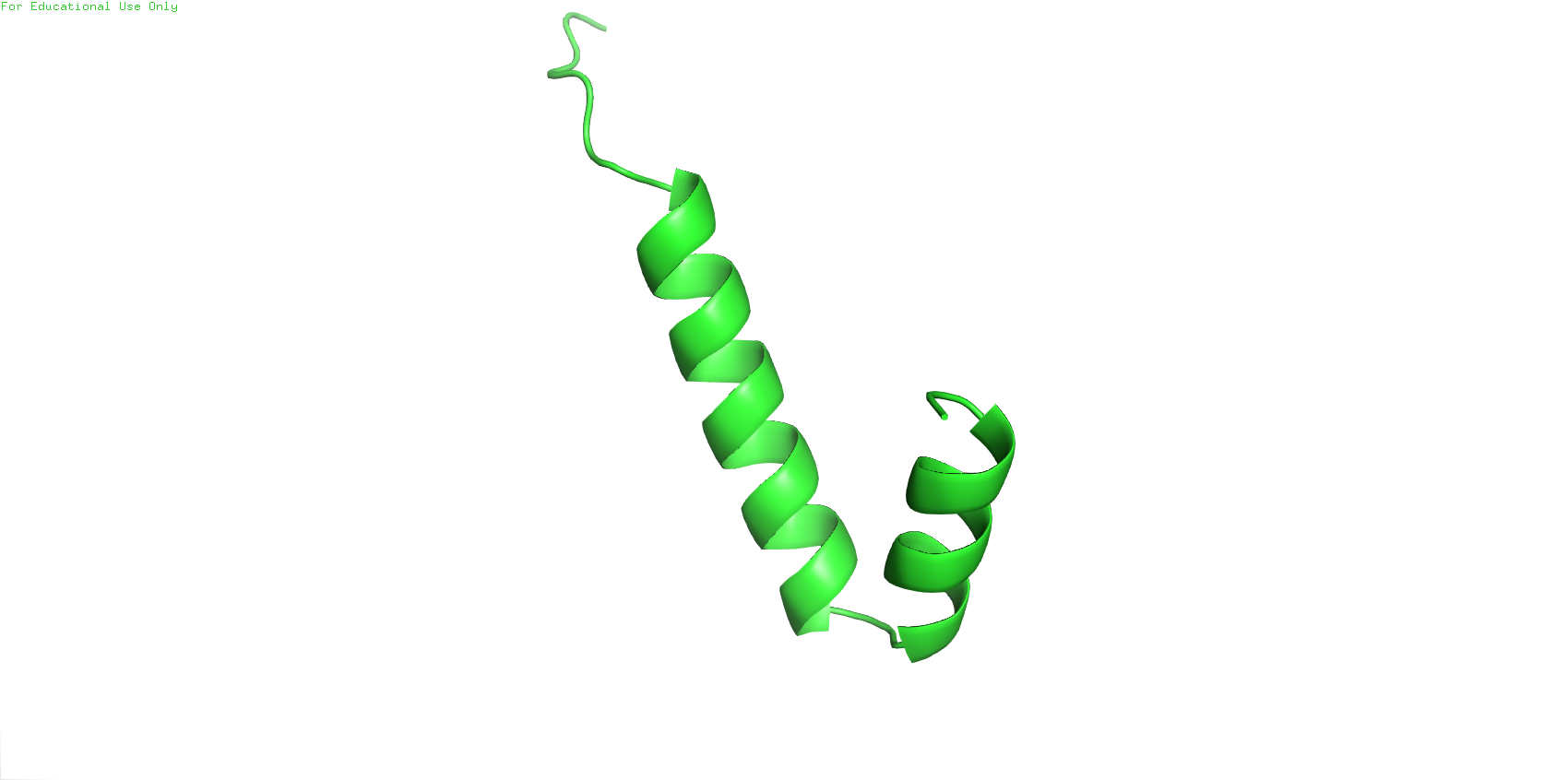
 P70 P71**

**
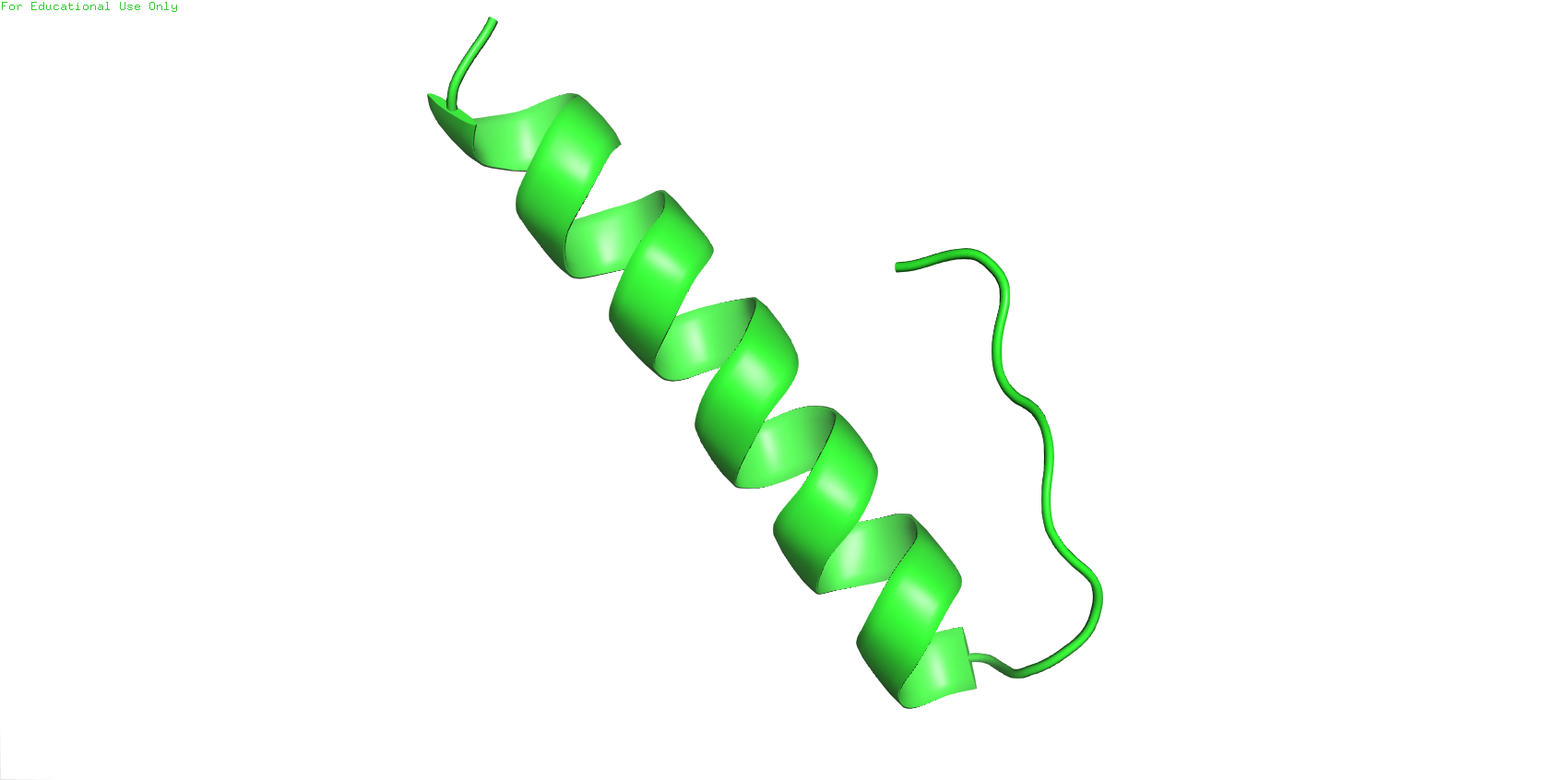

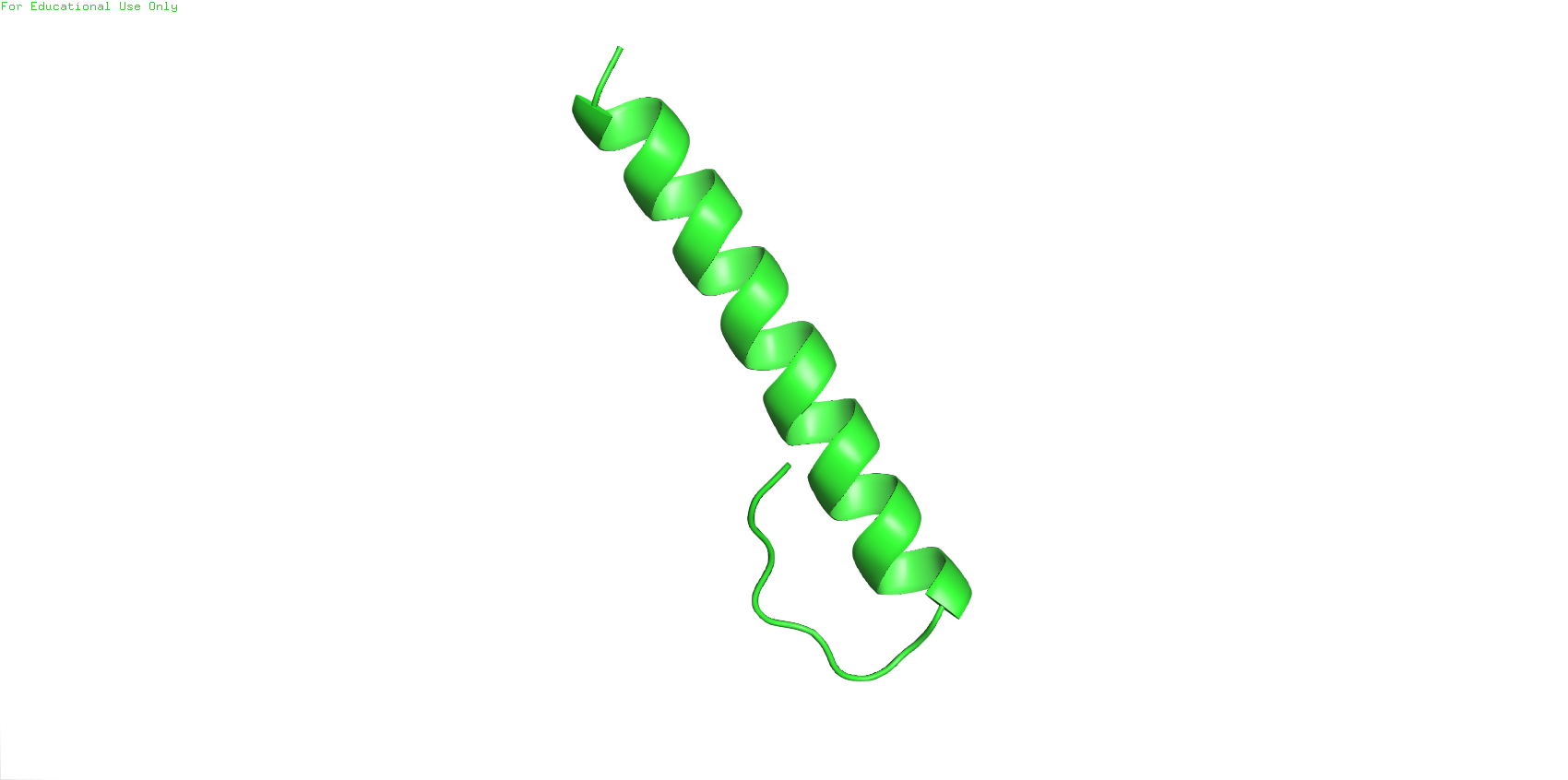
 P72 P73**

**
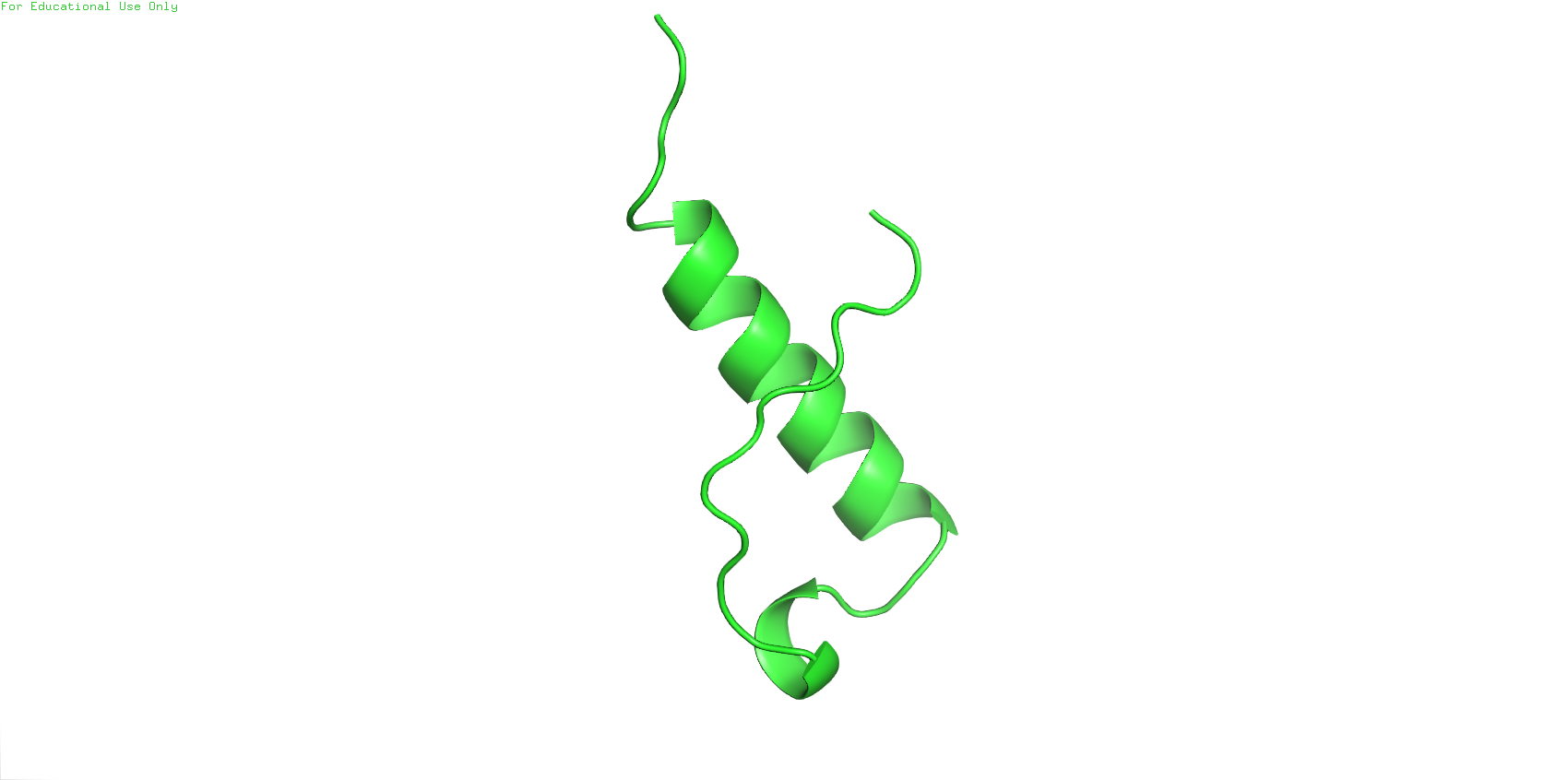

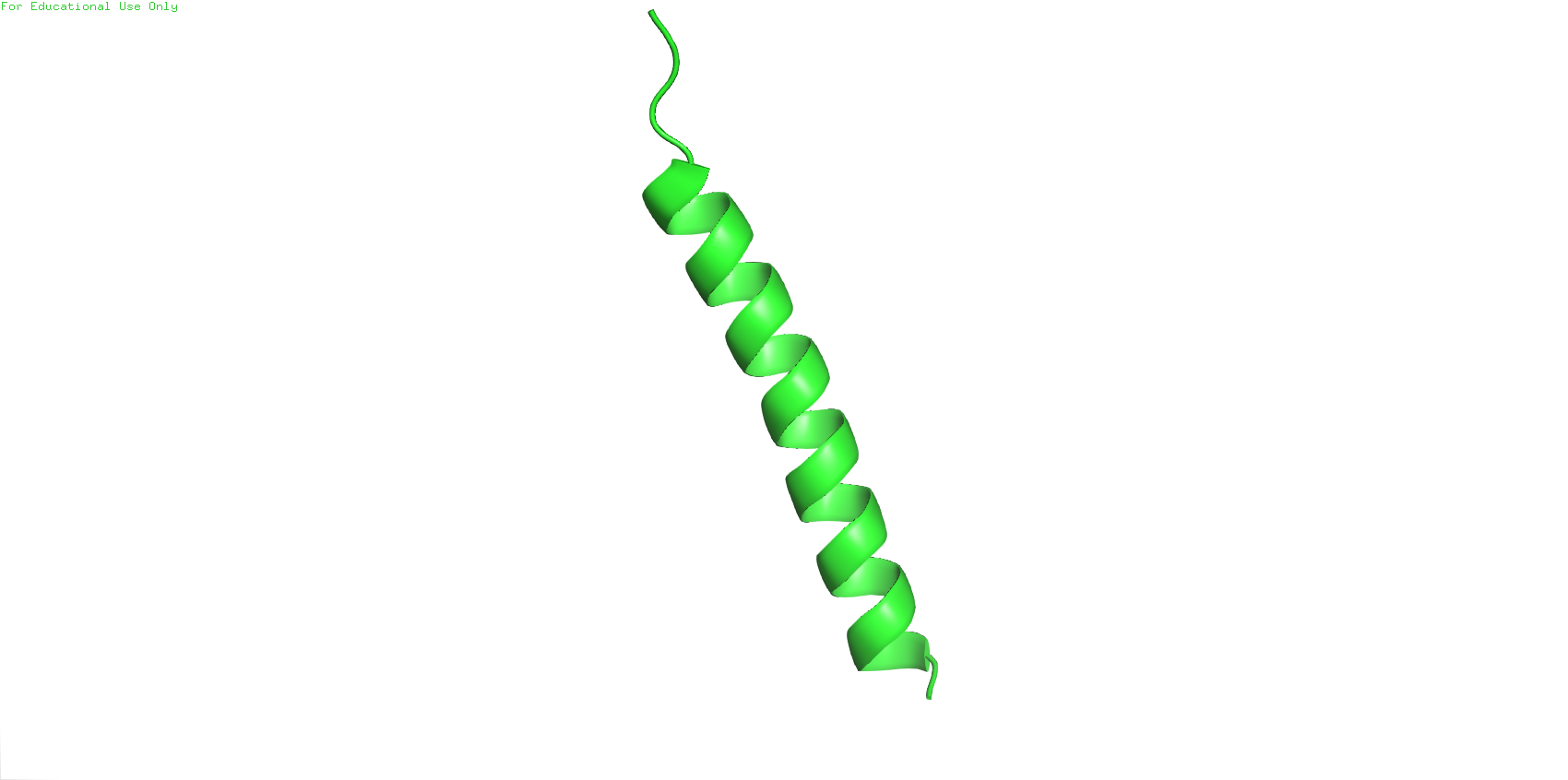
**

**P74 P75**

**
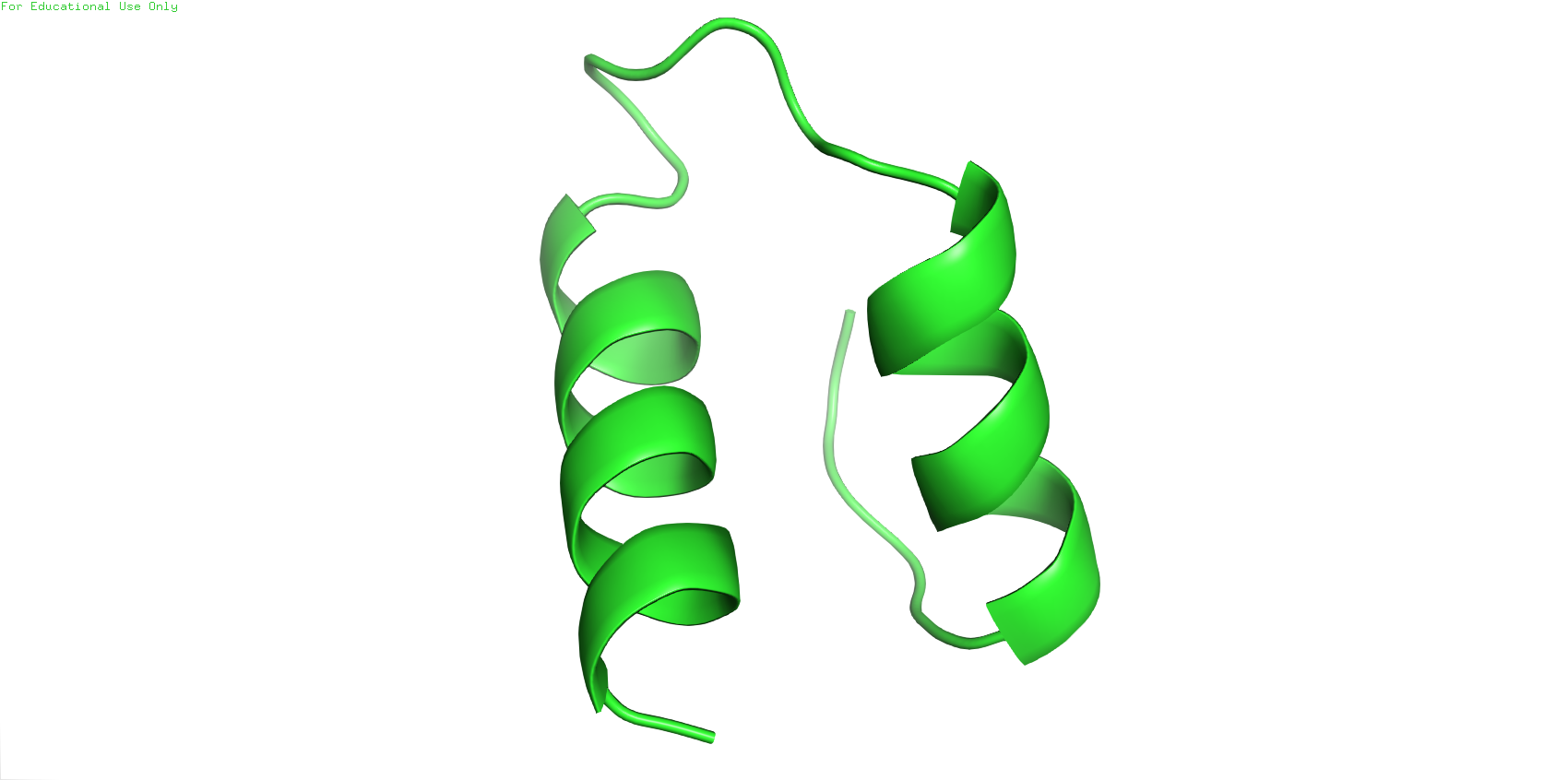

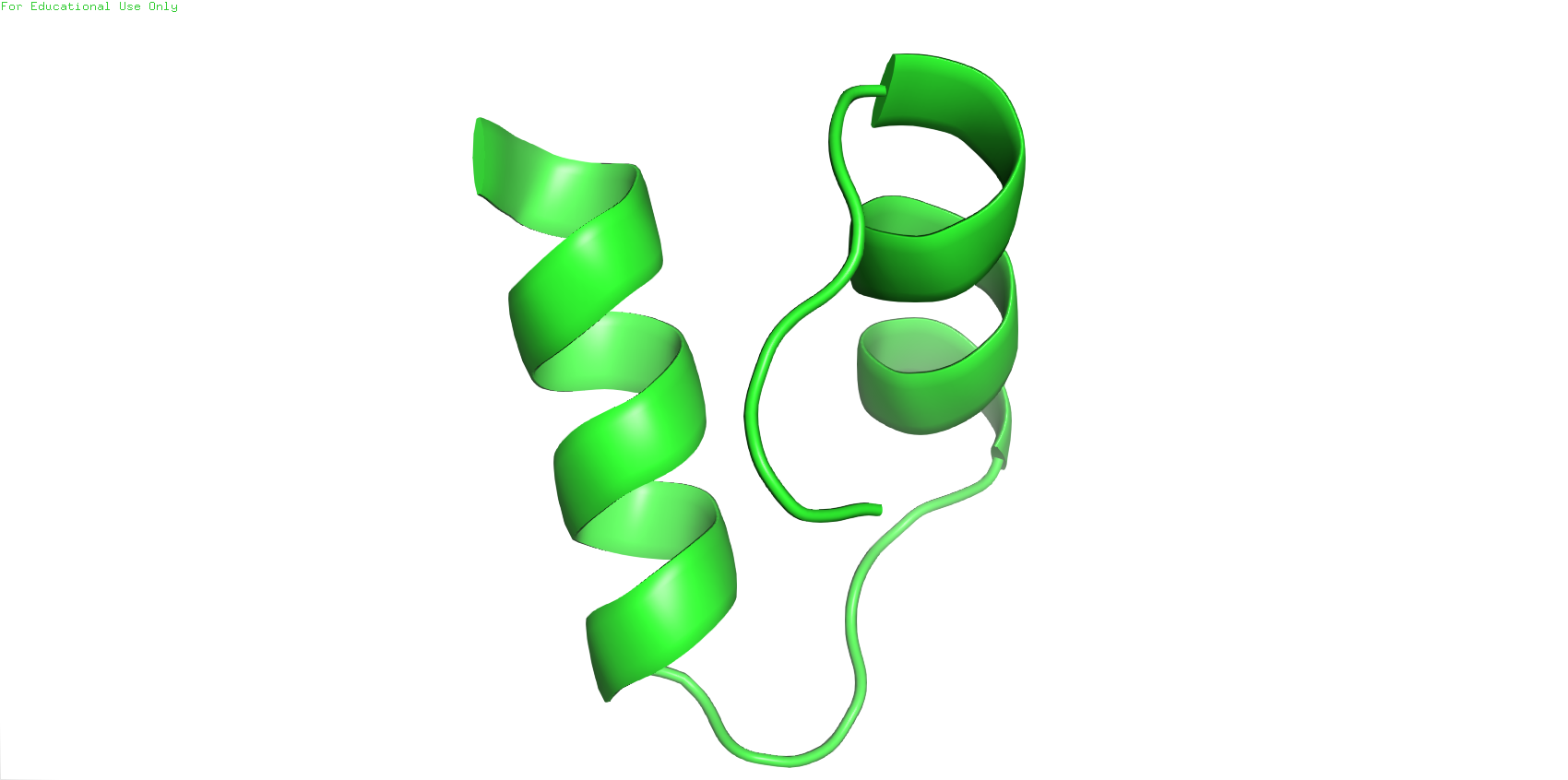
 P76 P77**

**
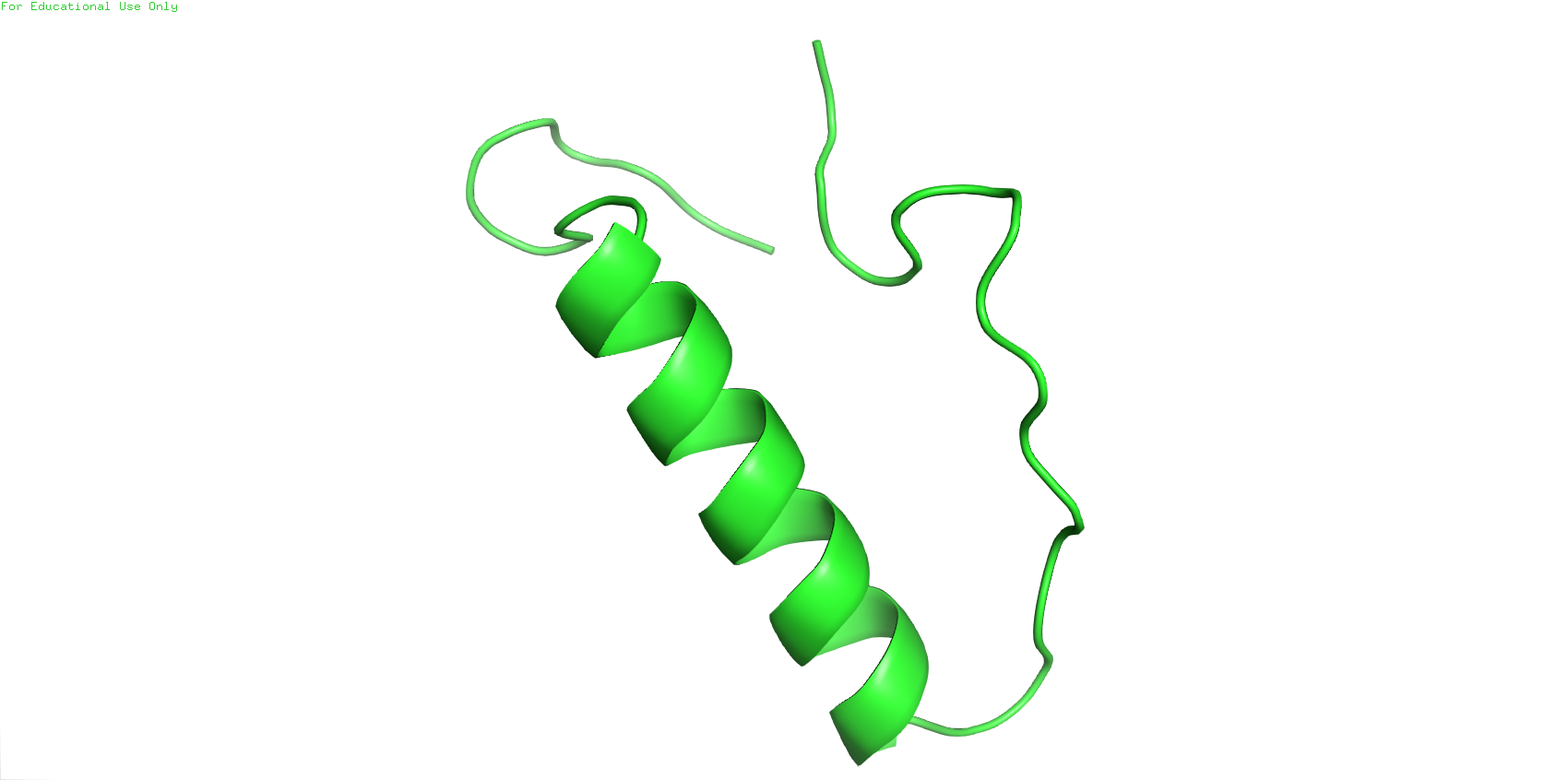
**
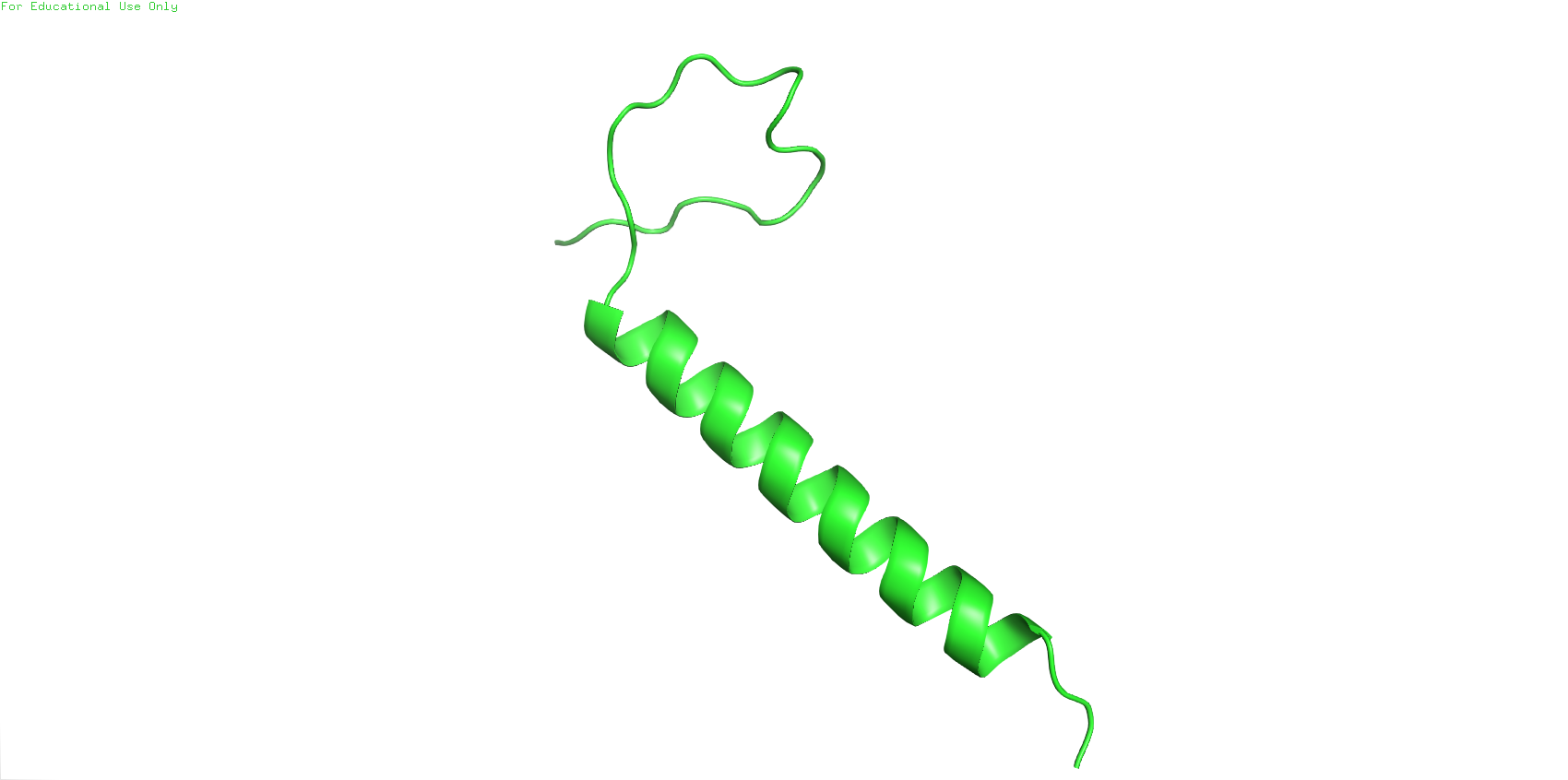


**P78 P79**

**
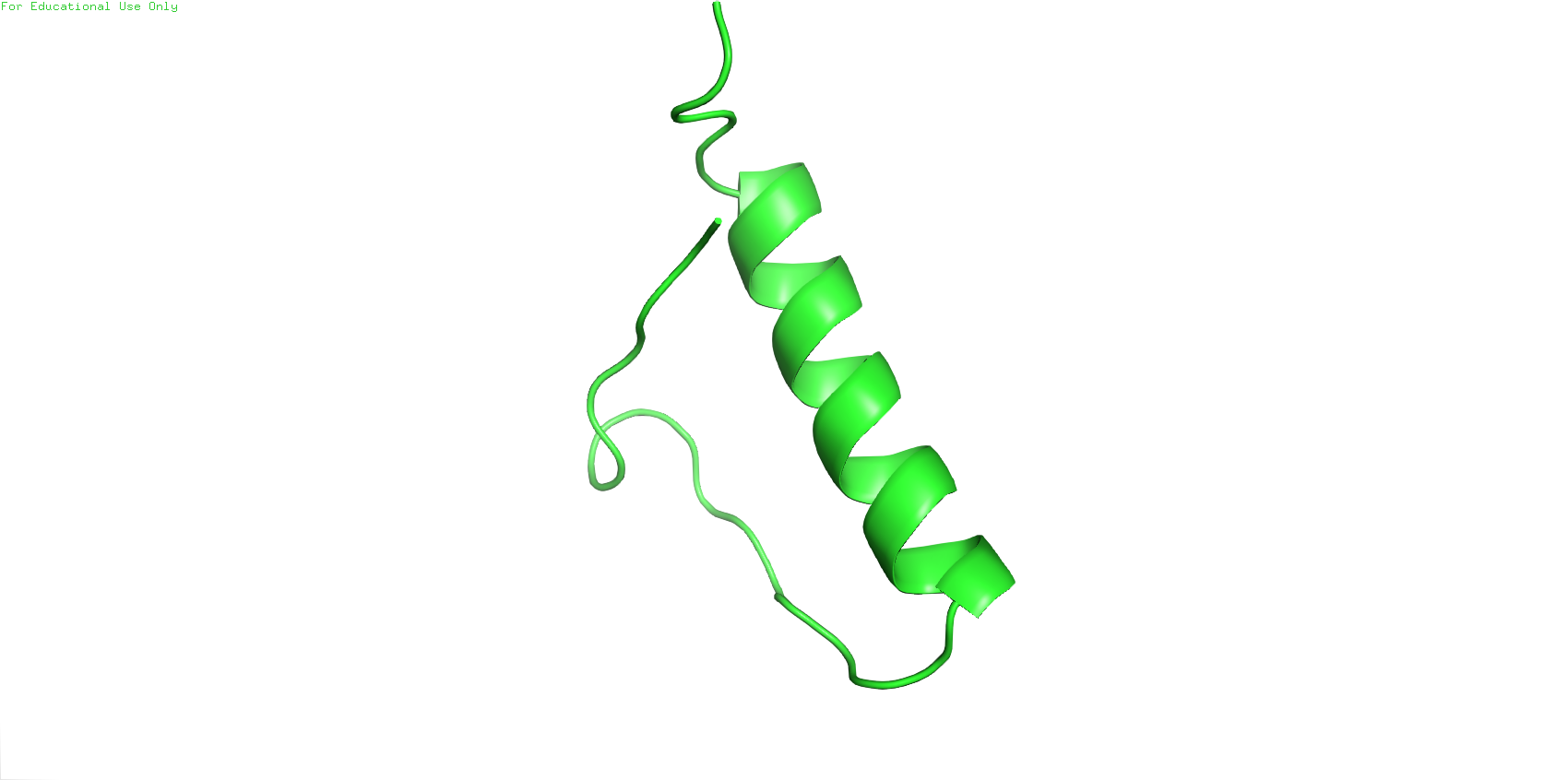
**

**
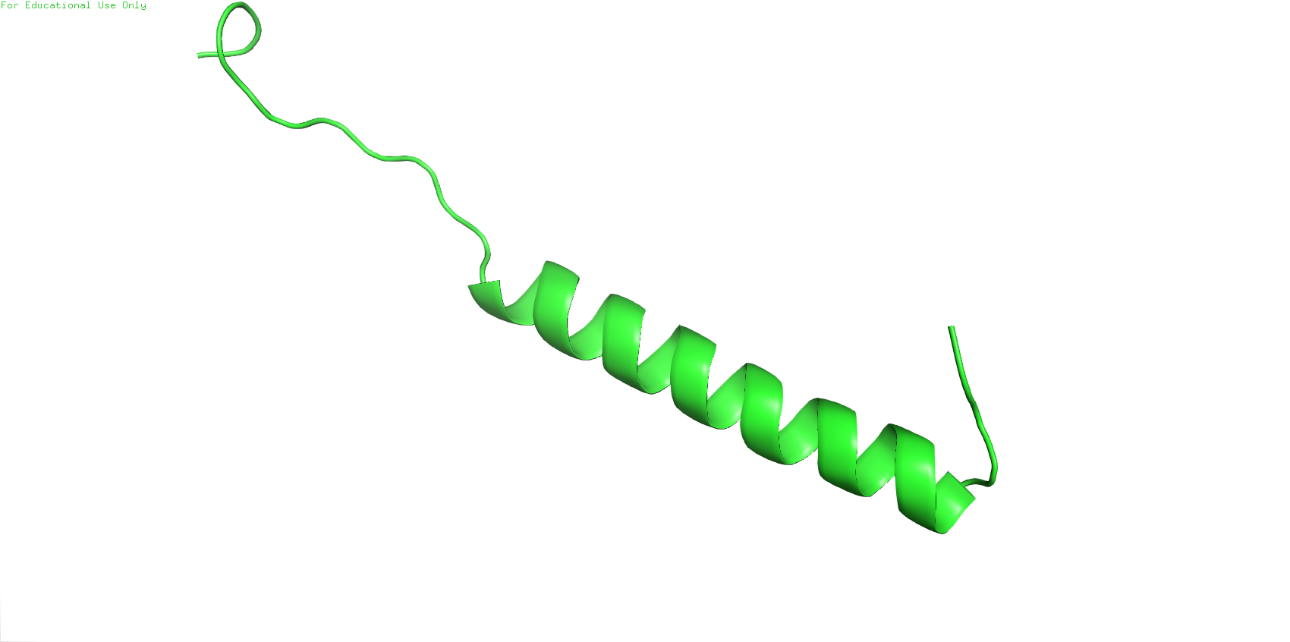
**

**P80 P81**

**
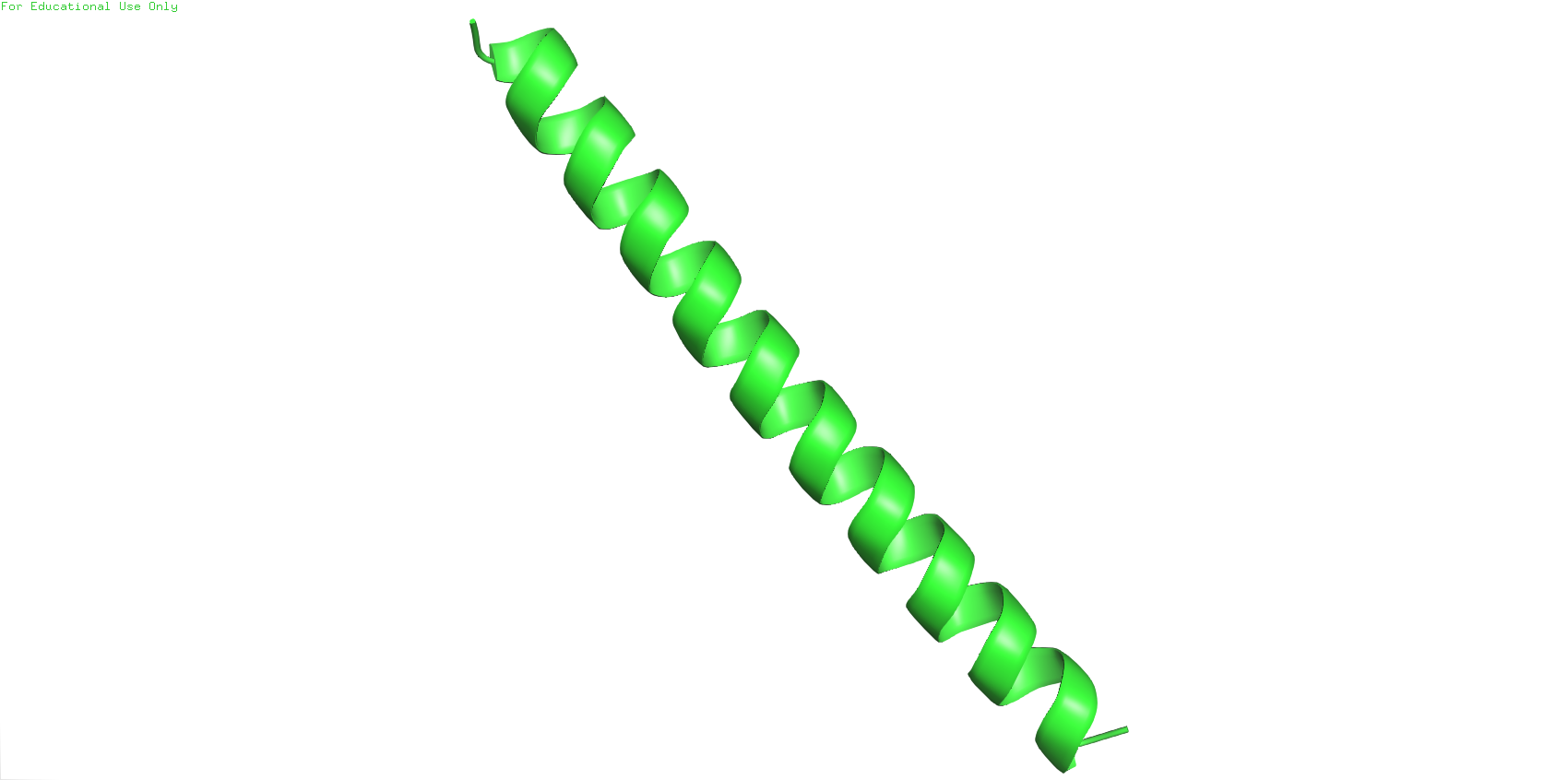

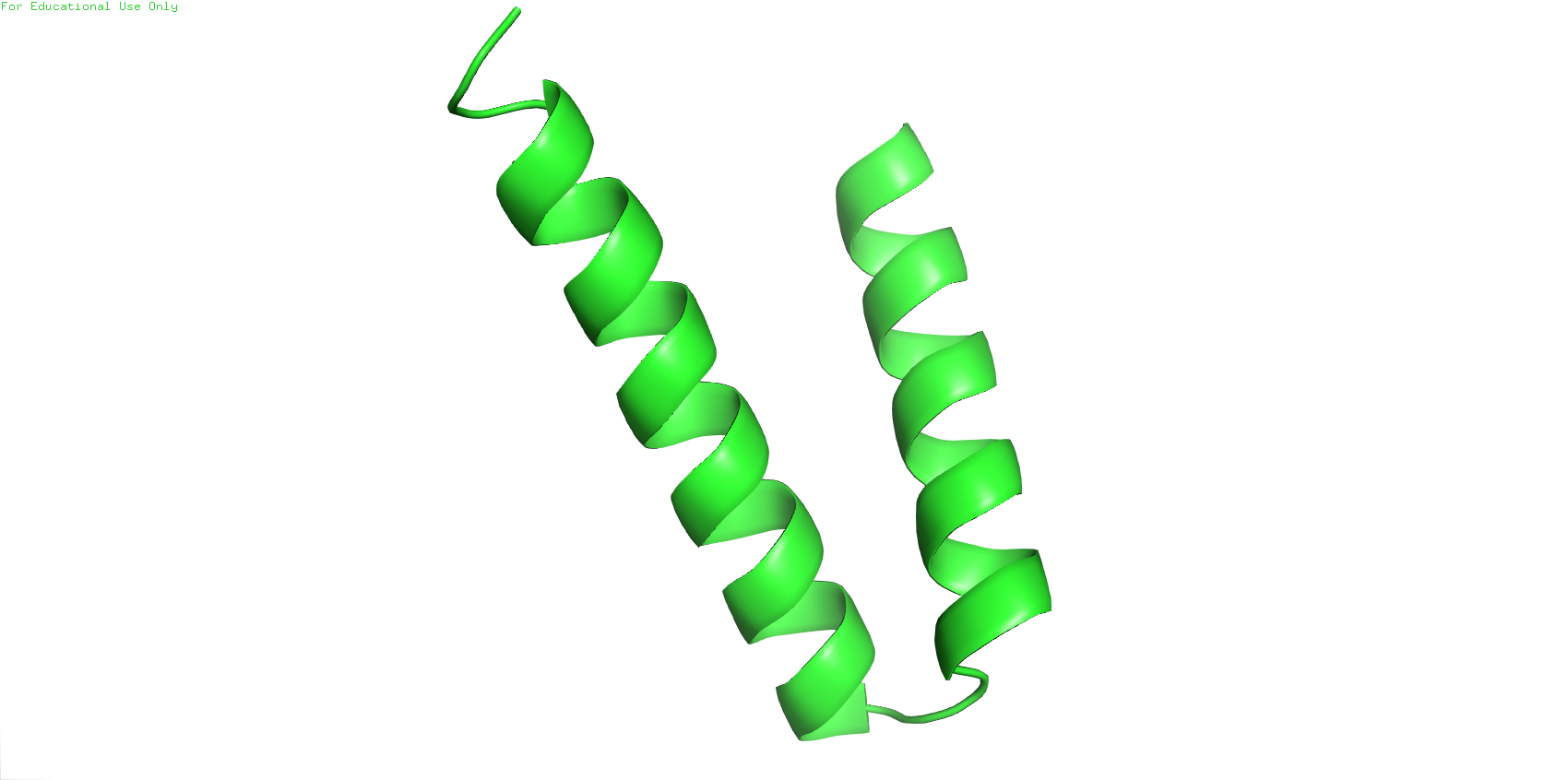
**

**P82 P83**

**
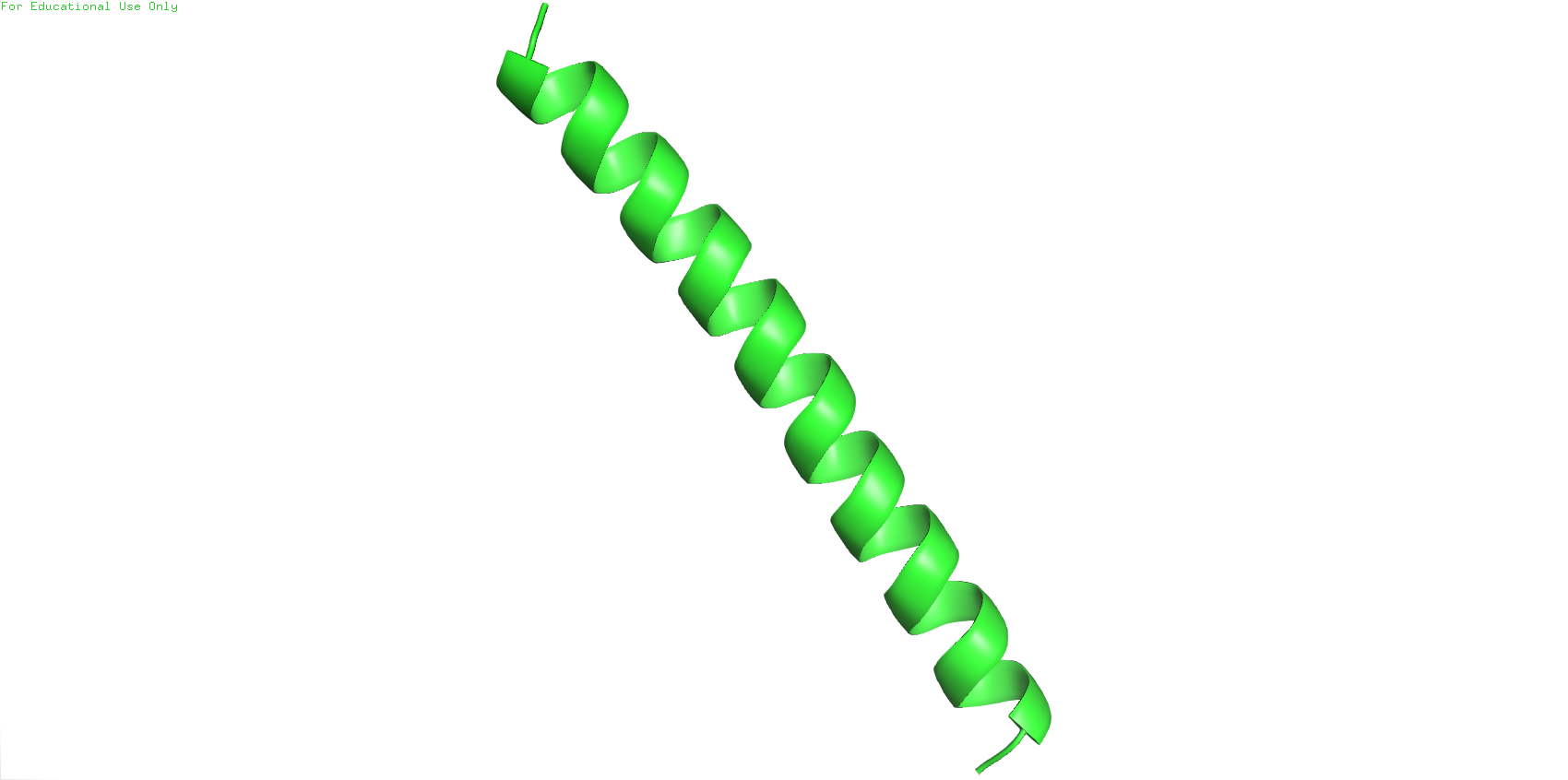

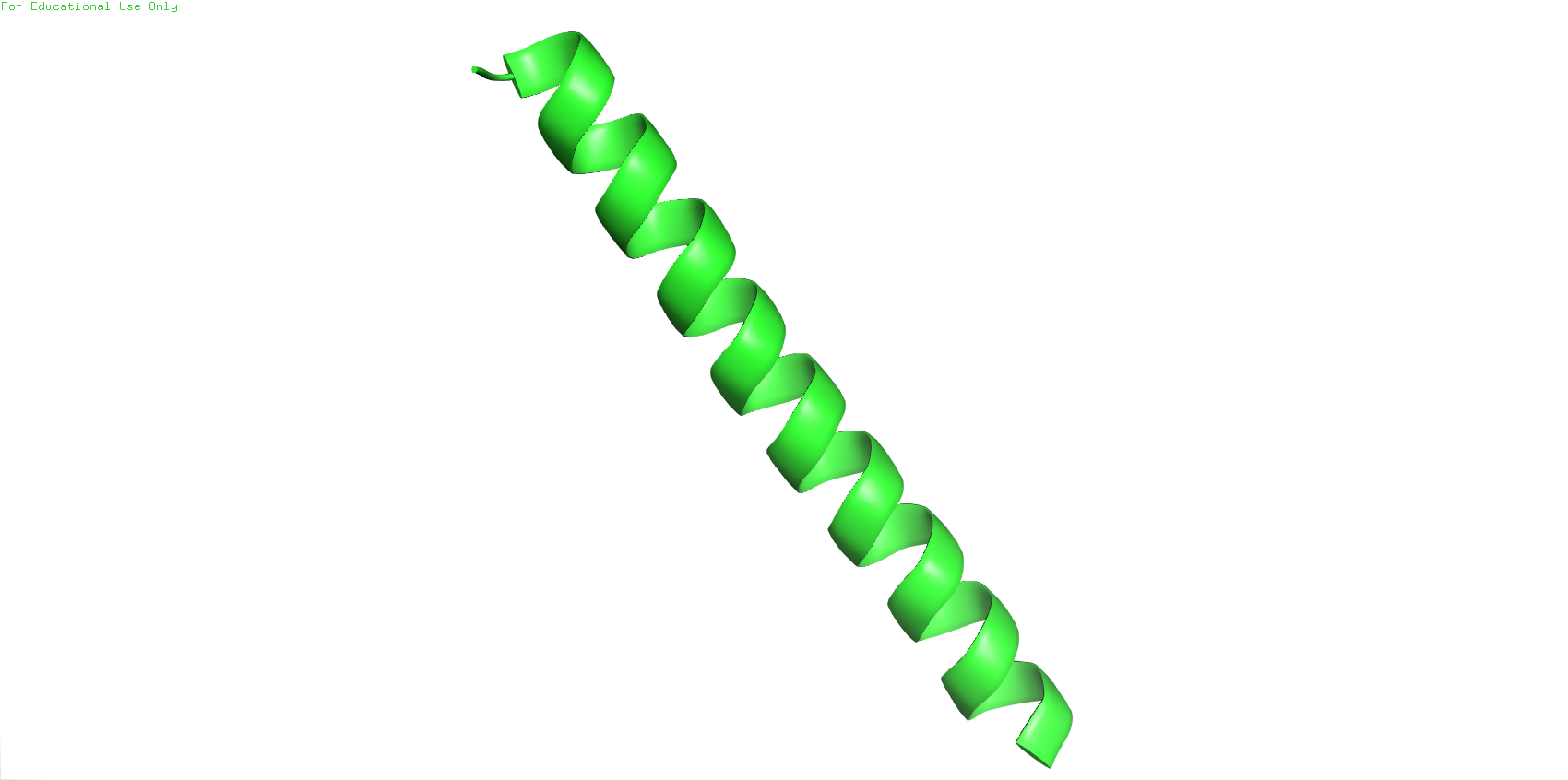
**

**P84 P85**

**
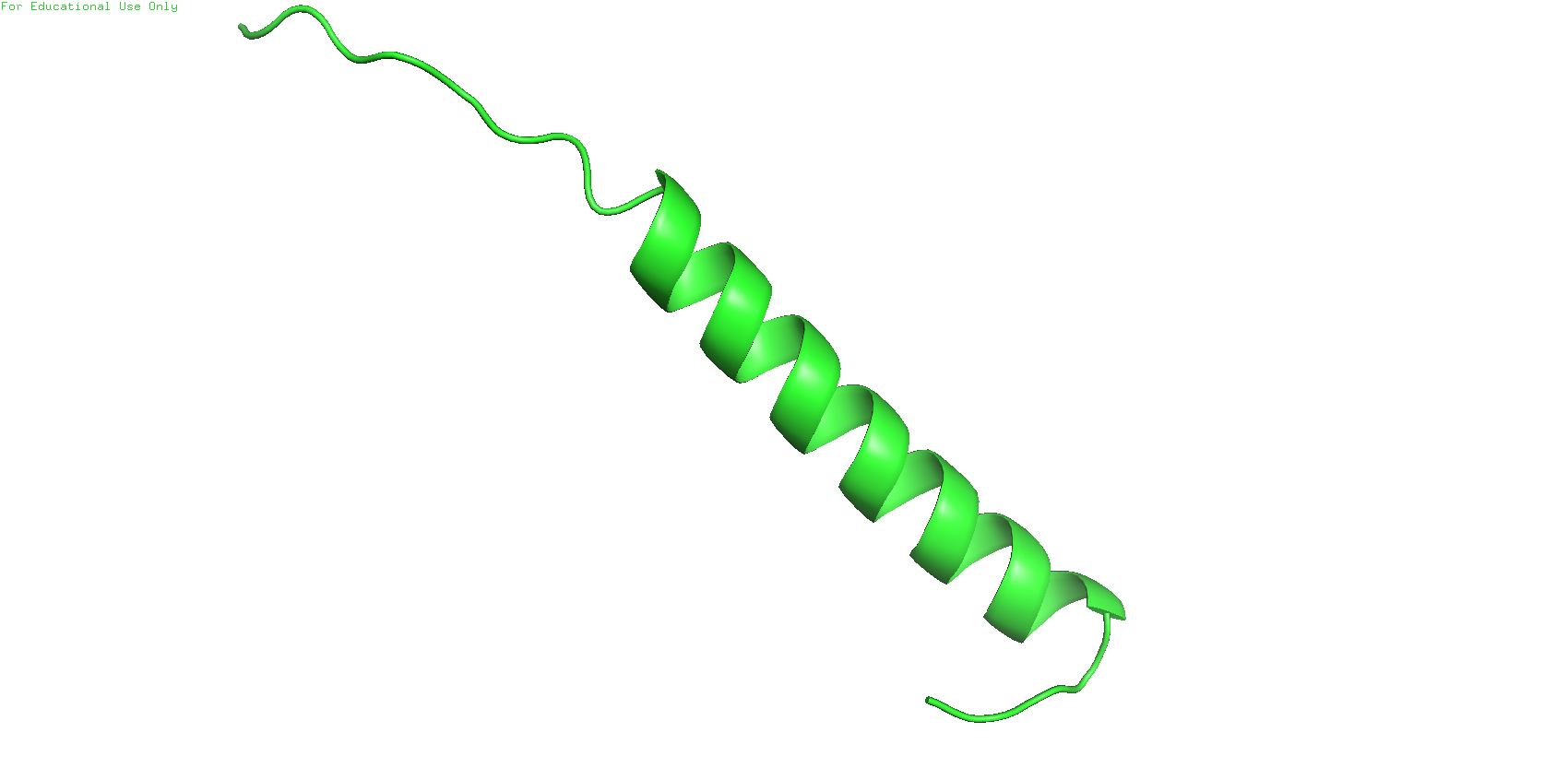

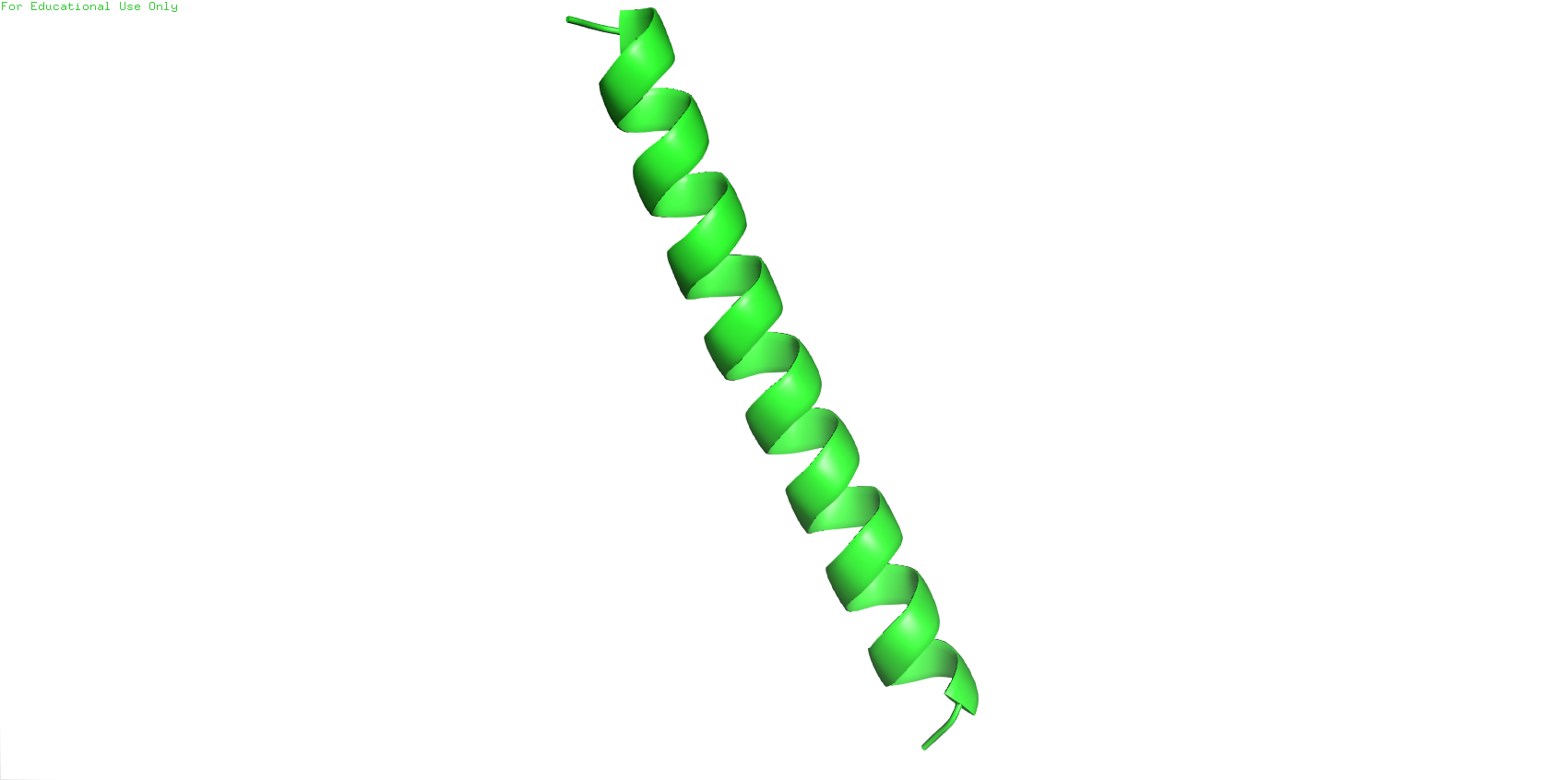
**

**P86 P87**

**
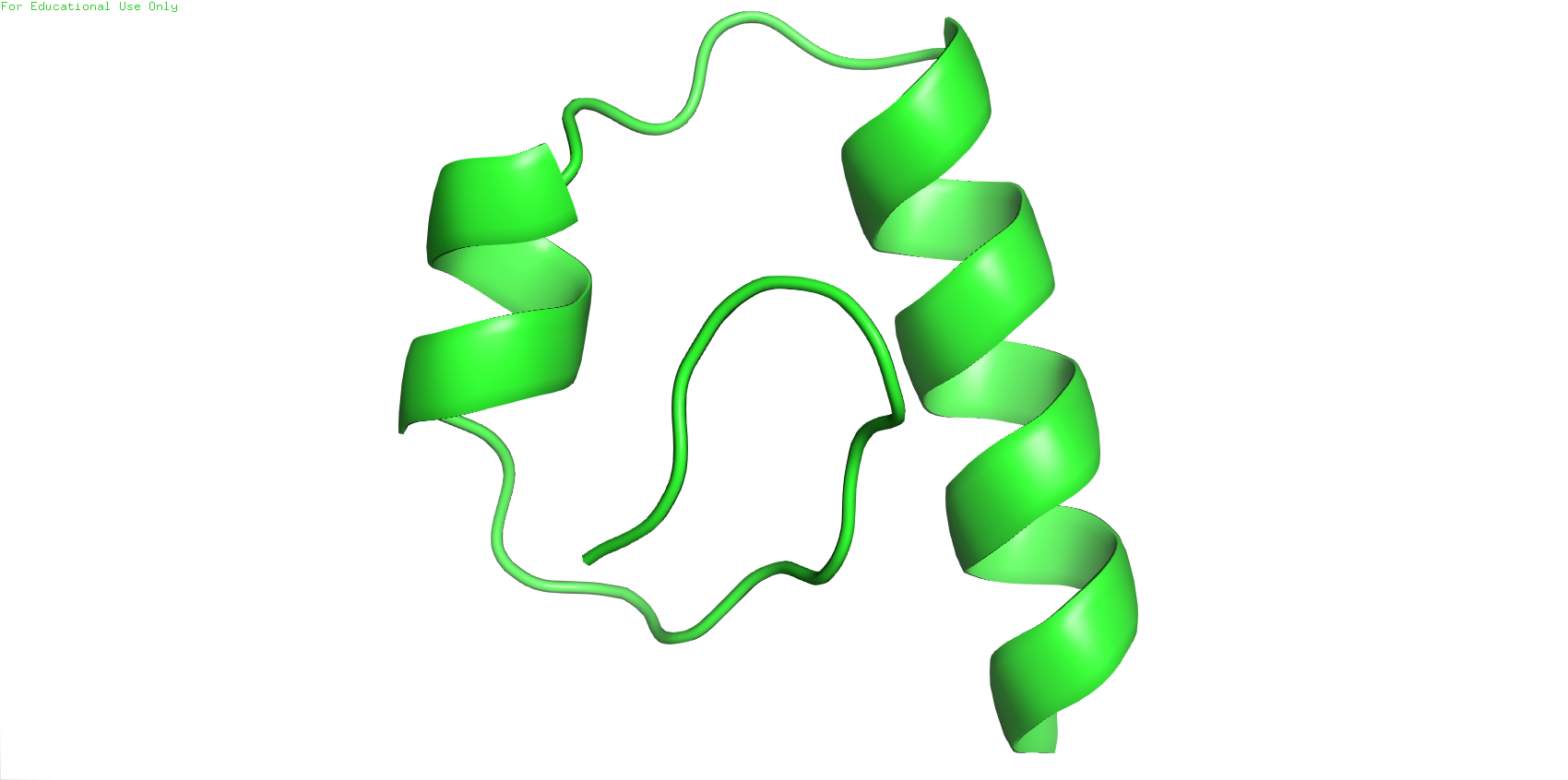
**

**P88**

**Supplementary Figure 1.** The PEP-FOLD peptide sequences of all the peptides.

## Supplementary Tables

**TABLE S1** The docking scoring of all the peptides based on binding energy. The binding energy was calculated through FireDock and Cluspro program.

| **Peptide ID** | **Sequence** | **FireDock score**  **(Global Energy)** | **Cluspro score** |
| --- | --- | --- | --- |
| P1 | MWKTPTLKYFGGFNFSQI | -55.67 | -786.5 |
| P2 | ATAGWTFGAGAALQIPFAMQMAY | -47.06 | -810.7 |
| P3 | GYHLMSFPQAAPHGVVFLHVTW | -41.70 | -998.5 |
| P4 | GVFVFNGTSWFITQRNFFS | -40.28 | -948.0 |
| P5 | NEVAKNLNESLIDLQELGKYEQYIKWPWYVW | -34.40 | -875.9 |
| P6 | AACEVAKNLNESLIDLQELGKYEQYIKW | -58.62 | -775.5 |
| P7 | GNHILSLVQNAPYGLYFIHFSW | -46.09 | -876.4 |
| P8 | GYFVQDDGEWKFTGSSYYY | -46.19 | -762.7 |
| P9 | FKLPLGINITNFRAILTAFS | -32.62 | -815.8 |
| P10 | PTTFMLKYDENGTITDAVDC | -19.74 | -609.4 |
| P11 | VLYNSTFFSTFKCYGVSATK | -40.54 | -801.0 |
| P12 | PALNCYWPLNDYGFYTTSGI | -41.50 | -786.8 |
| P13 | RDVSDFTDSVRDPKTSEILD | -26.89 | -624.1 |
| P14 | YQDVNCTDVSTAIHADQLTP | -58.45 | -885.9 |
| P15 | SNNTIAIPTNFSISITTEVM | -29.82 | -643.2 |
| P16 | QYGSFCTQLNRALSGIAAEQ | -27.81 | -681.9 |
| P17 | GIGVTQNVLYENQKQIANQF | -33.03 | -657.1 |
| P18 | IQKEIDRLNEVAKNLNESLI | -2.92 | -646.4 |
| P19 | GINASVVNIQKEIDRLNEVAKNLNESLIDLQELGKYE | -34.37 | -853.5 |
| P20 | GINASVVNIQKEIDRLNEVAKNLNESLIDL | -26.18 | -745.7 |
| P21 | GINASVVNIQKEIDRLNEVAKNL | -15.05 | -582.5 |
| P22 | QKQIANQFNKAISQIQESLTTTSTALGKLQDVVNQNAQALNTLVKQ | -31.17 | -772.8 |
| P23 | QNQSANQFQKEISQINEVLTTTNTSLGKLQDDVNQNNQSLNTLQKE | -21.48 | -696.9 |
| P24 | KKKKYRNIRRPG | -14.93 | -659.0 |
| P25 | ISGINASVVNIQEEIKKLNEEAKKLNESLIDLQEL | -24.86 | -757.4 |
| P26 | ISGINASVVNIQKEIDRLNEVAKNLNESLIDLQEL | -16.55 | -904.6 |
| P27 | IEEINKKVEEIQKKIEELNKKAEELNKKLEELQKK | -23.00 | -601.4 |
| P28 | TLKPIFKLPLGINITNFR | -43.54 | -908.8 |
| P29 | ISGINASVVNIQKEIDRLNEVAKNLNESLIDLQEL | -43.43 | -791.9 |
| P30 | YENQKQIANQFNKAISQIQESLTTTSTA | -8.85 | -765.5 |
| P31 | DVDLGDISGINASVVNIQKEIDRLNEVAKNLNESLIDLQELGKYEQYI | -21.79 | -772.0 |
| P32 | IQESLTTTSTALGKLQDVVNQNAQALNTLVKQLSS | -28.26 | -671.6 |
| P33 | FGGASCCLYCRCHIDHPNPKGFCDLKGKY | -32.54 | -1004.1 |
| P34 | GGASCCLYCRCH | -34.44 | -800.7 |
| P35 | DLKGKYVQIP | -42.17 | -565.0 |
| P36 | NCVKMLCTHTGTGQAITVTP | -40.99 | -773.4 |
| P37 | PTTCANDP | -34.27 | -476.6 |
| P38 | HVTTTFAPPPPR | -44.59 | -642.2 |
| P39 | SVVPSKATWGFA | -59.18 | -839.2 |
| P40 | FKPSSPPSITLW | -42.51 | -693.7 |
| P41 | ALNCYWPLNDYGFYTTTGIGYQPYRVVVLSFEL | -55.13 | -888.4 |
| P42 | YKYRYL | -30.46 | -729.5 |
| P43 | CANLLLQYGSFCTQLNRALSGIA | -66.85 | -824.9 |
| P44 | PSSKRFQPFQQFGRDVSDFT | -47.93 | -751.4 |
| P45 | RNTREVFAQVKQMYKTPTLKYFG | -39.01 | -831.6 |
| P46 | TKFPSVYAWERKKISNCVAD | -49.90 | -712.4 |
| P47 | KGIYQTSNFRVVPSGDVVRF | -33.76 | -848.3 |
| P48 | KSNVVRGWVFGSTMNNKSQS | -28.74 | -970.7 |
| P49 | TDAVDCSQNPLAELKCSVKSF | -44.45 | -768.9 |
| P50 | TSSMRGVYYPDEIFRSDTLYL | -32.33 | -775.8 |
| P51 | YKGYQPIDVVRDLPSGFNTL | -33.02 | -662.4 |
| P52 | YNYKYRYLRHGKLRPFERDI | -34.59 | -781.0 |
| P53 | NGIGVTQNVLYENQKQIANQFNKAISQIQESLTTTSTA | -32.05 | -912.2 |
| P54 | IQKEIDRLNEVAKNLNESLIDLQELGK | -9.84 | -630.6 |
| P55 | FNKAISQIQESLTTTSTALGKLQDVVNQNAQALNTLVKQL | -15.26 | -725.6 |
| P56 | ALNTLVKQLSSNFGAISSVLNDILSRLDKVEAEVQIDRL | -30.75 | -763.2 |
| P57 | IDRLITGRLQSLQTYVTQQLIRAAEIRASANLAATK | -27.51 | -799.9 |
| P58 | YVTQQLIRAAEIRASANLAATKMSECVLGQSKRVDFCGKG | -37.17 | -826.2 |
| P59 | VYDPLQPELDSFKEELDKYFKNHTSPDVDLGDISGINASVV | -37.92 | -803.9 |
| P60 | QPELDSFKEELDKYFKNHTSPDVDLGDISGINASVVNIQ | -54.18 | -659.1 |
| P61 | SFKEELDKYFKNHTSPDVDLGDISGINASVVNIQKEIDRLNE | -26.50 | -654.7 |
| P62 | LDKYFKNHTSPDVDLGDISGINASVVNIQKEIDRLNEVAK | -28.76 | -691.9 |
| P63 | FKNHTSPDVDLGDISGINASVVNIQKEIDRLNEVAKNLNES | -22.36 | -782.4 |
| P64 | PDVDLGDISGINASVVNIQKEIDRLNEVAKNLNESLIDLQ | -33.39 | -752.3 |
| P65 | GDISGINASVVNIQKEIDRLNEVAKNLNESLIDLQELGKY | -17.78 | -840.4 |
| P66 | ISGINASVVNIQKEIDRLNEVAKNLNESLIDLQELGKYEQYI KWPW | -27.82 | -761.0 |
| P67 | ISGINASVVNIQKEIDRLNEVAKNLNESLIDLQELGK | -17.43 | -859.7 |
| P68 | ISGINASVVNIQKEIDRLNEVAKNLNESLIDLQEL | -37.26 | -912.6 |
| P69 | GINASVVNIQKEIDRLNEVAKNLNESLIDLQELGK | -32.26 | -788.5 |
| P70 | INASVVNIQKEIDRLNEVAKNLNESLIDLQELGKYEQYIKWPW | -35.77 | -823.5 |
| P71 | INASVVNIQKEIDRLNEVAKNLNESLIDLQELGKYEQYI | -40.94 | -842.1 |
| P72 | INASVVNIQKEIDRLNEVAKNLNESLIDLQELGK | -32.33 | -724.9 |
| P73 | INASVVNIQKEIDRLNEVAKNLNESLIDL | -26.47 | -849.4 |
| P74 | VVNIQKEIDRLNEVAKNLNESLIDLQELGKYEQYIKWPW | -59.16 | -926.3 |
| P75 | VVNIQKEIDRLNEVAKNLNESLIDLQELGK | -14.45 | -750.3 |
| P76 | IQKEIDRLNEVAKNLNESLIDLQELGKYEQYIKWPW | -19.25 | -791.3 |
| P77 | IDRLNEVAKNLNESLIDLQELGKYEQYIKWPW | -30.96 | -740.6 |
| P78 | TSPDVDLGDISGINASVVNIQKEIDRLNEVAKNLNESLIDLQELGKYE | -42.36 | -984.0 |
| P79 | VDLGDISGINASVVNIQKEIDRLNEVAKNLNESLIDLQELGKYE | -49.12 | -811.9 |
| P80 | DISGINASVVNIQKEIDRLNEVAKNLNESLIDLQELGKYE | -43.91 | -836.4 |
| P81 | DLSLDFEKLNVTLLDLTYEMNRIQDAIKKLNESYINLKE | -38.42 | -796.5 |
| P82 | NQNAQALNTLVKQLSSNFGAISSVLNDILSRLDKVEAEVQIDRLIT | -32.66 | -719.4 |
| P83 | GVTQNVLYENQKQIANQFNKAISQIQESLTTTSTALGKLQ | -21.82 | -807.0 |
| P84 | LTTTSTALGKLQDVVNQNAQALNTLVKQLSSNFG | -24.24 | -694.7 |
| P85 | KQLSSNFGAISSVLNDILSRLDKVEAEVQIDRLITG | -35.84 | -779.1 |
| P86 | GRLQSLQTYVTQQLIRAAEIRASANLAATKMSEC | -41.59 | -877.2 |
| P87 | GINASVVNIQKEIDRLNEVAKNLNESLIDLQELGKYE | -27.63 | -841.9 |
| P88 | KEIDRLNEVAKNLNESLIDLQELGKYEQYIKWPWYVW | -24.69 | -777.3 |

**TABLE S2** The physiochemical properties of the best 15 peptides molecules.

| **Peptide**  **ID** | **Number of amino acids** | **Molecular weight** | **Theoretical pI** | **Total number of negatively charged residues (Asp + Glu)** | **Total number of positively charged residues (Arg + Lys)** | **Total number of atoms** |
| --- | --- | --- | --- | --- | --- | --- |
| P2 | 23 | 2374.76 | 5.57 | 0 | 0 | 327 |
| P3 | 22 | 2494.90 | 7.02 | 0 | 0 | 346 |
| P4 | 19 | 2254.53 | 9.75 | 0 | 1 | 310 |
| P7 | 22 | 2576.94 | 6.92 | 0 | 0 | 359 |
| P14 | 20 | 2191.35 | 3.93 | 3 | 0 | 296 |
| P28 | 18 | 2085.56 | 11.17 | 0 | 3 | 313 |
| P39 | 12 | 1249.43 | 8.47 | 0 | 1 | 177 |
| P41 | 33 | 3864.39 | 4.37 | 2 | 1 | 535 |
| P43 | 23 | 2456.86 | 8.06 | 0 | 1 | 345 |
| P71 | 39 | 4518.10 | 4.49 | 7 | 4 | 645 |
| P74 | 39 | 4730.40 | 4.77 | 7 | 5 | 674 |
| P78 | 48 | 5270.83 | 4.11 | 10 | 4 | 745 |
| P79 | 44 | 4870.45 | 4.21 | 9 | 4 | 693 |
| P80 | 40 | 4486.01 | 4.33 | 8 | 4 | 638 |
| P86 | 34 | 3723.28 | 9.31 | 2 | 4 | 528 |
